# Mancala board games and origins of entrepreneurship in Africa

**DOI:** 10.1371/journal.pone.0240790

**Published:** 2020-10-15

**Authors:** Maxwell Mkondiwa

**Affiliations:** Center for Agricultural Research and Development (CARD), Lilongwe University of Agriculture and Natural Resources (LUANAR), Lilongwe, Malawi; University of West London, UNITED KINGDOM

## Abstract

This study examines the correlational relationship between the historical playing of indigenous strategic board games (also called mancala) and the socio-economic complexity of African ethnic groups as well as the incidence of entrepreneurial pursuits. Anthropology literature suggests that these games may be associated with socio-economic complexity of the ethnic groups—the so-called games in culture hypothesis. I revisit this hypothesis with better data and motivated by anecdotal evidence, introduce a contemporary hypothesis, origins of entrepreneurship hypothesis—that descendants of societies that played complex mancala games are more likely to be engaged in non-farm self-employment today. I compile the first comprehensive database of mancala games in Africa matched to ancestral characteristics data, and for 18 African countries, to the Afrobarometer survey data. Using historical and contemporary data, I do not find evidence for either hypothesis. Despite the null results, I explore how related hypotheses and studies can build on the comprehensive mancala database.

## Introduction

A growing literature in economics investigates the short run and long run effects of culture on economic development (see a review of this literature by Nunn [1]). Beyond cultural traits related to work or anti-social beliefs (e.g., plough agriculture by Alesina, Giuliano and Nunn [2], and witchcraft beliefs by Gershman [3]), the recent literature has rightly started to focus on the intrinsic structures of African communities by understanding the origins and consequences of these features. Examples include: Moscona, Nunn and Robinson [4] on family structure and conflict; Chen [5] on language characteristics and saving; Lowes [6] on marriage systems and child outcomes; and Xue and Michalopoulos [7] on folklore and trade. In this paper, I introduce and analyze the correlational relationship between another non-work intrinsic feature of African ethnic groups, i.e., the playing of indigenous board games (commonly called mancala) and economic outcomes—socio-economic complexity and entrepreneurship—in the historical and contemporary societies.

The premise for testing these hypotheses is that the rules and strategies in the mancala board games are consistent with everyday language and decisions [8]. Board games can therefore act as catalysts for heuristics for decision making in real life. This idea also follows from Simon’s [9] research program on procedural and bounded rationality in which given their complexity, combinatorial tasks such as board games offer an ideal environment for exploring human bounded rationality [10, p.38] and learning [11, 12]. This study investigates whether the learning in the use of heuristics from playing strategic board games influences some societies to be socio-economically complex (in terms of settlement patterns and jurisdictional hierarchy beyond the community) and to be good at business.

Taking Nunn’s [1], S109 definition of culture as *“decision making heuristics or ‘rules of thumb’ that have evolved given the need to make decisions in complex and uncertain environments”*, this study contributes to this emerging literature by focusing on the foundations of the ‘rules of thumb’. In cognitive science and psychology (for example, the works of Adrianus de Groot, Herbert Simon, Fernand Gobet and Gerd Gigerenzer), strategic board games—two-person zero-sum games of perfect information—especially chess have been used to understand how human beings use heuristics to make decisions in complex environments. Building on this understanding, I analyze two hypotheses related to the playing of indigenous strategic board games in African societies: *games in culture hypothesis* and *origins of entrepreneurship hypothesis*. *Games in culture hypothesis* [13]—that societies that play strategic games are more likely to engage in complex social and economic activities—has generated a contentious debate in anthropology but so far there is no comprehensive empirical research on it. The hypothesis has been extended to cross-cultural relationships between strategic game playing and the following: obedience training [14], use of strategic folktales [15], and political complexity [16]. The hypotheses are documented in the Human Relations Area Files (https://hraf.yale.edu/ehc/hypotheses/1365).

The controversies of the games in culture hypothesis can be emotional especially because society complexity is subjective and at best incomprehensible. A closer look at the variables used by Roberts and co-authors shows that what they refer to as social or political complexity is simply whether the ethnic group had political jurisdictions beyond a community. Likewise, economic complexity was defined by levels of settlement patterns. It is questionable whether these variables reflect social-economic complexity of an ethnic group. Nonetheless, echoes of the games in culture hypothesis appear in economic history, political science and economics. For example, a prominent political scientist and economic historian, James Robinson, citing Iliffe [17, p.99] related the egalitarian African culture as reflected in mancala with its equal sized seeds and aiming to capture the opposing pieces to one’s own compared to chess developed in other cultures with its hierarchy of pieces and the objective of destroying the opposing forces. He presented this observation at a seminar talk at the United Nations University World Institute for Development Economics Research (UNU-WIDER) on 22^nd^ March 2019 in Helsinki (audio available here: https://www.wider.unu.edu/sites/default/files/Events/Video/27032019-Robinson.mp3). This generalization of African cultures and the educational content of mancala games is nonetheless questionable without a comprehensive empirical analysis.

Why is testing the games in culture hypothesis important? The first reason is the positive correlation between socio-political complexity (jurisdictional hierarchy beyond community) and other precolonial institutions and the contemporary economic development in Africa [18–21]. The games in culture hypothesis allows an investigation of a potential reason for the variation in socio-political complexity. In addition, similar expressive models of culture like folktales have been found to be associated with various economic outcomes including participation in trade [7, p.22]. In much earlier anthropological literature, presence of games of strategy was found to be associated with presence of folktales of strategy [15]. Second, in a recent study, Mkondiwa [8] demonstrated how the rules and words used in mancala games are the same as the rules and words used in everyday economic decision making thereby elevating mancala games as essential tools for understanding economic decision making in most of sub-Saharan Africa. This is echoed by a comprehensive analysis by Bayeck [12] of the variants of mancala in Cameroun where she found that practices within gameplay intersect with life beyond the gameplay environment.

**Fig 1:**
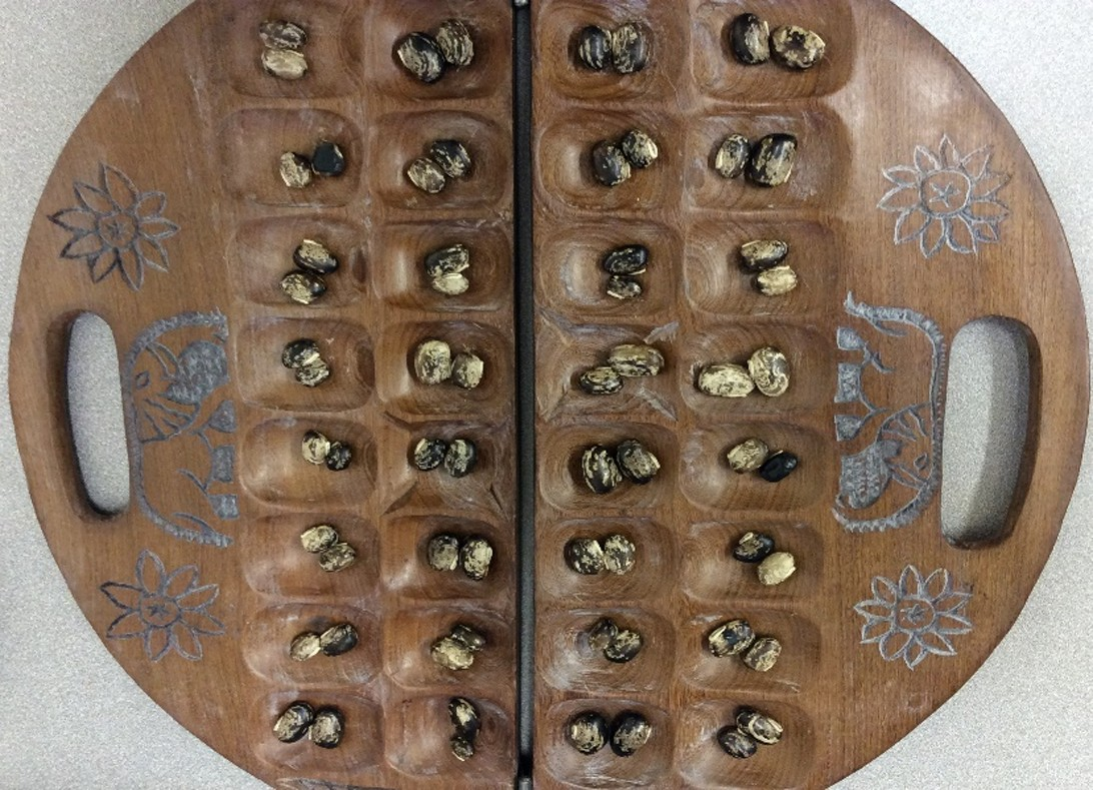
Example of a 4 by 8 mancala board game (Bao). Source: Author.

In addition to testing the games in culture hypothesis, I introduce a new hypothesis—*origins of entrepreneurship—*based on observation of contemporary societies that substantially lean towards entrepreneurial activities more than others and it begs the question whether strategic games may be related to incidence of entrepreneurial pursuits. Such societies appear to be in each of the African countries. Examples of large African societies known for entrepreneurial pursuits with members who play complex mancala (e.g., *Bao*, see Fig 1 for an example of the board game) include the Yao in Malawi and Mozambique, and the Swahili in Tanzania. The *origins of entrepreneurship* hypothesis is particularly important in Africa because strategic board games are prevalent and have significant variation in the levels of complexity and rules. In addition, anecdotal evidence supports the view that mancala games were intended originally to be a means of keeping business transactions—it is an image of the act of buying and selling [22]. In the case of the Swahili, *Bao* also reflected the Swahili ethic especially regarding ‘*financial commitments to the community and showing business acumen and an understanding of the importance of the long term in financial matters’*. [23].

Why is testing the origins of entrepreneurship hypothesis important? There is a growing literature that focuses on entrepreneurship training in developing countries. This literature has acknowledged the difficulty in predicting who is likely to be a better entrepreneur given a good economic environment [24, 25]. These training interventions are also very expensive and results on their effectiveness are mixed. There is however a subset of these studies focusing on heuristics that have been successful across different settings. Dexler, Fischer and Schoar [26] conducted a randomized control trial to increase financial literacy, comparing formal accounting training against a simple rule-of-thumb training of financial heuristics in Dominican Republic. They found that rule of thumb training significantly improved financial literacy especially for micro-entrepreneurs. Arraiz, Bhanot and Calero [27] conducted a similar randomized control trial in Ecuador in which they compared the effectiveness of a traditional training program and a tailor-made training program based on heuristics—practical approaches to problem solving—for microentrepreneurs. They found that heuristics training had statistically significant and economically meaningful impacts on sales and profits relative to the traditional training. This points to the need to establish different heuristics that might work in different contexts. Several recent studies in the pedagogy of entrepreneurship training have also suggested games—commonly referred to as serious or simulation games—as a successful approach to training entrepreneurs. None of these studies or the large number of randomized control trials in Africa have tested mancala games; yet the games mimic business transactions. In this paper, I focus on the heuristics that may be learned from the playing of mancala games. There are hundreds of variants of mancala games in Africa. This paper deals with all these variants differentiated by the mode of play, ethnic groups and geographical spread. This line of research can open research for investigating the non-traditional mechanisms for improving entrepreneurship.

Using historical and contemporary data, I do not find evidence to support either the games in culture or the origins of entrepreneurship hypotheses. These results are especially salient when I include country fixed effects. As others have already criticized the logic of the games in culture hypothesis, these results reflect how controversial the hypothesis is even in cases where the quality of the mancala data is relatively good. Perhaps, the results in this paper reflect the intensive margin, i.e., game type complexity of the hypothesis, yet the actual hypothesis itself was concerned with the extensive margin—presence and absence of games of strategy. The difficulty of testing the extensive margin is that it is difficult to determine which ethnic groups or societies lack games of strategy [28]. On the origins of entrepreneurship, it is important to recognize that both economic historians, anthropologists, economists and social scientists in general do not have a compelling theory of the origins of entrepreneurial ability. I however find a ‘peer effect’ of 2A and 4A (assumed complex in the game ethnology literature) mancala playing among immigrants implying a business social network which is synonymous with a key distinguishing feature of these mancala games—captured seeds are fed into own holes. Perhaps future research should directly test using randomized control trials an entrepreneurship curriculum that embeds known business-related mancala like *Bao*. The research can be along the lines of the heuristic or rule of thumbs entrepreneurship trainings [26, 27].

### Related literature

This paper is related to the two strands literature corresponding to the games in culture and origins of entrepreneurship hypothesis. First, the games in culture hypothesis remains the most debated anthropology hypothesis regarding games. In recent anthropological work, Chick [29] defends the usefulness of the hypothesis while De Voogt [28, 30] questions both the reasoning behind it, the data and the empirical methods used. De Voogt suggests that though the cross-cultural comparative method remains a viable method of research, the present databases (especially those used by Roberts, Arth and Bush [13] and Chick [34]) do not contain systematic data on games. Other game anthropologists especially Phillip Townshend were also against the cross-cultural correlations. Nonetheless, game anthropologists have also posited other related hypotheses regarding strategic board games in culture though without explicit quantitative analysis. For example, Pankhurst [31, p.28] suggests a relationship between game moves and whether the society is pastoral or agricultural.

The common feature of all the studies on either side of the debate is the lack of substantial empirical evidence. The correlational analyses that confirmed the hypothesis [13, 32–34, 16] were based on simply classifying ethnic groups into those that played strategic games and whether their political and economic organization were complex (i.e., political jurisdiction was beyond community and settlement patterns were complex). This approach did not control for other characteristics of the societies including their location, distance to coast, migration and religious beliefs; nor did it take into consideration the varying complexity and rules of games of strategy. Beginning with the classic paper that introduced the hypothesis to the current literature, these debates rely on very small samples (e.g., 50 ethnic groups across the world). Even a study by Chick [34] which increased the sample size only covered 107 games across the world of which only 37 were games of strategy. The critics rely on rhetoric of counter-examples which is also unhelpful to the debate. In this paper, I expand the number of African ethnic groups playing strategic games in the matched sample to 102 ethnic groups thereby allowing more nuanced multivariate regression analyses.

Second, this study contributes to the economics literature on the classification of occupation by ethnic groups, nationality or language. This line of work is associated with the classic Weber [35] on protestant work ethic and capitalism [36]. So far, many studies have found that minorities and immigrants are more entrepreneurial than locals in many societies (see for example, chapters in Ochonu [37] and Jalloh [38] on Fula migrant merchants). There are reasons for entrepreneurial differentiation including risk aversion, religion, social networks and trust. In Switzerland, Erhardt and Haenni [39] found that individuals with ancestry from the German speaking side are more entrepreneurial (start 20% more firms) than those from the French speaking side even though they live in same municipality today. They attributed this to differences in risk aversion across the groups.

Bottinici and Ekstein [40] document the transition of majority of Jews from farming just as many ethnic groups in the eighth-ninth centuries CE to skilled and urban occupations. They attribute this evolution to widespread literacy prompted by a religious and educational reform in Judaism. A similar human capital argument is made by Becker and Woessmann [36] on the protestant entrepreneurship. In India, Iyer and Schoar [25] investigated the cultural determinants of entrepreneurship motivated by observations of the Marwaris in India, Svabians in Germany, and Esfahanis in Iran. They selected entrepreneurs from three different groups in the South Indian city of Chennai: Andhraites, Marwaris, and Tamilians. They found that the Marwaris, a large minority that is also commonly considered an entrepreneurial community have distinctively different business practices. In a randomized experiment, the Marwaris charge lower prices for a homogeneous product within the same market to build business relationship for the future. In the United States (USA), Kerr and Mandorff [41] investigated the relationship between migrants’ ethnicity, occupational choice, and entrepreneurship. Specifically, they developed a model of social interactions in which non-work relationships facilitate acquisition of sector-specific skills to justify the observed patterns in self-employment, e.g., that concentration of Korean self-employment in dry cleaning is 34 times than other immigrants.

The puzzle remains in understanding how certain societies attained the skills for entrepreneurship. Several cultural and contextual factors have been proposed [37]. Consider for example, the Kikuyu in Kenya: Alkan [42] suggests their geographical location, half way between the coast and the interior may be the reason while Marris [43] attributed the Kikuyu entrepreneurship to being in detention camps during the emergency. Anthropological and historical literature on mancala board games has speculated several uses of the different games in different societies including initiation of young people, funeral rituals and selection of traditional leaders [44]. It is thus very easy to spuriously attribute the board games to many sociopolitical and economic activities of the people. To avoid these spurious results, it is important to state clearly the theoretical model, the hypotheses, the sources of data, and types of analyses to conduct.

## Theories and hypotheses

### Theories

The bedrock of the games in culture and origins of entrepreneurship hypotheses is the understanding of the role of games in cultural values training and decision making. I first synthesize theories that explain the cultural values training role and then those that pertain to decision making. The cultural values training theories are based on cultural evolution in anthropology while decision making theories are from cognitive science, psychology and behavioral economics.

Cross-cultural research relies on observing different cultures and generating generalizable patterns of causal factors. I rely on cross-cultural research by John Roberts that posed the games in culture hypothesis. I however extend the hypothesis which was at extensive margin to intensive margin. I place the games in culture hypothesis in a notion of culture in evolutionary anthropology [45, 46] and economic history of economic development [1] that views culture as decision making rules of thumb. This view is still inadequate in that it assumes culture as a given thereby neglecting a deeper look at the origins of the different aspects of culture. Games of strategy in culture are hypothesized to be key sources of rules of thumb observed across societies. This new view at culture goes beyond cross-cultural evidence to investigate what makes games salient in culture. Here, I build on psychological literature and behavioral game theory literature pioneered by Herbert Simon that relates economic games to economic outcomes.

The human decision-making model of Herbert Simon, the bounded rationality model, essentially uses the process of decision making in games of strategy (Herbert Simon used examples of Chess) to justify that humans do not conduct all computations before making a decision. Other related behavioral game theoretic models like *k*-level thinking [47] and cognitive hierarchy [48] all point to aspects of mancala game play that are common in everyday decision making. Strategic games in culture are therefore a good training in this essential process of decision making. Evidence of behavioral games in the field provide further evidence that behavior in games can predict economic outcomes in real life. It is thus natural to assume that the behavior in games in culture is a strong predictor of economic outcomes. It is important to state that the expressive models of cultural values do not start and end with strategic games; they permeate into several activities including riddles [49], folklores [7], dances, proverbs [50–52], and special ceremonies.

How does all this relate to contemporary entrepreneurship skills among African societies? Here I refer to Boyd and Richerson [46] who conceive societies as heirs to socially transmitted information. It is common to see business owners among sons and daughters of business owners and this applies to most professions (e.g., doctors, teachers and soldiers). According to the Bayesian rational choice theory of traditions as advanced by Boyd and Richerson, each individual either imitates a tradition or updates it using own experience. The imitation can be from parents, teachers and so on. In this Bayesian terminology, it means that prior beliefs probably obtained from parents or society are updated to form some posterior beliefs or actions that we observe as culture. But how did the parents gain those beliefs and why are they different from other groups? In this paper, I suggest that ‘hyperparameters’, borrowing from Bayesian terminology, which in this case is playing of strategic games or use of trade related folk roles, are key to the prior beliefs that a member of a society possesses. What this means is that the training that accorded some groups skills to strategically engage in trading from the playing of complex strategic games distinguished them from other groups at the outset. Placing this within the Bayesian rational choice model implies that if the playing of complex mancala board games allowed a society to distinguish themselves in economic activities like trading, the next generations developed the trading abilities that have overtime become the working life of those communities.

This conjecture may however break down if all societies developed the same faculties towards commerce and if games of strategy did not play a role to this distinction. One may list a whole load of other activities—religion, family structure, dances—that shaped societies to be engaged in certain economic activities. These factors are illustrated in historiographies of entrepreneurial Africans (Ochonu 2018). Beyond imitation, individuals in societies that play strategic games may also be more connected. Several studies have documented that venues where mancala games are played become important sources of information as such stronger social and economic networks may easily emerge in such society, thereby encouraging more economic activity and entrepreneurship.

### Hypotheses

#### Hypothesis 1 [Games in culture]

Societies that traditionally played complex mancala board games are associated with complex social political and economic structures.

*Main outcome variable*. The *economic complexity* variable is constructed from the ethnographic atlas provided by Giuliano and Nunn [53]. The variable is v30: settlement patterns. The categories in the data include: 1 = missing data, 2 = nomadic or fully migratory, 3 = seminomadic, 4 = semisedentary, 5 = compact or impermanent settlements, 6 = neighborhoods of dispersed family homes, 7 = dispersed hamlets forming a single community, 8 = compact and relatively permanent, and 9 = complex settlements. The categories 8–9 is designated complex with a value of 1 and the other categories (excluding missing data category) are denoted not complex with a value of 0. This variable is also commonly referred to as settlement pattern complexity.

*Secondary outcome variable*. The *political complexity* variable is constructed from the ethnographic atlas provided by Giuliano and Nunn [53]. The variable is v33: jurisdictional hierarchy beyond local community. The categories include: 1 = missing data, 2 = no levels, 3 = one level, 4 = two levels, 5 = three levels, 6 = four levels. The categories 4–6 is designated complex with a value of 1 and the other categories (excluding missing data category) are designated not complex with a value of 0. This variable is also commonly referred to as political centralization.

*Independent variable of interest*. Mancala board game complexity is described in section 3.2.

#### Hypothesis 2 [Origins of entrepreneurship]

Individuals from societies that traditionally played complex mancala board games are more likely to move out of agriculture and engage in entrepreneurial activities today. The detailed descriptions of how the variables were created are presented below and in S1 Table.

*Outcome variable*. 0 if the household head is self-employed in non-farm activities, 1 if self-employed fully in agriculture.

*Key assumption*. The underlying assumption for this hypothesis is that all African societies were engaged in agriculture, pastoralism or hunting prior to transitioning into non-agricultural self-employment.

## Data

I use three main sources of data: (i) Ancestral characteristics of modern populations data, (ii) ethnological and anthropological literature on mancala board games and (3) household survey data from the Afrobarometer survey as provided by Nunn and Watchekon [54].

### Ancestral characteristics of modern populations data

I use the ancestral characteristics of modern populations data [53] various versions of which have been used extensively in many cross-cultural studies and its credibility has been documented by anthropologists, economists and other social scientists. The data are available here: https://scholar.harvard.edu/nunn/pages/data-0, https://worldmap.harvard.edu/data/geonode:murdock_ea_2010_3 or https://github.com/sboysel/murdock. The important variables for this study include: name of the society, occupation, incidence of slave trade and location (see S1 Table in the appendices for details). There are 531 African societies in the data. The data has also a variable (v35) which shows categories of games available in a society (none, game of physical skill, game of strategy, game of chance and the combinations) based on evidence from Roberts, Arth and Bush [13]. This variable has missing information for 469 societies as such cannot be used to credibly test the games in culture and origins of entrepreneurship hypotheses. In addition, board game anthropologists (e.g., de Voogt [28]) have already argued that 20% of games information is inaccurate (I do not find evidence of this in the mancala-Murdock matched sample). Other scholars (e.g., Ball [32]) have also argued that genuine measures of association have not been legitimate given the form of the data. I therefore use other sources for the board game information.

### Mancala board games data

The information on mancala game structure for each ethnic group is coded from various papers by archeologists, anthropologists and historians. The most comprehensive of these is by Townshend [55] who provided a complete description of several types of mancala board games played in Eastern and Southern Africa. This source has about two thirds of games that were not documented in the classic Murray [57] compilation of board games other than chess. For each game, the description includes: the general rules of the game, number of rows, number of columns, number of seeds used, allowable directions, the ethnic groups playing the version of the game, the location and country in which the game was played. Other sources included Sanderson [56], Pankhurst [31], Murray [57], and Walker [58].

The data on mancala game structure were used to compute complexity classifications based on the number of pockets, dimensions, number of seeds used, number of directions and average time to complete the game. I considered two categories of measures (i) anthropological classifications and (ii) combinatorial game theory. On anthropological classifications, I used classifications by Murray [57] and Townshend [55]. These include: four row-types (A, B, C and D) and five types of two-row games (A, B, C, D, and E). In combinatory game theory, there are several measures for game complexity including: (i) state-space complexity, (ii) game tree size, (iii) decision complexity, (iv) game tree complexity, (v) computational complexity (https://en.wikipedia.org/wiki/Game_complexity) and (vi) mutational complexity. Gobet, de Voogt and Retschitzki [10] defines state-space complexity as the number of different positions, game tree complexity as the number of leaf positions that can be generated by search—the same position may be counted several times, and mutational complexity as changes on the board and number of pieces involved in a move. The first five measures are commonly used in combinatorial game theory to describe game complexity. Of these, only game tree complexity and mutational complexity are related to complexity humans face when playing the game. Of these measures, game tree complexity could be favourable in future studies because it has explicit formula [59] and can be simulated in complex games.

Table 1 shows the Townshend classification of mancala games, their complexity categories and number of ethnic groups that play the different variations of the game. According to accounts of several anthropologists and economic historians [31, 55, 57], 4A and 2A games are the most complex and have the most varied mode of capture. The huge caveat here is that the subjective westerner’s view of complexity of the games may not reflect the actual complexity of those who play the game. We use these classifications while acknowledging that better measures possibly from computational game theory would be the most appropriate. I dispel the subjectivity argument by grouping mancala games by complexity relatedness after examining game rules and strategies as suggested by Divilly et al [60] and Donkers et al [61]. For example, 4A games like *Bao* (played by Yao and Swahili ethnic groups in Malawi and Tanzania respectively) and *Omweso* (played by the Buganda in Uganda) are the most complex based on the state-space complexity and game-tree complexity from combinatorial game theory and still unsolved [62, 63]. In contrast, *Awari* board game (also called *Wari* or *Awele*), a 2B game played in parts of Ghana (Ashanti ethnic group) and Sierra Leone (Mende ethnic group) has a lower state-space complexity and has been solved. The distinguishing feature is that A games do not allow any seed to be taken out of the games; the captured seeds are sown in own front row. Thus, the number of seeds is constant. This makes these games divergent, complex and therefore difficult to solve using a computer. *Aware* in contrast is a converging, perfect information game, with fixed termination because the captured seeds are taken out of the game [59]. Coincidentally, the capture in type A games is synonymous with business transactions in which what is captured remains with the new owner but can be re-invested to capture more. This may lead to either more wins or losses of the acquired wealth.

**Table 1:** Mancala game type complexity classifications.

Game type	Distinguishing feature	Comments	Number of societies (total = 102)
**4A**	Games where captured seeds are fed into the player’s own holes, i.e. the number of seeds in play remains constant	Most complex and of huge variety	48
**4B**	Games where captured seeds are put aside out of play, i.e. the number of seeds in play gradually diminishes		16
**4C**	Combines Type-A capture rules with Type-B disposal of winnings (i.e. setting them aside out of play)		0
**4D**	Type B capture rules, but winnings are sown in Type A fashion on the player’s side of the board starting at the hole forward of that which received the previous last seed		1
**2A**	The capture of holes and subsequent use of these for the profit of the new owner and/or detriment of the original owner	This is the most complex and varied mode of capture in 2 row games.	13
**2B**	The capture of a given number of seeds with/following/opposite the last seed in hand and/or with the intermediate seeds of a sowing		8
**2C**	The accumulation of seeds in a predetermined hole		3
**2D**	The capture of seeds from opposite one of the player’s holes		11
**2E**	The capture of seeds from beyond an empty hole following that in which the last seed in hand was played		0

Source: Author compilation based on information from Townshend [55].

#### Spatial distribution of strategic games in culture

**Fig 2:**
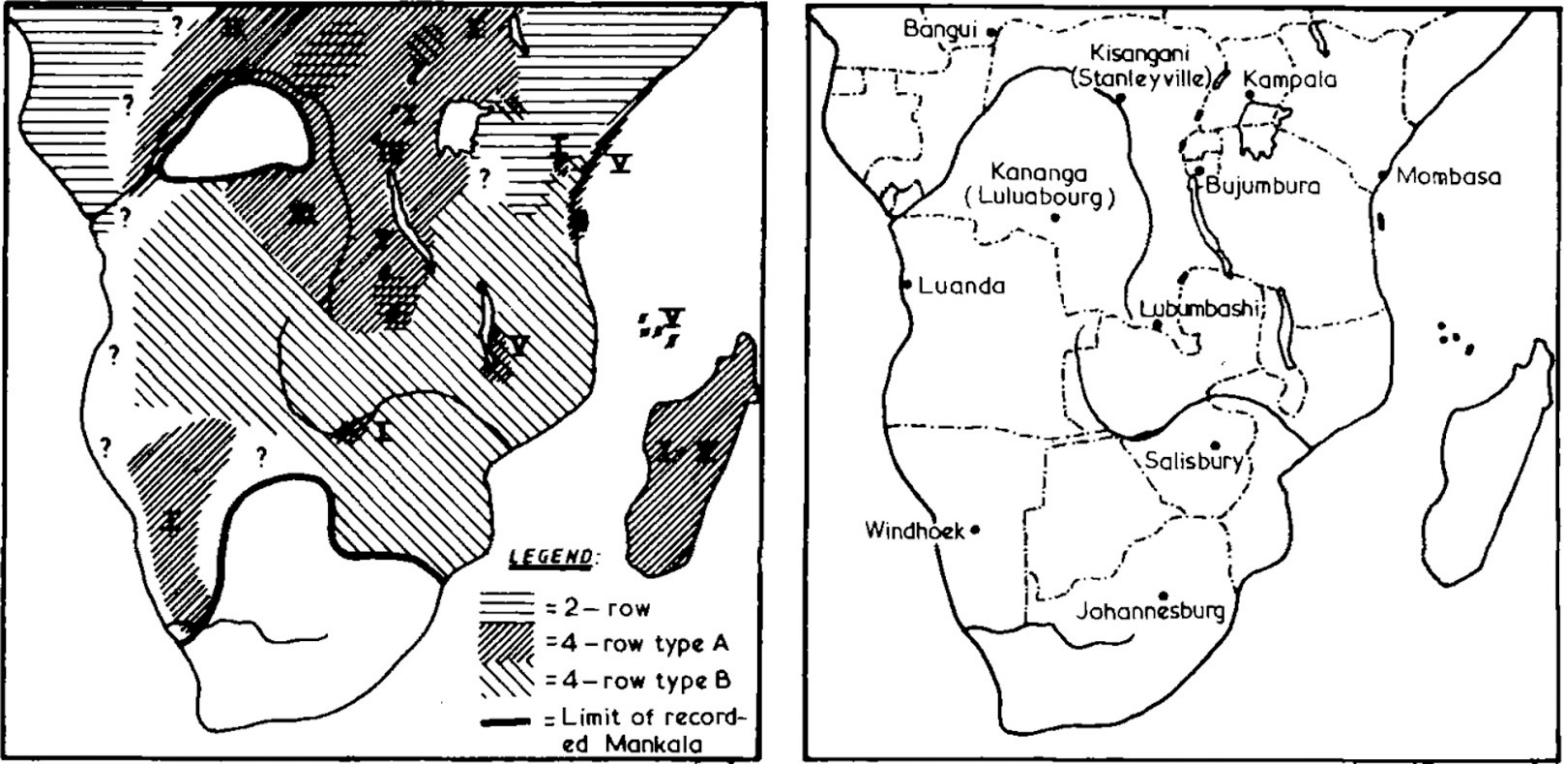
Approximate spatial distribution of main types of mancala in the twentieth century. Source: Townshend [55].

The advantage of the mancala data is that in many societies two or more modes of play co-exist, leaving a mosaic that can only be interpreted by a cross-cultural approach [55]. The variations in the game play can be attributed to many factors including migration, slavery, trade, and space/place [12, 64]. Fig 2 shows the approximate distribution of main types of mancala in the Central and Southern Africa. Murray [57] describes mancala games in parts of West Africa and North Africa as well. From these two sources, it is possible to match most of the societies in the ancestral characteristics data to their associated board games.

**Fig 3:**
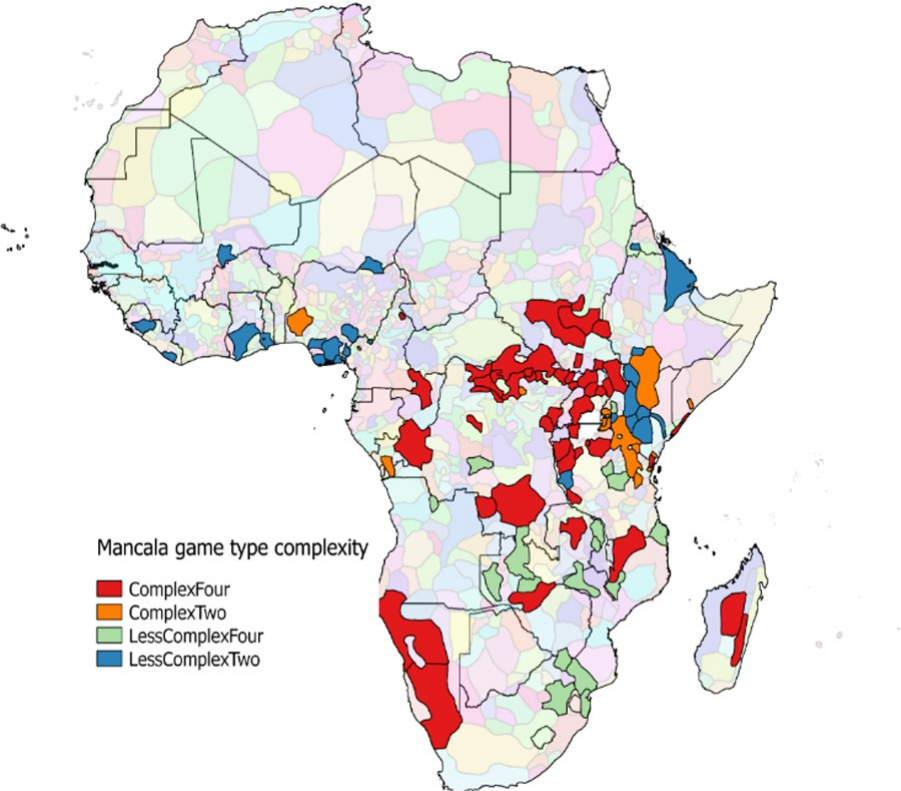
Mancala game type complexity. The blurred ethnic groups (data sourced from Giuliano and Nunn [53]) are not in the matched sample.

Fig 3 shows the diversity of ethnic groups in Africa and their geographic distribution across the continent. It is important to note that the size of the polygons is varied. There are about 531 ethnic groups in the ancestral characteristics database [53]. I matched these to the 325 ethnic group-mancala game combinations data getting about 182 matches with 102 unique ethnic groups. This preliminary matching was based only actual names of the ethnic groups. A more complex matching algorithm would increase this sample especially because the same ethnic groups are recorded using different names in the different sources. For example, the Chewa ethnic group in parts of Zambia and Malawi is denoted as Nyanja in the ancestral characteristics data.

The spatial pattern in the distribution of mancala game type complexity for the games played by each of the societies is evident in Fig 3. A spatial pattern is evident with most west African societies playing the two row games. South-eastern Africa plays four row games with a mix of game types in the central part of the continent. The maps in Figs 2 and 3 are consistent with separate accounts of the approximate geographical locations of 4A games (including *Omweso*, *Bao*, *Bosh*, *Nsumbi*, *Igisoro*, *Tsoro*, *Njombwa*, *Ryakati*, and *Bare*) by Wernham [63 p. 6].

#### Comparing compiled data to Murdock classification

The ancestral characteristics database has a variable on the presence and absence of different types of games. The expectation is that most of the ethnic groups in the mancala database will be classified as having games of strategy. Of the 102 matched ethnic groups, Murdock [65] game classification has only 19 non-missing observations (or ethnic groups). Seventeen of these indeed have games of strategy as expected (11 with skill and strategy and 6 with all games), one has games of physical skill only while one doesn’t have any of the games. Though with a small sample, Murdock’s classification is consistent with the mancala database. Besides the key sources of mancala games mentioned, there are other compilations of mancala games including a mancala website (the website provides about 140 mancala games with detailed descriptions of how each of the game is played, where and who plays it. The website is here: https://mancala.fandom.com/wiki/Mancala), not used in this paper because they do not contain information on the game complexity categories.

### Contemporary household survey data

Given that the mancala games database has a tribe identifier, one can link with other data with a tribe identifier. These may include national household surveys, Demographic and Health Surveys, Afrobarometer Surveys and population censuses. To present the usefulness of the mancala data, I use the afro barometer survey as provided by Nunn and Watchekon [54] to test the origins of entrepreneurship hypothesis.

## Empirical strategy

### Baseline estimating equations

I test *the games in culture* hypothesis (hypothesis 1) by estimating the following equation:

yk=α+βMancalaComplexityk+Xk'η+ϵk (1)

where *y*_*k*_ is an outcome of interest (e.g., political and economic complexity as defined in the ethnographic data) for each society *k*. *Mancala complexit y*_*k*_ is a vector of measures of game complexity that will be introduced in several specifications of the baseline regression. *X*_*k*_ is a vector of controls including ancestral dependence on agriculture, and slave trade. The sources of data for the controls are in S1 Table.

Similarly, I test *the origins of entrepreneurship* hypothesis (hypothesis 2) by estimating the following equation:

$$y_{ik}=\alpha +\beta \;Mancala\;Complexity_{ik}+X_{ik}^{'}\eta +\epsilon_{ik}$$ (2)

where *y*_*ik*_ is an outcome of interest (e.g., whether the individual is self-employed); *k*. *Mancala complexit y*_*ik*_ is a vector of measures of game complexity of the game the ethnic group the individual belongs played historically. *X*_*ik*_ is a vector of controls including country fixed effects, religion, distance to coast, slave trade, dependence on agriculture, distance to sea, initial population density, age, and gender. The controls were selected based on the prior literature that predicts entrepreneurial success [24]. Standard errors are clustered at ethnic group level.

Even after controlling for as many observed variables in variants of Eqs (1) and (2), it is likely the case that there are still omitted variables especially since the ancestral characteristics in the data were largely in the eyes of the observers, the anthropologists, not necessarily everything that constituted the persona of the people. The related literature in economics has often relied on spatial regression discontinuity designs [4, 66] and differential characteristics of immigrants aka epidemiological approach [67]. The spatial regression strategy involves identifying ethnic groups with different characteristics that share borders. This strategy controls for unobserved factors that vary smoothly across space but not those that vary discontinuously. We can’t use this research design because of data limitations especially on the location of individuals in the Afrobarometer survey and the small number of tribal shared borders in the sample. In addition, there is a considerable number of ethnic groups that play multiple mancala games. I use a two-pronged strategy to address identification concerns albeit not enough to make any causal statements at this stage of the research. First, I use Oster [68] sensitivity approach to determine whether unobservables are proportionally important to explain away the result. This is determined by a measure of the relative degree of selection of unobservables to selection of observables (*δ*). Second, to disentangle the effect of mancala game type complexity as a cultural trait from the geographical and historical environment in which the ethnic groups settled, I use the epidemiological approach which I discuss next in detail.

### Epidemiological approach

The epidemiological approach seeks to isolate the effect of the cultural phenomenon from the environment by comparing outcomes of migrants in a common economic environment [2, 67].This approach has been extensively used in analyzing the effects of culture on economic outcomes including female labor force participation and fertility [69], self-employment [70], and saving behavior [71].

Using the information of the ethnic group name based on the Murdock map as provided by Nunn and Watchekon [54], I selected individuals who are not residing in their ethnic homelands. These can be considered as immigrants in their current districts of residence. For these immigrants I estimated

$$y_{ik}=\alpha +\beta \;Mancala\;Complexity_{ik}+X_{ik}^{'}\eta +\epsilon_{ik}$$ (3)

Where *X* includes an interaction term for the fraction of ethnicity in a district and mancala game complexity of the ethnicity the person belongs to. The hypothesis here is that those from ethnic groups that traditionally played complex mancala and now reside in districts with other majority ethnicity are more likely to go into self-employment in non-farm sector because they do not have access to adequate land to go into farming. Playing complex mancala games gives them the comparative advantage at non-farm self-employment as compared to their counterparts from less complex mancala playing societies.

## Results and discussion

### Game type complexity and society socio-economic complexity

**Fig 4:**
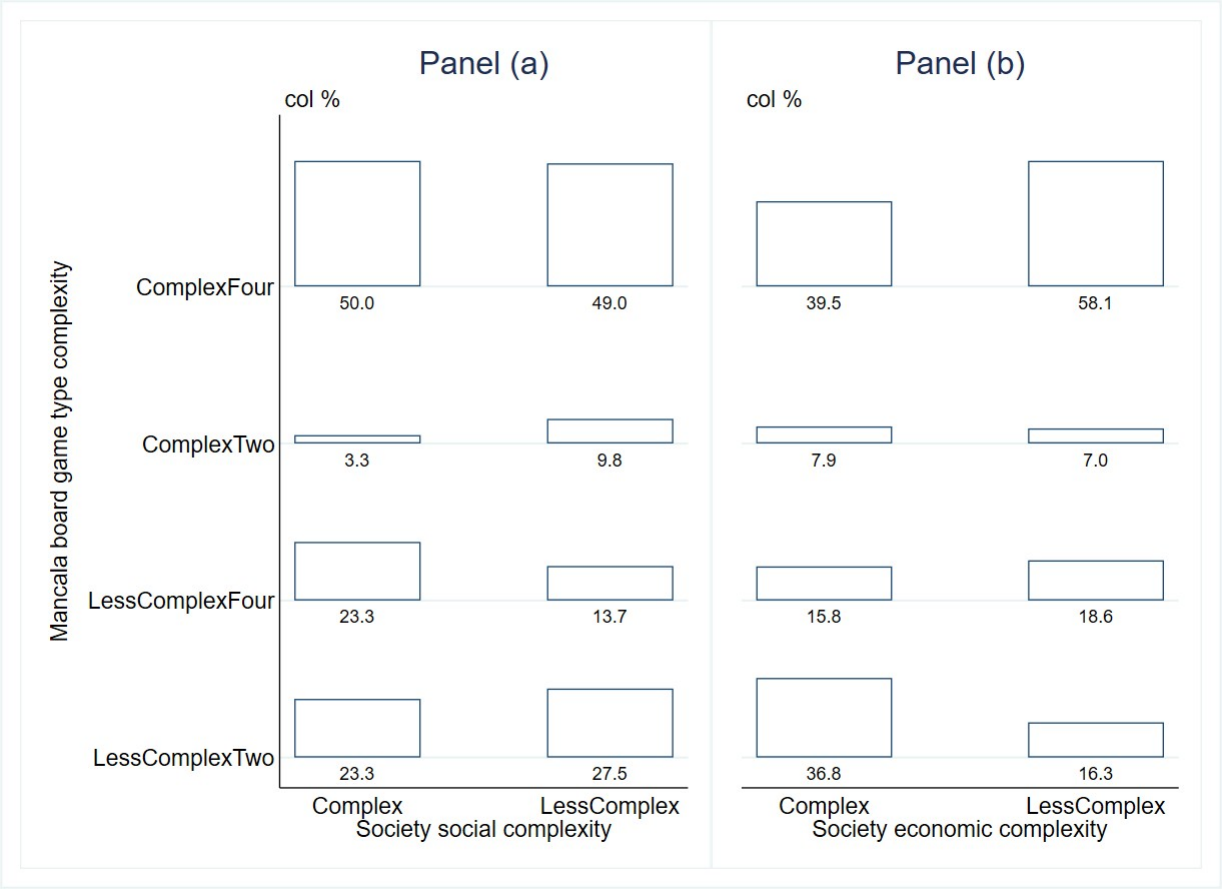
Table plot of mancala game type complexity and socio-economic complexity.

Fig 4 shows the percentage distribution of societies by socio-economic complexity (jurisdictional hierarchy beyond local community and settlement patterns) across the mancala game type complexity. Panel (a) of Fig 4 shows the classification by society social complexity (jurisdictional hierarchy beyond local community). About 37% of the ethnic groups had more than two levels of political jurisdiction beyond the local community, i.e., socially complex. These groups played both complex and less complex games almost equally (53.3% vs. 46.6%). For ethnic groups with none or one level of jurisdiction beyond local community (almost 63% of the societies), as much as 59% played complex mancala games.

In contrast to the game in culture hypothesis, these results show that societies that play complex two row or four row mancala games are even more likely to be those with less levels of jurisdiction beyond the local community (i.e., less socially complex societies). This essentially disproves the claims in the games in culture hypothesis.

Panel (b) of Fig 4 illustrates the relationship between game type complexity and complexity of settlement patterns (or society economic complexity). About 47% of the ethnic groups had complex settlement patterns. Almost 53% of these play the less complex games. For less complex two row games, it is the case that most societies that play the game are economically complex, contrary to the games in culture hypothesis. Overall, it appears that for both social and economic complexity, ethnic groups that played complex games are not any more complex on these indicators as espoused by the games in culture hypothesis [13].

**Fig 5:**
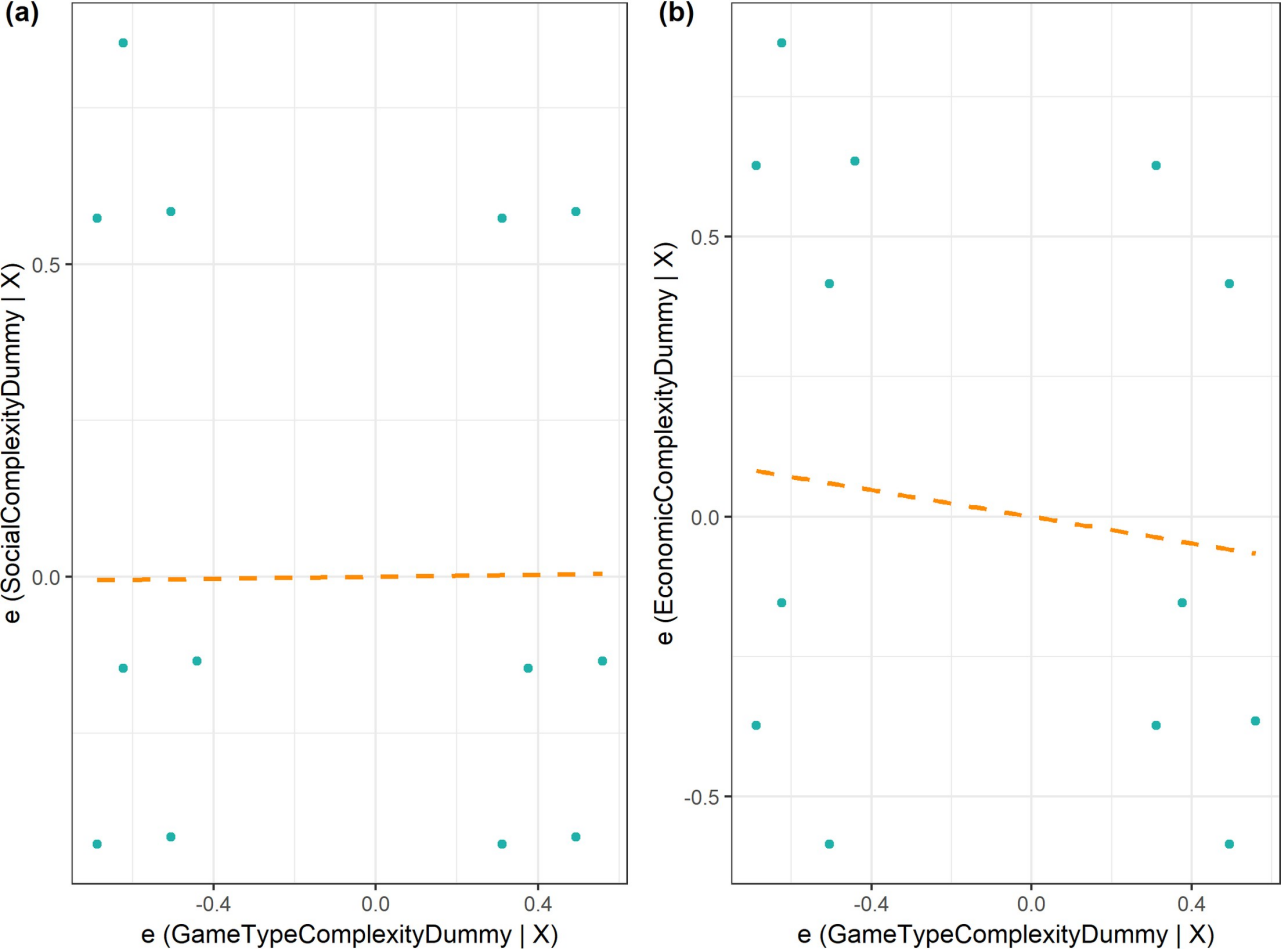
Partial correlation plots of game type complexity and socio-economic complexity. Controls include presence of slave trade and agriculture dependence.

The foregoing descriptive analysis disproves the hypothesis using cross tabulations as in the ensuing literature that supported it, but the analysis is simplistic because there are other confounding factors that may affect a society’s social and economic complexity. These include presence of slave trade and dependence on agriculture. We control for these factors in a regression of game type complexity on socio-economic complexity. Consistent with the descriptive analysis, Fig 5 shows no support for the games of strategy in culture hypothesis in that there is no evidence that societies playing complex game types are different from those not playing complex games in terms of the social and economic complexity (see S2 Table for the estimates). For each of the controlled specifications, the proportional selection of unobservables to observables is less than 1 implying that null result holds with addition of controls.

How do these null results relate to the findings and debates on games in culture hypothesis? First, note that all the studies on this hypothesis as reported in the Human Relations Area Files (https://hraf.yale.edu/ehc/hypotheses/1365) use the extensive margin—presence or absence of games of strategy—unlike in this paper. The rationale is however the same. Given that mancala games (all of which are games of strategy) are widespread across Africa, it is questionable to assume pre-colonial absence of these games for any ethnic group. Related to this difference, all the previous studies used small samples across societies in Asia, Africa and the rest of the world while in this paper I consider African games only.

### Results on origins of entrepreneurship hypothesis

**Fig 6:**
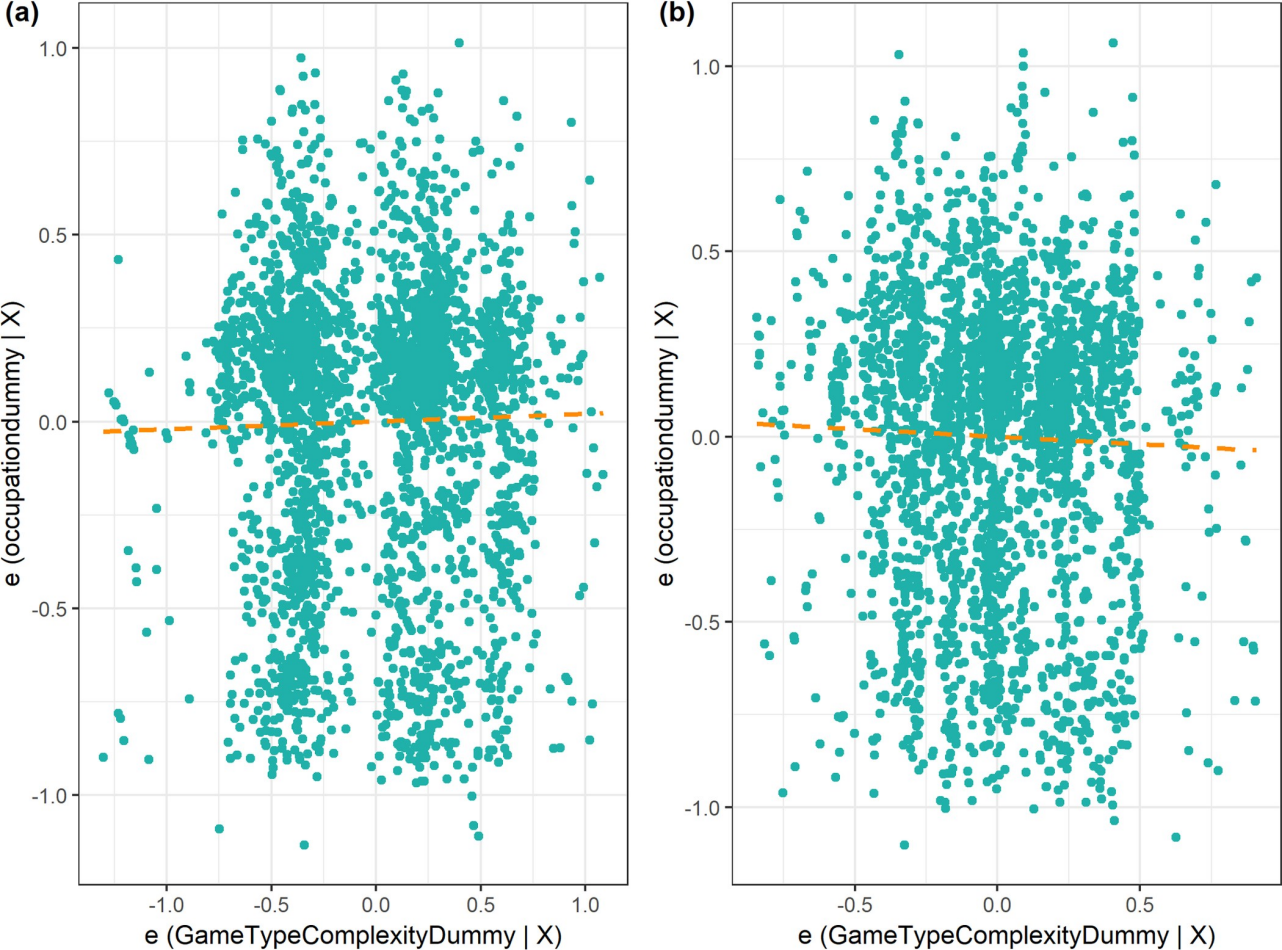
Partial correlation plots of game type complexity and contemporary occupation.

I present next results on the contemporary hypothesis that I have introduced—the origins of entrepreneurship hypothesis. The analysis is based on contemporary data at individual level unlike the results in the previous section which were from historical data at ethnic group level. Fig 6 shows using OLS estimates the relationship between the incidence of non-farm self-employment/ business ownership today and the game type complexity of the game that was played by the ethnic group from which the individual identifies with adjusting for a battery of controls. Panel (a) is for estimation without country fixed effects while panel (b) is for estimation with country fixed effects. There is a small sign flip on the game complexity estimate with addition of country fixed effects. S3 Table shows a test of the origins of entrepreneurship. It appears that the effect is null across most specifications. For each of the controlled specifications, the proportional selection of unobservables to observables is less than 1 implying that null result holds with addition of controls.

The literature on the relationship between pre-colonial institutions and economic outcomes in Africa (e.g., Michalopoulos and Papaioannou [18]) has consistently argued that OLS estimates of persistence of these features is problematic due to omitted variables. In the context of this paper, it is conceivable that several socioeconomic, geographical, historical and institutional factors that affect incidence of entrepreneurship are not included in the list of controls. The commonly used approach to deal with this endogeneity is to use a spatial regression discontinuity design, that is, using observations close to the ethnic boundary (e.g., Lowes [66]). This was not possible with the available data. Another approach which arguably does not address identification concerns fully but can plausibly help in disentangling effects of culture (e.g., playing of particular mancala games) from the historical and geographical factors is to restrict the analysis to individuals who are not living in their ethnic group homelands. These individuals face common conditions in the new homelands.

### Results using the epidemiological approach

To isolate the mancala complexity as a cultural trait of the ethnic groups for the individuals from the geography and institutions of the places they are living, I use the epidemiological approach. In most applications, the persistence of ancestral culture among those living elsewhere is considered a more conservative approach to establishing the effect of cultural traits on economic outcomes. Table 2 shows OLS estimates of the effect of game complexity on incidence of being self-employed in agricultural or non-agricultural sectors for those not living in their ancestral homelands. The results are similar to the full sample, with a null effect across all specifications for the main effect.

**Table 2:** OLS estimates of the effect of game complexity on entrepreneurship for immigrants.

	(1)	(2)	(3)	(4)	(5)
	Occupation (agriculture = 1)	Occupation (agriculture = 1)	Occupation (agriculture = 1)	Occupation (agriculture = 1)	Occupation (agriculture = 1)
**Game complexity**	0.0287	-0.0450	-0.0566	0.0426	0.0359
	(0.0718)	(0.0532)	(0.0371)	(0.0467)	(0.0445)
**Islam**		-0.0804	-0.0505	-0.0441	-0.0782
		(0.0409)	(0.0269)	(0.0259)	(0.0752)
**Fraction of ethnicity in district**		0.0748	0.108**	0.210***	0.208***
		(0.0419)	(0.0362)	(0.0535)	(0.0523)
**Game complexity x Fraction of ethnicity in district**				-0.187**	-0.184**
				(0.0615)	(0.0604)
**Game complexity x Islam**					0.0457
					(0.0801)
**Individual controls**	No	Yes	Yes	Yes	Yes
**Ethnicity controls**	No	Yes	Yes	Yes	Yes
**Country fixed effects**	No	No	Yes	Yes	Yes
*N*	2011	1585	1585	1585	1585
*R*^2^	0.001	0.349	0.387	0.391	0.391
adj. *R*^2^	0.000	0.340	0.374	0.378	0.378

*Notes*: The table reports OLS estimates. Robust (clustered at ethnic group level) standard errors in parentheses. An observation is an individual living in a district other than the ethnic homeland. Individual controls include education, age, age squared, urban residence, and gender. Ethnicity controls include distance to sea, initial population density, presence of slave trade and ancestor’s dependence on agriculture.

* *p* < 0.05,

** *p* < 0.01,

*** *p* < 0.001.

It is still natural to ask whether particular groups may benefit more from the skills their ancestors learnt in complex mancala. This can be through two main mechanisms commonly referred to as vertical transmission and horizontal transmission of culture. In the context of vertical transmission, the question is whether an individual from particular ethnic groups inherited the playing of complex mancala from direct parents or grand-parents. For horizontal transmission, the concern is whether the playing of mancala is salient when the individuals have peers in the foreign location to practice the skills with. In this paper, I focus on the horizontal transmission mechanism due to data limitations. Columns (4) and (5) show the negative interaction effect of game complexity and fraction of ethnicity in the district. As compared to individuals from ancestors who played less-complex games, those from ancestors that played complex mancala, likelihood of being self-employed in the non-farm sector increases especially with the increase in the fraction of ethnic compatriots in the foreign location. This can be christened as ‘peer effect’ of playing complex mancala games. It implies that complex mancala games teach some values important for creating business social networks. The ‘peer effect’ interpretation follows from Marcen [70] who in investigating the effects of culture on self-employment explored *‘whether second-generation immigrants responsiveness to parents’ country of origin self-employment rates vary*, *depending on whether they live in ethnic enclaves or communities with a majority of people of the same ancestry*.*’*

The ‘peer effect’ needs to be tested comprehensively in the context of this paper, but I will try to demonstrate using examples from the literature how this possibly works. Be warned, this is all speculative. First, the distinguishing feature of complex mancala games (Type 2A and Type 4A) is that captured seeds are fed into own holes rather than set aside as in other types of mancala. According to Iliffe [17], this type A feature (not universal as he thought) makes these ‘games of a society dedicated to building up its numbers’. Second, the little evidence of internal borders—ethnic-based market segmentation and favoritism—across ethnic lines [72] are much reported for business merchants from a complex mancala playing group e.g., the *Yaos*, in Malawi. Since immigrants are self-selected, we can assume that the most ambitious members of an ethnic group leave their homelands. The skills from a particular game play is thus activated among this group and when such immigrants reach some critical mass, business activities thrive among them. It may also have to do with whether these games are differentially played communally or within families. This logic is also suggested in a social networks model by Kerr and Mandorff [41] in which non-work relationships facilitate the acquisition of sector-specific skills that results in occupational stratification.

It is along these lines that Chachage [73] argues for the entrepreneurial success of East African Asians, *‘it is entrepreneurship networking*, *and not an innate entrepreneurial spirit*, *that explains the reproduction of East African Asian entrepreneurs and their relative business success’*. Lundy, Patterson and O’Neill [74] also documented the importance of social networks to the foreigner’s entrepreneurial success in Guinea-Bissau. Perhaps what is missing in this economic history and anthropology literature is the understanding of the circumstances that create such entrepreneurial networks. While still incomplete, the ‘peer effect’ finding in this paper can help in understanding the business management skills across different cultures and how a social network of business minded individuals can spur entrepreneurship in addition to the traditional entrepreneurial skills.

## Limitations

This paper is exploratory in nature and deals with hypotheses that are not well known except to board game researchers. Most of the evidence available is at best anecdotal. As a research pursuit, it therefore raises several questions regarding the appropriateness of the hypotheses, data quality and availability, and credibility of the empirical approach. The concerns espoused in these questions may also partly explain the null results presented in this paper.

On the appropriateness of the hypotheses, a natural question that arises on the relationship between entrepreneurship tendencies of contemporary societies and the playing of mancala games is whether the members today still play the same games their ancestors played. This is a difficult task and would require a comprehensive survey of games that are currently played in different societies across Africa. In this paper, I provide examples of anthropological and game ethnology studies that discuss the games currently played by members of societies in the database. Mancala is considered one of the most persistent aspects of African culture. The rules remain unchanged over centuries even though the stories in gameplay change. For instance, the rules of *Bao* documented in the 19^th^ century are the same as the rules used currently in Malawi and Tanzania. Bayeck [12] also discusses how *Songo* game remains important to the economic lives of Cameroonians and it is not only an object of art to preserve culture but can be of significance to research on literacy and learning, and to the design of environments that support learning.

It appears the persistence of mancala games as a cultural practice posits opportunities to understand the historical patterns in trade and migration [64]. Indeed, the similarity in the rules among some games may suggest social contact among the different societies [75]. That perhaps explains why ethnic groups that were in the Islamic trade route (e.g., *Yao* and *Swahili*) use similar game rules and terminology.

The second concern on the appropriateness of the hypotheses is that not all members of the ethnic group played or know how to play the game. Mancala games are complex and difficult to learn such that not everyone within an ethnic group plays the game. Without the knowledge of the proportion of people within an ethnic group whose ancestors played the game or current members who play the game, it is difficult to defend the empirical results presented.

On mancala games data quality and availability, there are several challenges that may affect the evidence presented in the paper. First, there is missingness of board games data for most of ethnic groups. Second, the descriptions and sources are from different time periods which may affect the comparability assuming that for some of the games the rules may have changed and that some games may have been transferred across ethnic groups without game anthropologists observing such transfers.

Related to the concerns on data quality and availability are concerns on the credibility of the empirical approaches. There are possibly many other unobserved factors that vary with the types of games played. Given the data constraints and the lack of knowledge regarding the location of invention of each of the board games, the paper is not making a causal claim. The merit of the study should be considered in providing a comprehensive database of mancala board games in Africa that can be linked to other datasets to spur research on these games because regardless of the hypotheses they remain important for African societies. In addition, the paper explores areas of research that are replete with case studies and anecdotes and thus attempts to provide a basis for causal empirical research.

## Conclusions

This study investigated the relationship between the playing of indigenous strategic board games and socio-economic complexity—the games in culture hypothesis. The study also introduced a new hypothesis—origins of entrepreneurship hypothesis—based on anecdotal evidence that ethnic groups that play complex mancala games are business ethnic groups. To test these hypotheses, I combined ancestral characteristics data, Afrobarometer data and game ethnology data. The ancestral characteristics data was available for 531 African societies. For each of these societies, I collected information on the games they played in the pre-colonial era from the anthropological and ethnological literature to create the first comprehensive compilation of Mancala Games in Africa Database. I then matched these to Afrobarometer data for 18 countries. The database and all code are provided in the supplementary materials. It is also possible to match the mancala database to all available demographic and household survey (DHS) data for African countries that have occupation and ethnicity variables.

I do not find evidence to support either hypothesis. These null results echo the views of the sceptics of games in culture hypothesis (e.g., de Voogt [28] and de Voogt [30]) and several null effect studies of game (especially chess) effects on cognitive skills (see a review by Sala, Foley and Gobet [76]). It is therefore not very surprising that game play does not affect socio-economic complexity or willingness to engage in entrepreneurship. Nonetheless, I find peer effects of game play in that for migrants, an increase in their numbers increases the likelihood of being in business for those from 2A and 4A (assumed complex in the game ethnology literature) mancala playing societies as compared to other mancala-type playing societies. These claims are controversial but with better data as documented in this paper researchers can start investigating the economic importance of mancala games beyond these claims. This study analyzed a game feature at the ethnicity level which masks the enormous individual differences in game play within the ethnic group. For example, Mkondiwa [8] discussed for the case of *Bao* in Malawi that some opening, and end of game moves can be considered risky and may be used to elicit the risk preferences of the players. The characterization of the aesthetics of mancala boards and rules also point to potential for entrepreneurship training as pointed by Townshend [23] in describing the links between *Bao* and the business-oriented Swahili ethic. It is therefore possible that regardless of the complexity of the game; variations in moves and other features may be more important for entrepreneurial training than the entire game itself. Entrepreneurship training materials using financial literacy in *Bao* have been recently developed by Somba [77]. Perhaps future research should directly test using randomized control trials an entrepreneurship curriculum that embeds known business-related mancala like *Bao*. The research can be along the lines of the heuristic or rule of thumbs entrepreneurship trainings (e.g., Dexler, Fischer and Schoar [26], and Arraiz, Bhanot and Calero [27]) and psychology-based entrepreneurial mindset training [78].

## Supporting information

S1 Table Variable definitions. (DOCX)

S2 Table OLS estimates of the effect of mancala game complexity on socio-economic complexity. (DOCX)

S3 Table OLS estimates of the effect of mancala game complexity on incidence of entrepreneurship. (DOCX)

S1 File Replication package. This zip file contains the underlying datasets, R code and the STATA do-file used to replicate the results of the manuscript.
(ZIP)

## References

[1] Nunn N. Culture and the Historical Process. Economic History of Developing Regions. 2012; 27(S1):108–126. 10.1080/20780389.2012.664864

[2] Alesina A., Giuliano P. and Nunn N. On the Origins of Gender Roles: Women and the Plough. The Quarterly Journal of Economics 2013; 128 (2): 469–530. 10.1093/qje/qjt005

[3] Gershman B. Witchcraft Beliefs and Erosion of Social Capital: Evidence from Sub-Saharan Africa and Beyond. Journal of Development Economics 2016;120: 182–208. 10.1016/j.jdeveco.2015.11.005.

[4] Moscona, J., Nunn, N. and Robinson, J.A. Segmentary Lineage Organization and Conflict in Sub-Saharan Africa. 2020. Available from: https://scholar.harvard.edu/nunn/publications/social-structure-and-conflict-evidence-sub-saharan-africa.

[5] Chen K.M. The Effect of Language on Economic Behavior: Evidence from Savings Rates, Health Behaviors and Retirement Assets. American Economic Review 2013; 103 (2): 690–731. 10.1257/aer.103.2.690 29524925

[6] Lowes S. Matrilineal Kinship and Spousal Cooperation: Evidence from the Matrilineal Belt. Forthcoming 2018 Available from: https://scholar.harvard.edu/files/slowes/files/lowes_matrilineal.pdf.

[7] Xue, M.M. and Michalopoulos, S. Folklore. NBER Working Paper 25430. [Posted 2019 Jan]. 2019. Available from: https://www.nber.org/papers/w25430.pdf.

[8] Mkondiwa M. Games of Strategy in Culture and Economics Research. Journal of Economic Methodology 2020; 27(2): 146–163. 10.1080/1350178X.2019.1680858

[9] Simon H.A. A Behavioral Model of Rational Choice. Quarterly Journal of Economics 1955; 69 (1): 99–118. 10.2307/1884852

[10] Gobet F., de Voogt A.J. and Retschitzki J. Moves in Mind: The Psychology of Board Games. Hove and New York, Psychology Press; 2004.

[11] Bayeck R.Y. A Review of Five African Board Games: Is There Any Educational Potential? Cambridge Journal of Education 2018; 48(5): 533–552. 10.1080/0305764X.2017.1371671

[12] Bayeck, R.Y. “Exploring the African Songo Game and How Gameplay Enhances Multiple Literacies among Adult Players in Cameroun and the United States.” PhD Dissertation. Pennsylavania State University. 2019. Available from: https://etda.libraries.psu.edu/catalog/16327ryb105.

[13] Roberts J.M., Arth M.J. and Bush R.R. Games in Culture. American Anthropologist 1959; 64 (4): 597–605. 10.1017/CBO9781107415324.004

[14] Roberts J.M. and Sutton-Smith B. Child Training and Game Involvement. Ethnology 1962; 1(2): 166–185. 10.2307/3772873

[15] Roberts J.M., Sutton-Smith B. and Kendon A. Strategy in Games and Folk Tales. Journal of Social Psychology 1963; 61: 185–199. 10.1080/00224545.1963.9919478 14084800

[16] Peregrine P. N. Political Strategy and Cross-Cultural Variation in Games. Cross-Cultrual Research 2008; 42: 386–393. 10.1177/1069397108321895

[17] Illife, J. Africans: The history of a continent. 2nd edition. Cambridge University Press; 2007.

[18] Michalopoulos S. and Papaioannou E. Pre-colonial Ethnic Institutions and Contemporary African Development. Econometrica 2013; 81 (1): 113–152. 10.3982/ECTA9613 25089052 PMC4118452

[19] Bandyopadhyay S. and Green E. Precolonial Political Centralization and Contemporary Development in Uganda. Economic Development and Cultural Change 2016; 64(3): 471–508. 10.1086/685410.

[20] Archibong B. Historical origins of persistent inequality in Nigeria. Oxford Development Studies 2018; 46 (3): 325–347. 10.1080/13600818.2017.1416072

[21] Archibong B. Explaining divergence in the long-term effects of precolonial centralization on access to public infrastructure services in Nigeria. World Development 2019; 121: 123–140. 10.1016/j.worlddev.2019.04.014.

[22] Mwale S.K. Bao. The Society of Malawi Journal 1996; 49 (1): 56–70. Available from: https://www.jstor.org/stable/29778749.

[23] Townshend, P. Bao (Mankala): The Swahili Ethic in African Idiom. Paideuma, Bd.28, From Zinj to Zanzibar: Studies in History, Trade and Society on the Eastern Coast of Africa; 1982. Pp. 175–191. Available from: https://www.jstor.org/stable/41409882.

[24] McKenzie D. and Sansone D. Predicting Entrepreneural Success is Hard: Evidence from a Business Plan Competition in Nigeria. Journal of Development Economics 2019; 141:102369 10.1016/j.jdeveco.2019.07.002.

[25] Iyer R., & Shoar A. Are there cultural determinants of entrepreneurship? In: International Differences in Entrepreneurship, edited by Lerner J., & Schoar A. University of Chicago Press Chicago and London 2010 10.7208/chicago/9780226473109.003.0008

[26] Drexler A., Fischer G., & Schoar A. Keeping It Simple: Financial Literacy and Rules of Thumb. American Economic Journal: Applied Economics 2014; 6(2): 1–31. 10.1257/app.6.2.1.25485039

[27] Arraiz, I., Bhanot, S.P., & Calero, C. Less is More: Experimental Evidence on Heuristic-Based Business Training in Ecuador. Inter-American Development Cooperation. 2019. Working Paper. Available from: https://www.idbinvest.org/en/publications/report-less-more-experimental-evidence-heuristics-based-business-training-ecuador.

[28] de Voogt A.J. Strategic Games in Society: The Geography of Adult Play. International Journal of Play 2017; 6 (3): 308–318. 10.1080/21594937.2017.1382986

[29] Chick G. Comment on ‘Strategic Games in Society: The Geography of Adult Play’. International Journal of Play 2017; 6 (3): 319–321. 10.1080/21594937.2017.1382990.

[30] de Voogt A.J. The Absence of Games in the Presence of Claims. Reply to Garry Chick. International Journal of Play 2017; 6 (3): 322–323. 10.1080/21594937.2017.1382991

[31] Pankhurst R. Gabata and Other Board-Games of Ethiopia and the Horn of Africa. Azania: Archaelogical Research in Africa 1982; 17(1): 27–42. 10.1080/00672708209511298

[32] Ball D.W. The Scaling of Gaming: Skill, Strategy and Chance. The Pacific Sociological Review 1972; 15 (3): 277–294. 10.2307/1388347

[33] Silver B.B. Social Structure and Games: a Cross-Cultural Analysis of the Structural Correlates of Game Complexity. The Pacific Sociological Review 1978; 21 (1): 85–102. 10.2307/1388869

[34] Chick G. Games in culture revisited: a replication and extension of Roberts, Arth and Bush (1959). Cross-Cultural Research 1998; 32: 185–206. 10.1177/106939719803200204

[35] Weber, M. Die protestantische Ethik und der ‘Geist’ des Kapitalismus, Archiv fur Sozialwissenschaft und Sozialpolitik 20: 1–54 and 21: 1–110; reprinted in Gesammelte Aufs¨atze zur Religionssoziologie, pp. 17–206, 1920; English translation as The Protestant Ethic and the Spirit of Capitalism, translated by Talcott Parsons (London, UK: Routledge Classics, [1930] 2001). 1905.

[36] Becker S.O. and Woessman L. Was Weber Wrong? A Human Capital Theory of Protestant Economic History. The Quarterly Journal of Economics 2009; 124(2): 531–596. 10.1162/qjec.2009.124.2.531.

[37] Ochonu, M.E. Entrepreneurship in Africa: A historical approach. Indiana University Press; 2018.

[38] Jalloh, A. African Entrepreneurship: Muslim Fula Merchants in Sierra Leone. Ohio University Center for International Studies.1999.

[39] Erhardt, K. and Haenni, S. Born to be an Entrepreneur? How Cultural Origin Affects Entrepreneurship. KOF Working Papers, No. 446. 2018. Available from: https://www.research-collection.ethz.ch/handle/20.500.11850/310085.

[40] Botticini M. and Eckstein Z. Jewish Occupational Selection: Education, Restrictions, or Minorities. Journal of Economic History 2005; 65(4): 922–948. 10.1017/S0022050705000355.

[41] Kerr, W.R. and Mandorff, M. Social networks, ethnicity and entrepreneurship. NBER Working Paper No. 21597. [Posted September 2015, Revised July 2019]. 2019. Available from: https://www.nber.org/papers/w21597.

[42] Alkan W. Entrepreneurs and Entrepreneurship in Africa. The World Bank Research Observer 1988; 3(2):171–188. 10.1093/wbro/3.2.171

[43] Marris P. The Social Barriers to African Entrepreneurship. The Journal of Development Studies 2017; 5(1): 29–38. 10.1080/00220386808421279.

[44] Townshend P. African Mankala in Anthropological Perspective. Current Anthropology 1979; 20 (4): 794–96. 10.1086/202380

[45] Boyd R. and Richerson P.J. Culture and the Evolutionary Process. The University of Chicago Press Chicago 1985.

[46] Boyd R. and Richerson P.J. The Origin and Evolution of Cultures. Oxford University Press Oxford 2005.

[47] Costa-Gomes M.A. and Crawford V.P. “Cognition and Behavior in Two-Person Guessing Games: An Experiemental Study.” American Economic Review 2006; 95(5): 1737–1768. 10.1257/aer.96.5.1737

[48] Camerer C.F., Ho T. and Chong J. A Cognitive Hierarchy Model of Games. The Quarterly Journal of Economics 2004; 119 (3): 861–898. 10.1162/0033553041502225.

[49] Roberts J.M. and Forman M.L. Riddles: Expressive Models of Interrogation. Ethnology 1971; 10 (4): 509–533. 10.2307/3773178

[50] Kaphagawani D.N. and Chidammodzi H.P. Chewa Cultural Ideals and System of Thought as Determined from Proverbs: A Preliminary Analysis. Pula: Botswana Journal of African Studies 1983; 3: 29–37. Available from: http://pdfproc.lib.msu.edu/?file=/DMC/African%20Journals/pdfs/PULA/pula003002/pula003002004.pdf.

[51] Kayange G. “Understanding the semantics of Chichewa proverbs in the light of contemporary philosophy of language.” Journal of African Cultural Studies 2014; 26(2), 220–233. 10.1080/13696815.2014.887461

[52] Graff E. Some Proverbs of the Nyanja People. African Studies 1944; 3(3): 101–128. 10.1080/00020184408706647

[53] Giuliano P. and Nunn N. Ancestral Characteristics of Modern Populations. Economic History of Developing Regions 2018; 33 (1): 1–17. 10.1080/20780389.2018.1435267

[54] Nunn N. and Wantchekon L. The Slave Trade and the Origins of Mistrust in Africa. American Economic Review 2011; 101 (7): 3221–52. 10.1257/aer.101.7.3221

[55] Townshend P. Mankala in Eastern and Southern Africa: A Distributional Analysis. Azania: Archaeological Research in Africa 1979; 14 (1): 109–38. 10.1080/00672707909511266

[56] Sanderson M.G. Native Games of Central Africa. The Journal of the Royal Anthropological Institute of Great Britain and Ireland 1913; 43: 726–736.

[57] Murray H.J.R. A History of Board Games Other than Chess Oxford: Clarendon Press 1952.

[58] Walker, R.A. Sculptured mancala gameboards of sub-Saharan Africa. PhD Thesis, Indiana University, Indiana, USA.1990.

[59] Allis, L. V. Searching for solutions in games and artificial intelligence. PhD Thesis; 1994. Maastricht. Available from: http://fragrieu.free.fr/SearchingForSolutions.pdf.

[60] Divilly, C., O’Riordan, C. and Hill, S. Exploration and Analysis of the Evolution of Strategies for Mancala Variants. 2013. IEEE Conference on Computational Intelligence and Games. 10.1109/CIG.2013.6633628

[61] Donkers, J., Uiterwijk, J. and de Voogt, A. Mancala games-Topics in Mathematics and Artificial Intelligence. In: Step by Step. Proceedings of the 4th Colloquium ‘Board Games in Academia’ eds. Retschitzki, J. and Haddad-Zubel,R. Fribourg, Switzerland: Universsity of Fribourg. 2001. pp. 133–146.

[62] Donkers, J. and Uiterwijk, J. Programming Bao. In: Seventh Computer Olympiad: Computer-Games Workshop Proceedings: Technical Reports in Computer Science, CS 02–03. Edited by J. Uiterwijk, Maastricht, Nethelands. 2002.

[63] Wernham, B. Omweso, the Royal Mancala of Uganda: A General Overview of Current Research. In: Step by Step: Proceedings of the 4th Colloquium “Board Games in Academia,” edited by Jean Retschitzki and Rosita Haddad-Zubel. Fribourg, Switzerland.2002.

[64] de Voogt A.J. Distribution of Mancala Board Games: A Methodological Inquiry. Journal of Board Game Studies 1999; 2: 104–114. Available from: https://www.researchgate.net/publication/239522959.

[65] Murdock, G.P. Ethnographic Atlas. Pittsburgh: University of Pittsburgh Press. 1967.

[66] Lowes S., Nunn N., Robinson J.A. and Weigel J.L. The Evolution of Culture and Institutions: Evidence From the Kuba Kingdom. Econometrica 2017; 85 (4): 1065–91. 10.3982/ECTA14139

[67] Fernandez R. “Chapter 11- Does culture matter?” In Handbook of Social Economics Volume 1A: 481–510. 2011 10.1016/B978-0-444-53187-2.00011-5.

[68] Oster E. Unobservable Selection and Coefficient Stability: Theory and Evidence. Journal of Business and Economic Statistics 2019; 37 (2): 187–204. 10.1080/07350015.2016.1227711

[69] Fernandez R. and Fogli A. Culture: An Empirical Investigation of Beliefs, Work and Fertility. American Economic Journal: Macroeconomics 2009; 1(1): 146–177. 10.1257/mac.1.1.146

[70] Marcen M. The Role of Culture on Self-employment. Economic Modelling 2014; 44 Supplement 1: S20–S32. 10.1016/j.econmod.2013.12.008.

[71] Fuchs-Schundeln N., Masella P. and Paule-Paludkiewicz H. “Cultural Determinants of Household Saving Behavior.” Journal of Money, Credit and Banking.2019 Forthcoming. 10.1111/jmcb.12659

[72] Robinson A.L. Internal Borders: Ethnic-Based Market Segmentation in Malawi. World Development 2016; 87: 371–384. 10.1016/j.worlddev.2016.07.006

[73] Chachage, C. Globalization and the Making of East Africa’s Asian Entrepreneurship Networks. In: Entrepreneurship in Africa: A Historical Approach, edited by Ochonu, M.E. Indiana University Press; 2018. Part I.

[74] Lundy B.D., Patterson M. and O’Neill A. Drivers and Deterrents of Entrepreneurial Enterprise in the Risk-Prone Global South. Economic Anthropology 2017; 4: 65–81. 10.1002/sea2.12073

[75] Natsoulas A. The Game of Mancala with Reference to Commonalities among the Peoples of Ethiopia and in Comparison to Other African Peoples: Rules and Strategies. Northeast African Studies 1995; 2(2): 7–24. Available from: https://www.jstor.org/stable/41931202.

[76] Sala G., Foley J.P. and Gobet F. The Effects of Chess Instruction on Pupils’ Cognitive and Academic Skills: State of the Art and Theoretical Challenges. Frontiers in Psychology 2017; 8:1–48. 10.3389/fpsyg.2017.00001 28280476 PMC5322219

[77] Somba, A. Financial Literacy in Bawo (S1.E1). 2020. Available from: https://viseriq.com/05/29/financial-literacy-in-bawo-s1-e1/.

[78] Campos F., Frese M., Goldstein M., Iacovone L., Johnson H. C., McKenzie D., et al “Teaching Personal Iniative Beats Traditional Training in Boosting Small Business in West Africa.” Science 2017; 357 (6357): 1287–1290. 10.1126/science.aan5329 28935805

