# Supplementary material for: Mancala board games and origins of entrepreneurship in Africa
Source: PLoS One. 2020 Oct 15;15(10):e0240790. doi: 10.1371/journal.pone.0240790 (PMC7561206; doi:10.1371/journal.pone.0240790)
Supplement: S2 Table — (DOCX) [file pone.0240790.s002.docx]

S2 Table: OLS estimates of the effect of mancala game complexity on socio-economic complexity

|  | (1) | (2) | (3) | (4) |
| --- | --- | --- | --- | --- |
|  | Social Complexity | Social  Complexity | Economic Complexity | Economic Complexity |
| Game complexity | -0.0522 | 0.00806 | -0.180 | -0.118 |
|  | (0.110) | (0.117) | (0.112) | (0.114) |
| Controls | No | Yes | No | Yes |
| *N* | 81 | 76 | 81 | 76 |
| *R*^2^ | 0.003 | 0.050 | 0.032 | 0.107 |
| adj. *R*^2^ | -0.010 | 0.010 | 0.020 | 0.070 |

*Notes:* The table reports OLS estimates. Robust (clustered at ethnic group level) standard errors in parentheses. An observation is a tribe. Controls include presence of slave trade and ancestor’s dependence on agriculture.

^*^ *p* < 0.05, ^**^ *p* < 0.01, ^***^ *p* < 0.001.
