# Supplementary material for: Mancala board games and origins of entrepreneurship in Africa
Source: PLoS One. 2020 Oct 15;15(10):e0240790. doi: 10.1371/journal.pone.0240790 (PMC7561206; doi:10.1371/journal.pone.0240790)
Supplement: S3 Table — (DOCX) [file pone.0240790.s003.docx]

S3 Table: OLS estimates of the effect of game complexity on entrepreneurship (occupational choice)

|  | (1) | (2) | (3) | (4) | (5) |
| --- | --- | --- | --- | --- | --- |
|  | Occupation (agriculture=1) | Occupation (agriculture=1) | Occupation (agriculture=1) | Occupation (agriculture=1) | Occupation (agriculture=1) |
| **Game complexity** | **0.0548** | **0.0191** | **-0.0589^*^** | **0.00695** | **0.00976** |
|  | **(0.0596)** | **(0.0441)** | **(0.0263)** | **(0.0405)** | **(0.0398)** |
|  |  |  |  |  |  |
| Islam |  | -0.0709 | -0.0452^*^ | -0.0459^*^ | -0.0332 |
|  |  | (0.0361) | (0.0196) | (0.0198) | (0.0454) |
|  |  |  |  |  |  |
| Fraction of ethnicity in district |  | 0.0859^**^ | 0.137^***^ | 0.186^***^ | 0.187^***^ |
|  |  | (0.0317) | (0.0269) | (0.0362) | (0.0360) |
|  |  |  |  |  |  |
| Game complexity x Fraction of ethnicity in district |  |  |  | -0.0987 | -0.0997 |
|  |  |  |  | (0.0513) | (0.0514) |
|  |  |  |  |  |  |
| Game complexity x Islam |  |  |  |  | -0.0173 |
|  |  |  |  |  | (0.0466) |
| Individual controls | No | Yes | Yes | Yes | Yes |
| Ethnicity controls | No | Yes | Yes | Yes | Yes |
| Country fixed effects | No | No | Yes | Yes | Yes |
| *N* | 3576 | 2917 | 2917 | 2917 | 2917 |
| *R*^2^ | 0.003 | 0.307 | 0.349 | 0.350 | 0.350 |
| adj. *R*^2^ | 0.003 | 0.303 | 0.341 | 0.342 | 0.342 |

*Notes:* The table reports OLS estimates. Robust (clustered at ethnic group level) standard errors in parentheses. An observation is an individual. Individual controls include education, age, age squared, urban residence, and gender. Ethnicity controls include distance to sea, initial population density, presence of slave trade and ancestor’s dependence on agriculture. ^*^ *p* < 0.05, ^**^ *p* < 0.01, ^***^ *p* < 0.001.
