# Supplementary material for: Mancala board games and origins of entrepreneurship in Africa
Source: PLoS One. 2020 Oct 15;15(10):e0240790. doi: 10.1371/journal.pone.0240790 (PMC7561206; doi:10.1371/journal.pone.0240790)
Supplement: S1 File — This zip file contains the underlying datasets, R code and the STATA do-file used to replicate the results of the manuscript. (ZIP) [file pone.0240790.s004.zip › replicationfiles/Appendices.docx]

# Appendices

Table A1: Variable definitions

| Variable name | Variable description | Construction from data source | Data source |
| --- | --- | --- | --- |
| ***Dependent variables*** |  |  |  |
| Economic complexity | 1= Complex  0= Not complex | Code categories (8= compact and relatively permanent, and 9= complex settlements) into complex while the other categories into not complex | Ancestral characteristics data variable v30 |
| Political complexity | 1=Complex  0=Not complex | Code categories (two levels to four levels) into complex while the other categories into not complex | Ancestral characteristics data variable v33 |
| Entrepreneurship participation | 1=if individual (aged 25 or older) is engaged in non-farm self-employment  0= otherwise | Following data from Nunn and Watchekon (2011)  ***0= On-farm self-employment*** (Q95: 1=Subsistence Farmer (produces only for home consumption), 2=Peasant Farmer (produces both for own consumption and some surplus produce for sale), 3=Commercial Farmer (produces mainly for sale), 4=Farm worker, 5=Fisherman, 25=Pastoralist/herder/raise livestock);  ***1= Non-farm self-employment/ owning a business*** (Q95: 6=Trader/Hawker/Vendor, 15= Businessperson (works in the company of others), 16= Businessperson (Owns small business of less than 10 employees), 17=Businessperson (Owns large business of more than 10 employees)^[[1]](#footnote-1)^ | Afrobarometer data. Q95: What is your main occupation? |
|  | 1=Non-farm self employment/ owning a business  0=farm worker |  |  |
| ***Independent variables of interest*** |  |  |  |
| Game type (simple classification) | 2 row (A,B,C,D,E) dummies  4 row (A^[[2]](#footnote-2)^, B) dummies | As already coded by Murray (1952) and Townshend (1979b) | Townshend (1979b) and Murray (1952) |
| Game type (detailed classification) | Multiple variables: Number of rows, number of columns, number of stones used, directions of play allowed, number of special pockets, number of stones per pocket | As reported in the source | Townshend (1979b) and Murray (1952) |
| Mancala complexity | Game tree complexity | Author computations using formulae in Allis (1994) | Townshend (1979b) and Murray (1952) |
| ***Controls*** |  |  |  |
| Country | Country dummies | Matching using geocoordinates | Ancestral characteristics data and Afrobarometer |
| Distance to coast | Distance to nearest coast in kilometers | Using ancestral characteristics geo-coordinates (v104, v106), Afrobarometer geocoordinates and Africa’s coastline shapefile. | Ancestral characteristics data and Afrobarometer survey |
| Slave trade | 1=Slave trade, 0= No slave trade | Code categories (2=absence or near absence) as no slave trade and the other categories as slave trade. | Ancestral characteristics data variable v70 |
| Ruggedness | Ruggedness index | Using a procedure reported Nunn and Puga (2012) | Ancestral characteristics data, Nunn and Puga (2012) |
| Period observed | 1=Pre-colonial  0= Colonial | Years before 1890 as pre-colonial and years after as colonial | Ancestral characteristics data variable v102 |
| Agricultural suitability index | Pixel level crop suitability | As in the data | FAO Global Agro Ecological Zones (GAEZ) |
| Religion | Islam dummy  Christianity dummy | As in the data | Afrobarometer |
|  | 1=Islam  0=Other | 1=Islam (11=Muslim, Sunni, 12=Muslim, Shiite, 15= Muslim (general/other))  0=Other (includes all the codes except the ones for Islam) | Afrobarometer survey |
| Demographic variables | Age, age squared, gender, marital status, population density, education, urban/rural | As in the data | Afrobarometer survey |
| Agricultural dependence | 0=Less dependent  1=More dependent | 0= Less dependent on agriculture (1=0-5% dependence, 2=6-15% dependence, 3=16-25% dependence, 4=26-35% dependence, 5=36-45% dependence, 6=46-55% dependence),  1= More dependent on agriculture (7=56-65% dependence | Ancestral characteristics data variable v5 |
|  |  |  |  |
| ***Variables for matching across data sources*** |  |  |  |
| Geo-variables | Latitude and longitude |  | Ancestral characteristics data (v104, v106) |
| Ethnicity | Society name | Use Ancestral characteristics society names as benchmark for matching | Ancestral characteristics data (v107: society name), Afrobarometer survey, mancala data |

1. 1=Subsistence Farmer (produces only for home consumption), 2=Peasant Farmer
   (produces both for own consumption and some surplus produce for sale), 3=Commercial Farmer (produces mainly
   for sale), 4=Farm worker, 5=Fisherman, 6=Trader/Hawker/Vendor, 7=Miner, 8=Domestic
   Worker/Maid/Char/Househelp, 9=Armed Services/Police/Security Personnel, 10=Artisan/skilled manual worker -
   formal sector, 11= Artisan/skilled manual worker - informal sector, 12=Clerical Worker, 13= Unskilled manual in
   the formal sector, 14= Unskilled manual worker in the informal sector, 15= Businessperson (works in the company
   of others), 16= Businessperson (Owns small business of less than 10 employees), 17=Businessperson (Owns large
   business of more than 10 employees), 18= Professional Worker (e.g., lawyer, accountant, nurse, engineer, etc.), 19=
   Supervisor/Foreman, 20=Teacher, 21=Government Worker, 22=Retail worker, 23= Student, 24=Housewife/Works
   In the Household, 25=Pastoralist/herder/raise livestock. [↑](#footnote-ref-1)
2. 4 row A games are the most complex. [↑](#footnote-ref-2)
