# Supplementary material for: Mancala board games and origins of entrepreneurship in Africa
Source: PLoS One. 2020 Oct 15;15(10):e0240790. doi: 10.1371/journal.pone.0240790 (PMC7561206; doi:10.1371/journal.pone.0240790)
Supplement: S1 File — This zip file contains the underlying datasets, R code and the STATA do-file used to replicate the results of the manuscript. (ZIP) [file pone.0240790.s004.zip › replicationfiles/dta/merged_r3_codebook2_0.pdf]

Data Codebook

for

Round 3 Afrobarometer Surveys

in

18 African Countries

(Benin, Botswana, Cape Verde, Ghana, Kenya, Lesotho, Madagascar, Malawi, Mali, Mozambique, Namibia, Nigeria, Senegal, South Africa, Tanzania, Uganda, Zambia, Zimbabwe)

Prepared by:
Danielle Carter
Michigan State University
January 2008

The Institute for Democracy in South Africa (IDASA)
6 Spin Street, Church Square
Cape Town 8001, South Africa
27 21 461 2559•fax: 27 21 461 2589
Mattes (bob@idasact.org.za)

Ghana Centre for Democratic Development (CDD-Ghana)
14 West Airport Residential Area
P.O. Box 404, Legon-Accra, Ghana
233 21 776 142•fax: 233 21 763 028
Gyimah-Boadi (cdd@ghana.com)

Michigan State University (MSU)
Department of Political Science
East Lansing, Michigan 48824
517 353 3377•fax: 517 432 1091
Bratton (mbratton@msu.edu)

Copyright Afrobarometer

Question Number: country
Question: Country
Variable Label: Country
Values: 1-18
Value Labels: 1=Benin, 2=Botswana, 3=Cape Verde, 4=Ghana, 5=Kenya, 6=Lesotho, 7=Madagascar, 8=Malawi, 9=Mali, 10=Mozambique, 11=Namibia, 12=Nigeria, 13=Senegal, 14=South Africa, 15=Tanzania, 16=Uganda, 17=Zambia, 18=Zimbabwe
Note:

Question Number: respno
Question: Respondent number
Variable Label: Respondent number
Values: BEN0001-BEN1200, BOT0001-BOT1200, CVE0001-1261, GHA0001-GHA1200, KEN0001-KEN1278, LES0001-LES1161, MAD0001-1350, MWI0001 – MWI1200, MLI0001-1244, MOZ0001-MOZ1623, NAM0001-NAM1200, NIG0001-NIG2630, SEN0001-SEN1200, SAF0001-SAF2618, TAN0001-TAN1304, UGA0001-UGA2400, ZAM0001-ZAM1205, ZIM0001-ZIM1112
Value Labels: String variable
Note: Assigned by data managers

Question Number: backchk
Question: Household back-checked?
Variable Label: Household back-checked?
Values: 1-2
Value Labels: 1=Yes, 2=No
Note: Answered by interviewer

Question Number: urbrur
Question: PSU/EA
Variable Label: Urban or Rural Primary Sampling Unit
Values: 1-2
Value Labels: 1=urban, 2=rural
Note: Answered by interviewer

Question Number: region
Question: Region
Variable Label: Province or Region
Values: 100-108, 120-131, 140-151, 153-155, 160-164, 180-189, 200-207, 220-229, 240-245, 260-262, 280-288, 300-310, 320-332, 340-370, 380-402, 404, 420-428, 440-449, 540-545, 550-560
Value Labels: 100=Eastern Cape, 101=Free State, 102=Gauteng, 103=KwaZulu-Natal, 104=Limpopo, 105=Mpumalanga, 106=North West, 107=Northern Cape, 108=Western Cape, 120=Alibori, 121=Atacora, 122=Atlantique, 123=Borgou, 124=Collines, 125=Couffo, 126=Donga, 127=Littoral, 128=Mono, 129=Ouéme, 130=Plateau, 131=Zou, 140=Central, 141=Chobe, 142=Francis Town, 143=Gaborone, 144=Ghanzi, 145=Jwaneng, 146=Kgalagadi, 147=Kgatleng, 148=Kweneng, 149=Lobatse, 150=North East, 151=Ngamiland, 153=Selibe Phikwe, 154=South East, 155=Southern, 160=Santo Antao, 161= Sao Vicente, 162=Santiago-Interior, 163=Santiago-Praia, 164=Fogo, 180=Western, 181=Central, 182=Greater-Accra, 183=Volta, 184=Eastern, 185=Ashanti, 186=Brong Ahafo, 187=Northern, 188=Upper East, 189=Upper West, 200=Nairobi, 201=Central, 202=Eastern, 203=Rift Valley, 204=Nyanza, 205=Western, 206=North Eastern, 207=Coast, 220=Maseru, 221=Mafeteng, 222=Mohale’s Hoek, 223=Quthing, 224=Qacha’s Nek, 225=Mokhotlong, 226=Butha-Buthe, 227=Leribe, 228=Berea, 229=Thaba-Tseka, 240=Antananarivo, 241=Fianarantsoa, 242=Toamasina, 243=Mahajanga, 244=Toliary, 245=Antsiranana, 260=South, 261=Central, 262=North, 280=Bamako, 281=Kayes, 282=Koulikoro, 283=Sikasso, 284=Segou, 285=Mopti, 286=Tombouctou, 287=Gao, 288=Kidal, 300=Maputo province, 301=Maputo City, 302=Gaza, 303=Inhambane, 304=Sofala, 305=Tete, 306=Manica, 307=Zambezia, 308=Nampula, 309=Niassa, 310=Cabo Delgado, 320=Caprivi, 321=Erongo, 322=Hardap, 323=Karas, 324=Kavango, 325=Khomas, 326=Kunene, 327=Ohangwena, 328=Omaheke, 329=Omusati, 330=Oshana, 331=Oshikoto, 332=Otjozundjupa, 340=Lagos, 341=Ogun, 342=Oyo, 343=Osun, 344=Ondo, 345=Ekiti, 346=Enugu, 347=Anambra, 348=Imo, 349=Abia, 350=Akwa-Ibom, 351=Bayelsa, 352=Cross-River, 353=Delta, 354=Edo, 355=Rivers, 356=Kano, 357=Sokoto, 358=Kaduna, 359=Katsina, 360=Dakar, 361=Diourbel, 362=Fatick, 363=Kaolack, 364=Kolda, 365=Louga, 366=Matam, 367=Saint Louis, 368=Tambacounda, 369=Thies,

370=Ziguinchor, 380=Dodoma, 381=Arusha, 382=Kilimanjaro, 383=Tanga, 384=Morogoro, 385=Pwani, 386=Dar es Salaam, 387=Lindi, 388=Mtwara, 389=Ruvuma, 390=Iringa, 391=Mbeya, 392=Singida, 393=Tabora, 394=Rukwa, 395=Kigoma, 396=Shinyanga, 397=Kagera, 398=Mwanza, 399=Mara, 400=Central, 401=East, 402=North, 404=West, 420=Lusaka, 421=Central, 422=Copper Belt, 423=Eastern, 424=Luapula, 425=Northern, 426=North-western, 427=Southern,428=Western, 440=Harare, 441=Bulawayo, 442=Midlands, 443=Masvingo, 444=Mashonaland East, 445=Mashonaland West, 446= Mashonaland Central, 447= Matebeleland South, 448= Matebeleland North, 449=Manicaland, 540=Manyara, 541=North Unguja, 542=South Unguja, 543=Urban West, 544=North Pemba, 545=South Pemba, 550=Zamfara, 551=Bauchi, 552=Bomo, 553=Adamawa, 554=Taraba, 555=Plateau, 556=Benue, 557=Kogi, 558=Kwara, 559=Niger, 560=FCT
Note: Answered by interviewer

Question Number: district
Question: District
Variable Label: District
Values: String variable
Note: Answered by interviewer

Question Number: hholdse1
Question: Reasons for unsuccessful calls – Household 1
Variable Label: Reason for Unsuccessful Call 1
Values: 1-8, 997, -1
Value Labels: 1=Refused to be interviewed, 2=Person selected was never at home, 3=Household/Premises empty for the survey period, 4=Not a citizen/Spoke only a foreign language, 5=Deaf/Did not speak a survey language, 6=Did not fit gender quota, 7=No adults in household, 8=Other, 997=Not Applicable, -1=Missing Data
Source: Southern Africa Barometer (SAB)
Note: Answered by interviewer, after instructions, "It is your job is to select a random (this means any) household. A household is a group of people who presently eat together from the same spot. Start your walk pattern from the start point that has been randomly chosen by your Field Supervisor. Team members must walk in opposite directions to each other. If A walks towards the sun, B must walk away from the sun; C and D must walk at right angles to A and B. Use the day code to determine the sampling interval. For example, on the 5th, 14th and 23rd of the month, the day code (and sampling interval) is five. So you choose the fifth dwelling structure on the right. On the 6th, 15th and 24th of the month, the sampling interval is six. So you choose the sixth dwelling structure on the right. And so on. If a call is unsuccessful, use the table below to record your progress until you make a successful call. Circle a code number for unsuccessful calls only."

Question Number: hholdse2
Question: Reasons for unsuccessful calls – Household 2
Variable Label: Reason for Unsuccessful Call 2
Values: 1-8, 997
Value Labels: 1=Refused to be interviewed, 2=Person selected was never at home, 3=Household/Premises empty for the survey period, 4=Not a citizen/Spoke only a foreign language, 5=Deaf/Did not speak a survey language, 6=Did not fit gender quota, 7=No adults in household, 8=Other, 997=Not Applicable
Source: SAB

Question Number: hholdse3
Question: Reasons for unsuccessful calls – Household 3
Variable Label: Reason for Unsuccessful Call 3
Values: 1-8, 997
Value Labels: 1=Refused to be interviewed, 2=Person selected was never at home, 3=Household/Premises empty for the survey period, 4=Not a citizen/Spoke only a foreign language, 5=Deaf/Did not speak a survey language, 6=Did not fit gender quota, 7=No adults in household, 8=Other, 997=Not Applicable
Source: SAB
Note: Answered by interviewer

Question Number: hholdse4
Question: Reasons for unsuccessful calls – Household 4
Variable Label: Reason for Unsuccessful Call 4
Values: 1-8, 997

Copyright Afrobarometer

Value Labels: 1=Refused to be interviewed, 2=Person selected was never at home, 3=Household/Premises empty for the survey period, 4=Not a citizen/Spoke only a foreign language, 5=Deaf/Did not speak a survey language, 6=Did not fit gender quota, 7=No adults in household, 8=Other, 997=Not Applicable
Source: SAB
Note: Answered by interviewer

Question Number: hholdse5
Question: Reasons for unsuccessful calls – Household 5
Variable Label: Reason for Unsuccessful Call 5
Values: 1-8, 997
Value Labels: 1=Refused to be interviewed, 2=Person selected was never at home, 3=Household/Premises empty for the survey period, 4=Not a citizen/Spoke only a foreign language, 5=Deaf/Did not speak a survey language, 6=Did not fit gender quota, 7=No adults in household, 8=Other, 997=Not Applicable
Source: SAB
Note: Answered by interviewer

Question Number: hholdse6
Question: Reasons for unsuccessful calls – Household 6
Variable Label: Reason for Unsuccessful Call 6
Values: 1-8, 997
Value Labels: 1=Refused to be interviewed, 2=Person selected was never at home, 3=Household/Premises empty for the survey period, 4=Not a citizen/Spoke only a foreign language, 5=Deaf/Did not speak a survey language, 6=Did not fit gender quota, 7=No adults in household, 8=Other, 997=Not Applicable
Source: SAB
Note: Answered by interviewer

Question Number: hholdse7
Question: Reasons for unsuccessful calls – Household 7
Variable Label: Reason for Unsuccessful Call 7
Values: 1-8, 997
Value Labels: 1=Refused to be interviewed, 2=Person selected was never at home, 3=Household/Premises empty for the survey period, 4=Not a citizen/Spoke only a foreign language, 5=Deaf/Did not speak a survey language, 6=Did not fit gender quota, 7=No adults in household, 8=Other, 997=Not Applicable
Source: SAB
Note: Answered by interviewer

Question Number: prevint
Question: Previous interview was with a:
Variable Label: Previous interview, gender
Values: 0, 1, 2,
Value Labels: 0=First interview, 1=Male, 2=Female
Note: Question not asked in Ghana

Question Number: currint
Question: This interview must be with a:
Variable Label: This interview, gender
Values: 1, 2
Value Labels: 1=Male, 2=Female
Note: Answered by interviewer

Question Number: calls
Question: Interviewer: How many calls were made to the household where the interview actually took place?
Variable Label: Number of calls
Values: 1, 2, -1
Value Labels: 1=One call, 2=Two calls, -1=Missing Data
Source: SAB
Note: Answered by interviewer

Question Number: dateintr
Question: Date of interview
Variable Label: Date of interview
Values: 10Mar2005-07Mar2006
Note: Answered by interviewer.

Question Number: strtime
Question: Time interview started
Variable Label: Time interview started
Note: Answered by interviewer. Entered hour and minute, 24 hour clock

Question Number: Q1
Question: How old are you?
Variable Label: Age
Values: 18-110, 998, 999, -1
Value Labels: 998=Refused to Answer, 999=Don't Know, -1=Missing Data

Question Number: Q2
Question: Are you the head of the household?
Variable Label: Head of household
Values: 0, 1, 9, 998, -1
Value Labels: 0=No, 1=Yes, 9=Don't know, 998=Refused to answer, -1=Missing Data

Question Number: Q3
Question: Which [Ghanaian/Kenyan/etc.] language is your home language?
Variable Label: Language of respondent
Values: 1-4, 100-109, 120-130, 140-149, 165, 180-185, 200-221, 240-244, 260-271, 280-298, 300-312, 320-332, 338, 340-369, 380-398, 405-429, 431-444, 500-511, 550-569, 580, 803-820, 860-866, 995, 998-999, -1
Value Labels: 1=English, 2=French, 3=Portuguese, 4=Kiswahili, 100=Afrikaans, 101=Ndebele, 102=Xhosa, 103=Pedi/Spedi/North Sotho, 104=Sesotho/Sotho/South Sotho, 105=Setswana/Tswana, 106=Shangaan, 107=Swazi, 108=Venda, 109=Zulu, 120=Fon, 121=Adja, 122=Bariba, 123=Dendi, 124=Yoruba, 125=Ditamari, 126=Peulh, 127=Yoa, 128=Haoussa, 129=Idé, 130=Lamba, 140=Setswana, 141=Sesarwa, 142=Sekgaladi, 143=Sesobea, 144=Sekalanga, 145=Seherero, 146=Sembukushu, 147=Sebirwa, 148=Sengologa, 149=Seyei, 165=Creole, 180=Akan, 181=Ewe, 182=Ga/Dangbe, 183=Other Northern Languages, 184=Dagbani, 185=Dagaare, 200=Kikuyu, 201=Luo, 202=Luhya, 203=Kamba, 204=Meru/Embu, 205=Kisli, 206=Kalenjin, 207=Masai/Samburu, 208=MijiKenda, 209=Taita, 210=Somali, 211=Pokot, 212=Turkana, 213=Digo, 214=Giriama, 215=Duruma, 216=Chonyi, 217=Gunya, 218=Arabic, 219=Maragoli, 220=Sesotho, 221=Sephuthi, 240=Malgache officielle, 241=Langue regionale, 242=Chinois, 243=Comorien, 244=Pakistanais, 260=Tumbuka, 261=Nkhonde, 262=Lambya, 263=Chewa, 264=Yao, 265=Ngoni, 266=Lomwe, 267=Manga'nja, 268=Sena, 269=Sukwa, 270=Senga, 271=Tonga, 280=Bambara, 281=Peugl/Fulfulde, 282=Senufo, 283=Mianka, 284=Mossi, 285=Soninke, 286=Malinke, 287=Khasonke, 288=Dogon, 289=Bobo, 290=Bozo, 291=Arabe, 292=Maure, 293=Kakolo, 294=Samoko, 295=Sonrhai, 296=Bella, 297=Tamasheq, 298=Dafing, 300=Makua, 301=Sena, 302=Ndau, 303=Nyanja, 304=Changana, 305=Chope, 306=Bitonga, 307=Makonde, 308=Chuabo, 309=Ajua, 310=Quimuane, 311=Muaui, 312=Nhungue, 320=Nama/Damara, 321=Afrikaans, 322=Rukwangali, 323=Silozi, 324=Masubia, 325=German, 326=Oshiwambo, 327=Otjiherero, 328=Setswana , 329=Angolan:Portuguese, 330=Angolan:Rumbundu, 331=Other Kavango:Thimbukushu, 332=Other Kavango: Rugririku, 338=Ndebele, 340=Hausa, 341=Igbo, 342=Yoruba, 343=Pidgin English, 344=Efik, 345=Ebira, 346=Fulani, 347=Isoko, 348=Ibibio, 349=Kanuri, 350=Tiv, 351=Nupe, 352=Ijaw, 353=Edo, 354=Igala, 355=Urhobo, 356=Idoma, 357=Bassa, 358=Ikwere, 359=Ukwani, 360=Wolof, 361=Pular, 362=Serer, 363=Mandinka, 364=Sonike, 365=Diola, 366=Manjack, 367=Bambara, 368=Bainouk, 369=Bassari, 380=Kinyakyusa, 381=Kichaga, 382=Kihaya, 383=Kingoni, 384=Kikwere, 385=Kipare, 386=Kihehe, 387=Kimakonde, 388=Kinyamwezi, 389=Kisukuma, 390=Kimasai, 391=Kimeru, 392=Kikurya, 393=Kigogo, 394=Kiluguru, 395=Kifipa, 396=Kimanyema, 397=Kinyiramba, 398=Kinyaturu, 405=Akaramojong, 406=Akur, 407=Ateso, 408=Japadhola, 409=Jonam, 410=Kakwa, 411=Kumam, 412=Kuksabin, 413=Luo, 414=Lugbara, 415=Lugwere, 416=Madi, 417=Luganda, 418=Lugishi/Lumasaba, 419=Lusoga, 420=Bemba, 421=Nyanja, 422=Tonga, 423=Lozi, 424=Chewa, 425=Nsenga, 426=Tumbuka, 427=Kaonde, 428=Luvale, 429=Namwanga, 431=Bisa, 432=Ila, 433=Nkaya, 434=Mambwe, 435=Lunda, 436=Lenje, 437=Lamba, 438=Mbunda, 439=Ngoni, 440=Ndebele, 441=Shona, 442=Venda, 443=Kalanga, 444=Tonga, 500=Lunyoli, 501=Rufumbira, 502=Ruhororo, 503=Rukiga,

504=Rukonzo, 505=Runyarwandam 506=Runyoro, 507=Rutagwenda, 508=Rutooro, 509=Rwamba, 510=Runyankole, 511=Samia, 550=Anang, 551=Ron, 552=Geomai, 553=Kadara, 554=Pyam, 555=Bahumono, 556=Boki, 557=Yakurr, 558=Esan, 559=Bijim, 560=Ekpeye, 561=Jaba, 562=Birom, 563=Igede, 564=Pyem, 565=Taroh, 566=Korro, 567=Ogoni, 568=Mbembe, 569=Sayawa, 580=Itsekiri, 803=Tanchoni, 804=Bukusu, 805=Nyore, 806=Marama, 807=Sabaot, 808=Wanga, 809=Kabarasi, 810=Nyala, 811=Vatsotso, 812=Keiyo, 813=Kipsigis, 814=Tugen, 815=Boran, 816=Ethiopian/Kenyan, 817=Indian, 818=Burji, 819=Kuria, 820=Gare, 860=Soli, 861=Senga, 862=Chokwe, 863=Luchazi. 864=Kwamashi, 865=Tokaleya, 866=Lala, 995=other, 998=Refused to answer, 999=Don't Know, -1=Missing Data
Source: SAB

Question Number: Q4A
Question: In general, how would you describe: The present economic conditions of this country?
Variable Label: Country's present economic condition
Values: 1-5, 9, 998, -1
Value Labels: 1=Very bad, 2=Fairly bad, 3=Neither good nor bad, 4=Fairly good, 5=Very good, 9=Don't Know, 998=Refused to Answer, -1=Missing Data
Source: NDB, Zambia96

Question Number: Q4B
Question: In general, how would you describe: Your own present living conditions?
Variable Label: Your present living conditions
Values: 1-5, 9, 998, -1
Value Labels: 1=Very bad, 2=Fairly bad, 3=Neither good nor bad, 4=Fairly good, 5=Very good, 9=Don't Know, 998=Refused to Answer, -1=Missing Data
Source: NDB, Zambia96

Question Number: Q5
Question: In general, how do you rate: Your living conditions compared to those of other [Ghanaians/Kenyans/etc.]?
Variable Label: Your living conditions vs. others
Values: 1-5, 9, 98, -1
Value Labels: 1=Much worse, 2=Worse, 3=Same, 4=Better, 5=Much better, 9=Don't Know, 98=Refused to Answer, -1=Missing Data
Source: NDB, Zambia96

Question Number: Q6A
Question: Looking back, how do you rate the following compared to twelve months ago: Economic conditions in this country?
Variable Label: Country's economic condition 12 months ago
Values: 1-5, 9, 998, -1
Value Labels: 1=Much worse, 2=Worse, 3=Same, 4=Better, 5=Much better, 9=Don't Know, 998=Refused to Answer, -1=Missing Data
Source: SAB

Question Number: Q6B
Question: Looking back, how do you rate the following compared to twelve months ago: Your living conditions?
Variable Label: Your living conditions 12 months ago
Values: 1-5, 9, 998, -1
Value Labels: 1=Much worse, 2=Worse, 3=Same, 4=Better, 5=Much better, 9=Don't Know, 998=Refused to Answer, -1=Missing Data
Source: NDB, Zambia96

Question Number: Q7A
Question: Looking ahead, do you expect the following to be better or worse: Economic conditions in this country in twelve months time?
Variable Label: Country's economic condition in 12 months
Values: 1-5, 9, 998, -1

Copyright Afrobarometer

Value Labels: 1=Much worse, 2=Worse, 3=Same, 4=Better, 5=Much better, 9=Don't Know, 998=Refused to Answer, -1=Missing Data
Source: NDB, Zambia96

Question Number: Q7B
Question: Looking ahead, do you expect the following to be better or worse: Your living conditions in twelve months time?
Variable Label: Your living conditions in 12 months
Values: 1-5, 9, 998, -1
Value Labels: 1=Much worse, 2=Worse, 3=Same, 4=Better, 5=Much better, 9=Don't Know, 998=Refused to Answer, -1=Missing Data
Source: NDB, Zambia96

Question Number: Q8A
Question: Over the past year, how often, if ever, have you or anyone in your family gone without: Enough food to eat?
Variable Label: How often gone without food
Values: 0-4, 9, 998, -1
Value Labels: 0=Never, 1=Just once or twice, 2=Several times, 3=Many times, 4=Always, 9=Don't Know, 998=Refused to Answer, -1=Missing Data
Source: NDB

Question Number: Q8B
Question: Over the past year, how often, if ever, have you or anyone in your family gone without: Enough clean water for home use?
Variable Label: How often gone without water
Values: 0-4, 9, 998, -1
Value Labels: 0=Never, 1=Just once or twice, 2=Several times, 3=Many times, 4=Always, 9=Don't Know, 998=Refused to Answer, -1=Missing Data
Source: NDB

Question Number: Q8C
Question: Over the past year, how often, if ever, have you or anyone in your family gone without: Medicines or medical treatment?
Variable Label: How often gone without medical care
Values: 0-4, 9, 998, -1
Value Labels: 0=Never, 1=Just once or twice, 2=Several times, 3=Many times, 4=Always, 9=Don't Know, 998=Refused to Answer, -1=Missing Data
Source: NDB

Question Number: Q8D
Question: Over the past year, how often, if ever, have you or anyone in your family gone without: Enough fuel to cook your food?
Variable Label: How often gone without cooking fuel
Values: 0-4, 9, 998, -1
Value Labels: 0=Never, 1=Just once or twice, 2=Several times, 3=Many times, 4=Always, 9=Don't Know, 998=Refused to Answer, -1=Missing Data
Source: SAB

Question Number: Q8E
Question: Over the past year, how often, if ever, have you or anyone in your family gone without: A cash income?
Variable Label: How often gone without cash income
Values: 0-4, 9, 998, -1
Value Labels: 0=Never, 1=Just once or twice, 2=Several times, 3=Many times, 4=Always, 9=Don't Know, 998=Refused to Answer, -1=Missing Data
Source: SAB

Copyright Afrobarometer

Question Number: Q8F
Question: Over the past year, how often, if ever, have you or anyone in your family gone without: School expenses for your children (like fees, uniforms or books)?
Variable Label: How often gone without school expenses
Values: 0-4, 7, 9, 998, -1
Value Labels: 0=Never, 1=Just once or twice, 2=Several times, 3=Many times, 4=Always, 7=No children, 9=Don't Know, 998=Refused to Answer, -1=Missing Data
Source: SAB

Question Number: Q9A
Question: Over the past year, how often (if ever) have you or anyone in your family: Feared crime in your own home?
Variable Label: How often feared crime in home
Values: 0-4, 9, 998, -1
Value Labels: 0=Never, 1=Just once or twice, 2=Several times, 3=Many times, 4=Always, 9=Don't Know, 998=Refused to Answer, -1=Missing Data
Source: Adapted from NDB
Note: Question not asked in Zimbabwe.

Question Number: Q9B
Question: Over the past year, how often (if ever) have you or anyone in your family: Had something stolen from your house?
Variable Label: How often something stolen from house
Values: 0-4, 9, 998, -1
Value Labels: 0=Never, 1=Just once or twice, 2=Several times, 3=Many times, 4=Always, 9=Don't Know, 998=Refused to Answer, -1=Missing Data
Source: Adapted from NDB
Note: Question not asked in Zimbabwe.

Question Number: Q9C
Question: Over the past year, how often (if ever) have you or anyone in your family: Been physically attacked?
Variable Label: How often physically attacked
Values: 0-4, 9, 998, -1
Value Labels: 0=Never, 1=Just once or twice, 2=Several times, 3=Many times, 4=Always, 9=Don't Know, 998=Refused to Answer, -1=Missing Data
Source: Adapted from NDB
Note: Question not asked in Zimbabwe.

Question Number: Q10
Question: Which of the following statements is closest to your view? Choose Statement A or Statement B.
A: It is better to have free schooling for our children, even if the quality of education is low.
B: It is better to raise educational standards, even if we have to pay school fees.
Variable Label: Free schooling vs. user fees
Values: 1-5, 9, 98, -1
Value Labels: 1=Agree Very Strongly with A, 2=Agree with A, 3=Agree with B, 4=Agree Very Strongly with B, 5=Agree with Neither, 9=Don't Know, 98=Refused to Answer, -1=Missing Data
Source: Zambia96
Note: Interviewer probed for strength of opinion. Question not asked in Zimbabwe.

Question Number: Q11
Question: Which of the following statements is closest to your view? Choose Statement A or Statement B.
A: All civil servants should keep their jobs, even if paying their salaries is costly to the country.
B: The government cannot afford so many public employees and should lay some of them off.
Variable Label: Civil servants keep jobs vs. lay offs to reduce costs
Values: 1-5, 9, 98, -1
Value Labels: 1=Agree Very Strongly with A, 2=Agree with A, 3=Agree with B, 4=Agree Very Strongly with B, 5=Agree with Neither, 9=Don't Know, 98=Refused to Answer, -1=Missing Data
Source: Zambia 96

Copyright Afrobarometer

Note: Interviewer probed for strength of opinion. Question not asked in Zimbabwe.

Question Number: Q12
Question: Which of the following statements is closest to your view? Choose Statement A or Statement B.
A: The costs of reforming the economy are too high; the government should therefore abandon its current economic policies.
B: In order for the economy to get better in the future, it is necessary for us to accept some hardships now.
Variable Label: Abandon economic reforms vs. accept hardships
Values: 1-5, 9, 98, -1
Value Labels: 1=Agree Very Strongly with A, 2=Agree with A, 3=Agree with B, 4=Agree Very Strongly with B, 5=Agree with Neither, 9=Don't Know, 98=Refused to Answer, -1=Missing Data
Source: Ghana97
Note: Interviewer probed for strength of opinion.

Question Number: Q13
Question: Which of the following statements is closest to your view? Choose Statement A or Statement B.
A: The government's economic policies have helped most people; only a few have suffered.
B: The government's economic policies have hurt most people and only benefited a few.
Variable Label: Economic policies helped most vs. hurt most
Values: 1-5, 9, 98, -1
Value Labels: 1=Agree Very Strongly with A, 2=Agree with A, 3=Agree with B, 4=Agree Very Strongly with B, 5=Agree with Neither, 9=Don't Know, 98=Refused to Answer, -1=Missing Data
Source: Adapted from Ghana99
Note: Interviewer probed for strength of opinion.

Question Number: Q14A
Question: Please tell me whether each of the following aspects of our economic situation in this country are better or worse than they were a few years ago, or whether they have remained the same: The availability of consumer goods?
Variable Label: Present vs. past: Availability of goods
Values: 1-5, 9, 98, -1
Value Labels: 1= Much Worse, 2=Worse, 3=About the Same, 4=Better, 5=Much Better, 9=Don't Know, 98=Refused to Answer, -1=Missing Data
Source: Afrobarometer Round 2
Note: Interviewer probed for strength of opinion.

Question Number: Q14B
Question: Please tell me whether each of the following aspects of our economic situation in this country are better or worse than they were a few years ago, or whether they have remained the same: The availability of job opportunities?
Variable Label: Present vs. past: Job opportunities
Values: 1-5, 9, 98, -1
Value Labels: 1= Much Worse, 2=Worse, 3=About the Same, 4=Better, 5=Much Better, 9=Don't Know, 98=Refused to Answer, -1=Missing Data
Source: Afrobarometer Round 2
Note: Interviewer probed for strength of opinion.

Question Number: Q14C
Question: Please tell me whether each of the following aspects of our economic situation in this country are better or worse than they were a few years ago, or whether they have remained the same: The gap between the rich and the poor?
Variable Label: Present vs. past: Gap between rich and poor
Values: 1-5, 9, 98, -1
Value Labels: 1= Much Worse, 2=Worse, 3=About the Same, 4=Better, 5=Much Better, 9=Don't Know, 98=Refused to Answer, -1=Missing Data
Source: Afrobarometer Round 2
Note: Interviewer probed for strength of opinion.

Copyright Afrobarometer

Question Number: Q15A
Question: How often do you get news from the following sources: Radio?
Variable Label: Radio news
Values: 0-4, 9, 98, -1
Value Labels: 0=Never, 1=Less than once a month, 2=A few times a month, 3=A few times a week, 4=Every day, 9=Don't Know, 98=Refused to Answer, -1=Missing Data
Source: Zambia96

Question Number: Q15B
Question: How often do you get news from the following sources: Television?
Variable Label: Television news
Values: 0-4, 9, 98, -1
Value Labels: 0=Never, 1=Less than once a month, 2=A few times a month, 3=A few times a week, 4=Every day, 9=Don't Know, 98=Refused to Answer, -1=Missing Data
Source: SAB

Question Number: Q15C
Question: How often do you get news from the following sources: Newspapers?
Variable Label: Newspaper news
Values: 0-4, 9, 98, -1
Value Labels: 0=Never, 1=Less than once a month, 2=A few times a month, 3=A few times a week, 4=Every day, 9=Don't Know, 98=Refused to Answer, -1=Missing Data
Source: Zambia96

Question Number: Q16
Question: How interested would you say you are in public affairs?
Variable Label: Interest in public affairs
Values: 0-3, 9, 98, -1
Value Labels: 0=Not at all interested, 1=Not very interested, 2=Somewhat interested, 3=Very interested, 9=Don't Know, 98=Refused to Answer, -1=Missing Data
Source: SAB

Question Number: Q17
Question: When you get together with your friends or family, would you say you discuss political matters:
Variable Label: Discuss politics
Values: 0-2, 9, 98, -1
Value Labels: 0=Never, 1=Occasionally, 2=Frequently, 9=Don't Know, 98=Refused to Answer, -1=Missing Data

Question Number: Q18A
Question: Do you agree or disagree with the following statements: Politics and government sometimes seem so complicated that you can't really understand what's going on?
Variable Label: Politics and government too complicated
Values: 1-5, 9, 98, -1
Value Labels: 1=Strongly Agree, 2=Agree, 3=Neither Agree nor Disagree, 4=Disagree, 5=Strongly Disagree, 9=Don't Know, 98=Refused to Answer, -1=Missing Data
Source: Zambia96
Note: Interviewer probed for strength of opinion.

Question Number: Q18B
Question: Do you agree or disagree with the following statements: As far as politics is concerned, friends and neighbors do not listen to you?
Variable Label: Others don't listen to you
Values: 1-5, 9, 98, -1
Value Labels: 1=Strongly Agree, 2=Agree, 3=Neither Agree nor Disagree, 4=Disagree, 5=Strongly Disagree, 9=Don't Know, 98=Refused to Answer, -1=Missing Data
Source: Zambia96
Note: Interviewer probed for strength of opinion.

Copyright Afrobarometer

Question Number: Q19
Question: Lets talk for a moment about the kind of society we would like to have in this country. Which of the following statements is closest to your view? Choose Statement A or Statement B.
A: People should look after themselves and be responsible for their own success in life.
B: The government should bear the main responsibility for the well-being of people.
Variable Label: People responsible for well-being vs. government
Values: 1-5, 9, 98, -1
Value Labels: 1=Agree Very Strongly with A, 2=Agree with A, 3=Agree with B, 4=Agree Very Strongly with B, 5=Agree with Neither, 9=Don't Know, 98=Refused to Answer, -1=Missing Data
Source: Ghana99
Note: Interviewer probed for strength of opinion.
Note: Question not asked in Zimbabwe.

Question Number: Q20
Question: Let's talk for a moment about the kind of society we would like to have in this country. Which of the following statements is closest to your view? Choose Statement A or Statement B.
A: As citizens, we should be more active in questioning the actions of our leaders.
B: In our country these days, we should show more respect for authority.
Variable Label: Question actions of leaders vs. respect authority
Values: 1-5, 9, 98, -1
Value Labels: 1=Agree Very Strongly with A, 2=Agree with A, 3=Agree with B, 4=Agree Very Strongly with B, 5=Agree with Neither, 9=Don't Know, 98=Refused to Answer, -1=Missing Data
Source: Zambia96
Note: Interviewer probed for strength of opinion, asking "Do you agree or agree very strongly?" Question not asked in Zimbabwe.

Question Number: Q21
Question: Which of the following statements is closest to your view? Choose Statement A or Statement B.
A: Since leaders represent everyone, leaders should not favour their own family or group.
B: Once in office, leaders are obliged to help their home community.
Variable Label: Leaders treat all equally vs. help own community
Values: 1-5, 9, 98, -1
Value Labels: 1=Agree Very Strongly with A, 2=Agree with A, 3=Agree with B, 4=Agree Very Strongly with B, 5=Agree with Neither, 9=Don't Know, 98=Refused to Answer, -1=Missing Data
Source: Afrobarometer Round 2
Note: Interviewer probed for strength of opinion. Question not asked in Zimbabwe.

Question Number: Q22
Question: Which of the following statements is closest to your view? Choose Statement A or Statement B.
A: All people should be permitted to vote, even if they do not fully understand all the issues in an election.
B: Only those who are sufficiently well educated should be allowed to choose our leaders.
Variable Label: All vote vs. only educated vote
Values: 1-5, 9, 98, -1
Value Labels: 1=Agree Very Strongly with A, 2=Agree with A, 3=Agree with B, 4=Agree Very Strongly with B, 5=Agree with Neither, 9=Don't Know, 98=Refused to Answer, -1=Missing Data
Source: Zambia 96
Note: Interviewer probed for strength of opinion. Question not asked in Zimbabwe.

Question Number: Q23
Question: Which of the following statements is closest to your view? Choose Statement A or Statement B.
A: In our country, women should have equal rights and receive the same treatment as men do.
B: Women have always been subject to traditional laws and customs, and should remain so.
Variable Label: Women have equal rights vs. subject to traditional laws
Values: 1-5, 9, 98, -1
Value Labels: 1=Agree Very Strongly with A, 2=Agree with A, 3=Agree with B, 4=Agree Very Strongly with B, 5=Agree with Neither, 9=Don't Know, 98=Refused to Answer, -1=Missing Data
Source: Uganda00
Note: Interviewer probed for strength of opinion. Question not asked in Zimbabwe.

Copyright Afrobarometer

Question Number: Q24
Question: Which of the following statements is closest to your view? Choose Statement A or Statement B.
A: Women should have the same chance of being elected to political office as men.
B: Men make better political leaders than women, and should be elected rather than women.
Variable Label: Women leaders vs. men only as leaders
Values: 1-5, 9, 98, -1
Value Labels: 1=Agree Very Strongly with A, 2=Agree with A, 3=Agree with B, 4=Agree Very Strongly with B, 5=Agree with Neither, 9=Don't Know, 98=Refused to Answer, -1=Missing Data
Source: Uganda00
Note: Interviewer probed for strength of opinion.

Question Number: Q25
Question: Which of the following statements is closest to your view? Choose Statement A or Statement B.
A: Government should be able to ban any organization that goes against its policies.
B: We should be able to join any organization, whether or not the government approves of it.
Variable Label: Government bans organization vs. join any
Values: 1-5, 9, 98, -1
Value Labels: 1=Agree Very Strongly with A, 2=Agree with A, 3=Agree with B, 4=Agree Very Strongly with B, 5=Agree with Neither, 9=Don't Know, 98=Refused to Answer, -1=Missing Data
Source: Uganda00
Note: Interviewer probed for strength of opinion.

Question Number: Q26
Question: Which of the following statements is closest to your view? Choose Statement A or Statement B.
A: Government should close newspapers that print false stories or misinformation.
B: The news media should be free to publish any story that they see fit without fear of being shut down.
Variable Label: Government close newspapers vs. free to publish
Values: 1-5, 9, 98, -1
Value Labels: 1=Agree Very Strongly with A, 2=Agree with A, 3=Agree with B, 4=Agree Very Strongly with B, 5=Agree with Neither, 9=Don't Know, 98=Refused to Answer, -1=Missing Data
Source: Uganda00
Note: Interviewer probed for strength of opinion.

Question Number: Q27
Question: Which of the following statements is closest to your view? Choose Statement A or Statement B.
A: Government should not allow the expression of political views that are fundamentally different from the views of the majority.
B: People should be able to speak their minds about politics free of government influence, no matter how unpopular their views may be.
Variable Label: Government suppress expression vs. people speak minds
Values: 1-5, 9, 98, -1
Value Labels: 1=Agree Very Strongly with A, 2=Agree with A, 3=Agree with B, 4=Agree Very Strongly with B, 5=Agree with Neither, 9=Don't Know, 98=Refused to Answer, -1=Missing Data
Source: Uganda00
Note: Interviewer probed for strength of opinion.

Question Number: Q28A
Question: Let's turn to your role in the community. Now I am going to read out a list of groups that people join or attend. For each one, could you tell me whether you are an official leader, an active member, an inactive member, or not a member: A religious group (e.g. church, mosque)?
Variable Label: Member of religious group
Values: 0-3, 9, 98, -1
Value Labels: 0=Not a Member, 1=Inactive Member, 2=Active Member, 3=Official Leader, 9=Don't Know, 98=Refused to Answer, -1=Missing Data
Source: SAB

Copyright Afrobarometer

Question Number: Q28B
Question: Now I am going to read out a list of groups that people join or attend. For each one, could you tell me whether you are an official leader, an active member, an inactive member, or not a member: A trade union or farmers association?
Variable Label: Member of trade union or farmers association
Values: 0-3, 9, 98, -1
Value Labels: 0=Not a Member, 1=Inactive Member, 2=Active Member, 3=Official Leader, 9=Don't Know, 98=Refused to Answer, -1=Missing Data
Source: SAB

Question Number: Q28C
Question: Now I am going to read out a list of groups that people join or attend. For each one, could you tell me whether you are an official leader, an active member, an inactive member, or not a member: A professional or business association?
Variable Label: Member of professional or business association
Values: 0-3, 9, 98, -1
Value Labels: 0=Not a Member, 1=Inactive Member, 2=Active Member, 3=Official Leader, 9=Don't Know, 98=Refused to Answer, -1=Missing Data
Source: SAB

Question Number: Q28D
Question: Now I am going to read out a list of groups that people join or attend. For each one, could you tell me whether you are an official leader, an active member, an inactive member, or not a member: A community development or self-help association?
Variable Label: Member of community development association
Values: 0-3, 9, 98, -1
Value Labels: 0=Not a Member, 1=Inactive Member, 2=Active Member, 3=Official Leader, 9=Don't Know, 98=Refused to Answer, -1=Missing Data
Source: SAB

Question Number: Q29
Question: Understanding that some [Ghanaians/Kenyans/etc.] were not able to register as voters for the [20xx] elections, which statement is true for you?
Variable Label: Registered to vote
Values: 1-6, 9, 98, -1
Value Labels: 1=You were registered to vote, 2=You did not want to register, 3=You could not find a place to register, 4=You were prevented from registering, 5=You were too young to register, 6= Did not register for some other reason, 9=Don't Know/Can't remember, 98=Refused to Answer, -1=Missing Data
Source: Afrobarometer Round 3
Note: Last national election was in 2000 in Senegal and Tanzania; 2001 in Zambia; 2002 in Cape Verde, Kenya, Lesotho, Madagascar, Mali, and Uganda; 2003 in Benin and Nigeria, 2004 in Botswana, Ghana, Malawi, Mozambique, Namibia, and South Africa; and 2005 in Zimbabwe

Question Number: Q30
Question: With regard to the most recent, [20xx] national elections, which statement is true for you?
Variable Label: Voted last election
Values: 1-7, 9, 98, -1
Value Labels: 1=You voted in the elections, 2=You decided not to vote, 3=You could not find the polling station, 4=You were prevented from voting, 5=You did not have time to vote 6= Did not vote for some other reason, 7=You were not registered 9=Don't Know/Can't remember, 98=Refused to Answer, -1=Missing Data
Source: Afrobarometer Round 3
Note: Last national election was in 2000 in Senegal and Tanzania; 2001 in Zambia; 2002 in Cape Verde, Kenya, Lesotho, Madagascar, Mali, and Uganda; 2003 in Benin and Nigeria, 2004 in Botswana, Ghana, Malawi, Mozambique, Namibia, and South Africa; and 2005 in Zimbabwe.

Copyright Afrobarometer

Question Number: Q31A
Question: Here is a list of actions that people sometimes take as citizens. For each of these, please tell me whether you, personally, have done any of these things during the past year. If not, would you do this if you had the chance: Attended a community meeting?
Variable Label: Attend a community meeting
Values: 0-4, 9, 98, -1
Value Labels: 0=No, would never do this, 1=No, but would do if had the chance, 2=Yes, once or twice, 3=Yes, several times, 4=Yes, often, 9=Don't Know, 98=Refused to Answer, -1=Missing Data
Source: SAB

Question Number: Q31B
Question: Here is a list of actions that people sometimes take as citizens. For each of these, please tell me whether you, personally, have done any of these things during the past year. If not, would you do this if you had the chance: Got together with others to raise an issue?
Variable Label: Join others to raise an issue
Values: 0-4, 9, 98, -1
Value Labels: 0=No, would never do this, 1=No, but would do if had the chance, 2=Yes, once or twice, 3=Yes, several times, 4=Yes, often, 9=Don't Know, 98=Refused to Answer, -1=Missing Data
Source: SAB

Question Number: Q31C
Question: Here is a list of actions that people sometimes take as citizens. For each of these, please tell me whether you, personally, have done any of these things during the past year. If not, would you do this if you had the chance: Attended a demonstration or protest march?
Variable Label: Attend a demonstration or protest march
Values: 0-4, 9, 98, -1
Value Labels: 0=No, would never do this, 1=No, but would do if had the chance, 2=Yes, once or twice, 3=Yes, several times, 4=Yes, often, 9=Don't Know, 98=Refused to Answer, -1=Missing Data
Source: Zambia96

Question Number: Q32A
Question: During the past year, how often have you contacted any of the following persons for help to solve a problem or to give them your views: A Local Government Councillor?
Variable Label: Contact Local government councillor
Values: 0-3, 9, 98, -1
Value Labels: 0=Never, 1=Only once, 2=A few times, 3=Often, 9=Don't Know, 98=Refused to Answer, -1=Missing Data
Source: Adapted from Zambia96
Note: Question not asked in Zimbabwe.
Note: "Local Government Councillor" in Botswana, Kenya, Malawi, Namibia, Nigeria, South Africa, Tanzania, Uganda, and Zambia; "District Chief Executive" in Ghana and Mozambique; "Village Development Councillor" in Lesotho; "Conseiller Communal" in Benin, Madagascar and Mali; "Um deputado da Assembleia Municipal" in Cape Verde.

Question Number: Q32B
Question: During the past year, how often have you contacted any of the following persons for help to solve a problem or to give them your views: A Member of Parliament/National Assembly Representative?
Variable Label: Contact MP
Values: 0-3, 9, 98, -1
Value Labels: 0=Never, 1=Only once, 2=A few times, 3=Often, 9=Don't Know, 98=Refused to Answer, -1=Missing Data
Source: Adapted from Zambia96
Note: Question not asked in Zimbabwe.
Note: "National Assembly Representative" in Benin, Cape Verde, Madagascar, Mali, Mozambique, Namibia, Nigeria, Senegal and South Africa; "Member of Parliament" in Botswana, Ghana, Kenya, Lesotho, Malawi, Tanzania, Uganda, Zambia, and Zimbabwe.

Question Number: Q32C
Question: During the past year, how often have you contacted any of the following persons for help to solve a problem or to give them your views: An official of a government ministry?
Variable Label: Contact official of a government ministry
Values: 0-3, 9, 98, -1
Value Labels: 0=Never, 1=Only once, 2=A few times, 3=Often, 9=Don't Know, 98=Refused to Answer, -1=Missing Data
Source: Adapted from Zambia96
Note: Question not asked in Zimbabwe.

Question Number: Q32D
Question: During the past year, how often have you contacted any of the following persons for help to solve a problem or to give them your views: A political party official?
Variable Label: Contact political party official
Values: 0-3, 9, 98, -1
Value Labels: 0=Never, 1=Only once, 2=A few times, 3=Often, 9=Don't Know, 98=Refused to Answer, -1=Missing Data
Source: Adapted from Zambia96
Note: Question not asked in Zimbabwe.

Question Number: Q32E
Question: During the past year, how often have you contacted any of the following persons for help to solve a problem or to give them your views: A religious leader?
Variable Label: Contact religious leader
Values: 0-3, 9, 98, -1
Value Labels: 0=Never, 1=Only once, 2=A few times, 3=Often, 9=Don't Know, 98=Refused to Answer, -1=Missing Data
Source: Adapted from Zambia96
Note: Question not asked in Zimbabwe.

Question Number: Q32F
Question: During the past year, how often have you contacted any of the following persons for help to solve a problem or to give them your views: A traditional ruler?
Variable Label: Contact traditional ruler
Values: 0-3, 7, 9, 98, -1
Value Labels: 0=Never, 1=Only once, 2=A few times, 3=Often, 7=Not Applicable, 9=Don't Know, 98=Refused to Answer, -1=Missing Data
Source: Adapted from Zambia96
Note: Question not asked in Cape Verde and Zimbabwe.

Question Number: Q32G
Question: During the past year, how often have you contacted any of the following persons for help to solve a problem or to give them your views: Some other influential person?
Variable Label: Contact some other influential person
Values: 0-3, 9, 98, -1
Value Labels: 0=Never, 1=Only once, 2=A few times, 3=Often, 9=Don't Know, 98=Refused to Answer, -1=Missing Data
Source: Adapted from Zambia96
Note: Question not asked in Zimbabwe.

Question Number: Q33
Question: Think of the last time you contacted any of the above leaders. Was the main reason to:
Variable Label: Why contact
Values: 1-4, 7, 9, 98, -1
Value Labels: 1=Tell them about personal problems, 2= Tell them about a community or public problem, 3= Give your view on political issue 4=Something else, 9=Don't Know, 98=Refused to Answer, -1=Missing Data
Note: Question not asked in Zimbabwe.

Copyright Afrobarometer

Question Number: Q34
Question: What if anything does democracy mean to you?
Variable Label: Understand democracy
Values: 1-3, 9, -1
Value Labels: 1=Understood “democracy” in national language, 2= Required local language translation, 3=Did not understand the word or question, even in the local language, -1=Missing
Source: Afrobarometer Round 3
Note: Interviewer was instructed as follows: “Read the question in the language of the interview, but always state the word ;democracy’ in the national language (i.e., English, French, Portuguese or Kiswahili). Only translate into local language if respondent does not understand the word ‘democracy.’ Record whether respondent understood word in national language or required a local language translation.”

Question Number: Q35A
Question: What if anything does democracy mean to you?
Variable Label: Meaning of democracy-first verbatim response
Values: String variable
Source: Ghana 97
Note: Respondent could give up to three responses. Interviewer recorded respondent’s verbatim response in the national language (i.e., English, French, Portuguese or Kiswahili). Responses were coded by Field Supervisors and checked by National Investigator.

Question Number: Q35pt1
Question: What if anything does democracy mean to you?
Variable Label: First meaning of democracy (code)
Values: 0-12, 14-17, 19-21, 995, 998, 999, -1
Value Labels: 0=Nothing/Democracy has no meaning, 1=Civil liberties/personal freedoms (e.g. freedom of speech, religion, movement, etc…), 2=Government by, for, of the people/popular rule, 3=Voting/elections/multiparty competition, 4=Peace/unity/power sharing, 5=Social/economic development, 6=Equality/justice, 7=Majority rule, 8=Governance/effectiveness/accountability/rule of law, 9=National independence/people’s self-determination=9, 10=Mutual respect, 11=Working together, 12=Other positive meanings, 14=Conflict/confusion, 15=Corruption/abuse of power, 16=Social/economic hardship, 17=Other negative meanings, 19=Civilian politics/government, 20=Change of government/leadership/laws, 21=Other neutral/ null meanings, 995=Other, 998=Refused to answer, 999=Don't know/Did not understand the question, -1=Missing data
Note: Respondent could give up to three responses. Interviewer recorded respondent’s verbatim response in the national language (i.e., English, French, Portuguese or Kiswahili). Responses were coded by Field Supervisors and checked by National Investigator.

Question Number: Q35B
Question: What if anything does democracy mean to you?
Variable Label: Meaning of democracy-second verbatim response
Values: String variable
Source: Ghana 97
Note: Respondent could give up to three responses. Interviewer recorded respondent’s verbatim response in the national language (i.e., English, French, Portuguese or Kiswahili). Responses were coded by Field Supervisors and checked by National Investigator.

Question Number: Q35pt2
Question: What if anything does democracy mean to you?
Variable Label: Second meaning of democracy (code)
Values: 1-12, 14-17, 19-21, 996, -1
Value Labels: 1=Civil liberties/personal freedoms (e.g. freedom of speech, religion, movement, etc…), 2=Government by, for, of the people/popular rule, 3=Voting/elections/multiparty competition, 4=Peace/unity/power sharing, 5=Social/economic development, 6=Equality/justice, 7=Majority rule, 8=Governance/effectiveness/accountability/rule of law, 9=National independence/people’s self-determination=9, 10=Mutual respect, 11=Working together, 12=Other positive meanings, 14=Conflict/confusion, 15=Corruption/abuse of power, 16=Social/economic hardship, 17=Other negative meanings, 19=Civilian

Copyright Afrobarometer

politics/government, 20=Change of government/leadership/laws, 21=Other neutral/ null meanings, 996=No Further Answer, -1=Missing data
Note: Respondent could give up to three responses. Interviewer recorded respondent's verbatim response in the national language (i.e., English, French, Portuguese or Kiswahili). Responses were coded by Field Supervisors and checked by National Investigator.

Question Number: Q35C
Question: What if anything does democracy mean to you?
Variable Label: Meaning of democracy-third verbatim response
Values: String variable
Source: Ghana 97
Note: Respondent could give up to three responses. Interviewer recorded respondent's verbatim response in the national language (i.e., English, French, Portuguese or Kiswahili). Responses were coded by Field Supervisors and checked by National Investigator.

Question Number: Q35pt3
Question: What if anything does democracy mean to you?
Variable Label: Third meaning of democracy (code)
Values: 1-12, 14-17, 19-21, 996, -1
Value Labels: 1=Civil liberties/personal freedoms (e.g., freedom of speech, religion, movement, etc…), 2=Government by, for, of the people/popular rule, 3=Voting/elections/multiparty competition, 4=Peace/unity/power sharing, 5=Social/economic development, 6=Equality/justice, 7=Majority rule, 8=Governance/effectiveness/accountability/rule of law, 9=National independence/people's self-determination=9, 10=Mutual respect, 11=Working together, 12=Other positive meanings, 14=Conflict/confusion, 15=Corruption/abuse of power, 16=Social/economic hardship, 17=Other negative meanings, 19=Civilian politics/government, 20=Change of government/leadership/laws, 21=Other neutral/ null meanings, 996=No Further Answer, -1=Missing data
Source: Ghana 97
Note: Respondent could give up to three responses. Interviewer recorded respondent's verbatim response in the national language (i.e., English, French, Portuguese or Kiswahili). Responses were coded by Field Supervisors and checked by National Investigator.

Question Number: Q36A
Question: There are many ways to govern a country. Would you disapprove or approve of the following alternatives: Only one political party is allowed to stand for election and hold office?
Variable Label: Reject one-party rule
Values: 1-5, 9, 98, -1
Value Labels: 1=Strongly Disapprove, 2=Disapprove, 3=Neither Approve Nor Disapprove, 4=Approve, 5=Strongly Approve, 9=Don't Know, 98=Refused to Answer, -1=Missing Data
Source: NDB
Note: Interviewer probed for strength of opinion.

Question Number: Q36B
Question: There are many ways to govern a country. Would you disapprove or approve of the following alternatives: The army comes in to govern the country?
Variable Label: Reject military rule
Values: 1-5, 9, 98, -1
Value Labels: 1=Strongly Disapprove, 2=Disapprove, 3=Neither Approve Nor Disapprove, 4=Approve, 5=Strongly Approve, 9=Don't Know, 98=Refused to Answer, -1=Missing Data
Source: Adapted from NDB
Note: Interviewer probed for strength of opinion.

Question Number: Q36C
Question: There are many ways to govern a country. Would you disapprove or approve of the following alternatives: Elections and Parliament/National Assembly are abolished so that the President/Prime Minister can decide everything?
Variable Label: Reject one-man rule
Values: 1-5, 9, 98, -1

Value Labels: 1=Strongly Disapprove, 2=Disapprove, 3=Neither Approve Nor Disapprove, 4=Approve, 5=Strongly Approve, 9=Don't Know, 98=Refused to Answer, -1=Missing Data
Source: SAB
Note: Interviewer probed for strength of opinion. "National Assembly" in Benin, Cape Verde, Madagascar, Mali, Mozambique, Namibia, Nigeria, Senegal and South Africa; "Parliament" in Botswana, Ghana, Kenya, Lesotho, Malawi, Tanzania, Uganda, Zambia, and Zimbabwe. "Prime Minister" in Lesotho; "President" in all other countries.

Question Number: Q37
Question: Which of these three statements is closest to your own opinion?
A: Democracy is preferable to any other kind of government.
B: In some circumstances, a non-democratic government can be preferable.
C: For someone like me, it doesn't matter what kind of government we have.
Variable Label: Support for democracy
Values: 1-3, 9, 98, -1
Value Labels: 1=Statement C: Doesn't matter, 2=Statement B: Sometimes non-democratic preferable, 3=Statement A: Democracy preferable, 9=Don't Know, 98=Refused to Answer, -1=Missing Data
Source: Latinobarometer (LB)
Note: Interviewer stated the word "democracy" in national language (i.e., English, French, Portuguese or Kiswahili), not in a local language.

Question Number: Q38
Question: Let's change the subject. I would like to hear your views about how this country is governed. Which of the following statements is closest to your view? Choose Statement A or Statement B.
A: We should choose our leaders in this country through regular, open and honest elections.
B: Since elections sometimes produce bad results, we should adopt other methods for choosing this country's leaders.
Variable Label: Choose leaders through elections vs. other methods
Values: 1-5, 9, 98, -1
Value Labels: 1=Agree Very Strongly with A, 2=Agree with A, 3=Agree with B, 4=Agree Very Strongly with B, 5=Agree with Neither, 9=Don't Know, 98=Refused to Answer, -1=Missing Data
Source: Afrobarometer Round 2
Note: Interviewer probed for strength of opinion, asking "Do you agree or agree very strongly?"

Question Number: Q39
Question: Which of the following statements is closest to your view? Choose Statement A or Statement B.
A: Political parties create division and confusion; it is therefore unnecessary to have many political parties in [Ghana/Kenya/etc.].
B: Many political parties are needed to make sure that [Ghanaians/Kenyans/etc.] have real choices in who governs them.
Variable Label: Political parties divisive vs. many parties needed
Values: 1-5, 9, 98, -1
Value Labels: 1=Agree Very Strongly with A, 2=Agree with A, 3=Agree with B, 4=Agree Very Strongly with B, 5=Agree with Neither, 9=Don't Know, 98=Refused to Answer, -1=Missing Data
Source: Afrobarometer Round 2
Note: Interviewer probed for strength of opinion, asking "Do you agree or agree very strongly?"

Question Number: Q40
Question: Which of the following statements is closest to your view? Choose Statement A or Statement B.
A: The members of Parliament/National Assembly represent the people; therefore they should make laws for this country, even if the President/Prime Minister does not agree.
B: Since the President/Prime Minister represents all of us, he should pass laws without worrying about what the Parliament/National Assembly thinks.
Variable Label: Parliament makes laws vs. president does
Values: 1-5, 9, 98, -1
Value Labels: 1=Agree Very Strongly with A, 2=Agree with A, 3=Agree with B, 4=Agree Very Strongly with B, 5=Agree with Neither, 9=Don't Know, 98=Refused to Answer, -1=Missing Data
Source: Afrobarometer Round 2

Copyright Afrobarometer

Note: Interviewer probed for strength of opinion, asking "Do you agree or agree very strongly?" "National Assembly" in Benin, Cape Verde, Madagascar, Mali, Mozambique, Namibia, Nigeria, Senegal and South Africa; "Parliament" in Botswana, Ghana, Kenya, Lesotho, Malawi, Tanzania, Uganda, Zambia, and Zimbabwe. "Prime Minister" in Lesotho; "President" in all other countries.

Question Number: Q41
Question: Which of the following statements is closest to your view? Choose Statement A or Statement B.
A: Since the President/Prime Minister was elected to lead the country, he should not be bound by laws or court decisions that he thinks are wrong.
B: The President/Prime Minister must always obey the laws and the courts, even if he thinks they are wrong.
Variable Label: President free to act vs. obey the laws and courts
Values: 1-5, 9, 98, -1
Value Labels: 1=Agree Very Strongly with A, 2=Agree with A, 3=Agree with B, 4=Agree Very Strongly with B, 5=Agree with Neither, 9=Don't Know, 98=Refused to Answer, -1=Missing Data
Note: Interviewer probed for strength of opinion, asking "Do you agree or agree very strongly?" "Prime Minister" in Lesotho; "President" in all other countries.

Question Number: Q42
Question: Which of the following statements is closest to your view? Choose Statement A or Statement B.
A: Our present system of elected government should be given more time to deal with inherited problems.
B: If our present system cannot produce results soon, we should try another form of government.
Variable Label: Time to deal with problems vs. try another form
Values: 1-5, 9, 98, -1
Value Labels: 1=Agree Very Strongly with A, 2=Agree with A, 3=Agree with B, 4=Agree Very Strongly with B, 5=Agree with Neither, 9=Don't Know, 98=Refused to Answer, -1=Missing Data
Note: Interviewer probed for strength of opinion, asking "Do you agree or agree very strongly?"

Now let us speak about the political system in this country?

Question Number: 43A1
Question: Can you tell me the name of: Your Member of Parliament/National Assembly Representative?
Variable Label: Identify MP (verbatim)
Values: String variable
Source: Afrobarometer Round 3
Note: Question not asked in Zimbabwe. Interviewer preceded question with the statement: "Now let us speak about the political system in this country." Interviewer recorded respondent's verbatim response, and then coded the answer. "National Assembly Representative" in Benin, Cape Verde, Lesotho, Madagascar, Mali, Namibia, Nigeria, Senegal and South Africa; "Member of Parliament" in Botswana, Ghana, Kenya, Malawi, Mozambique, Tanzania, Uganda and Zambia.

Question Number: 43A2
Question: Can you tell me the name of: Your Member of Parliament/National Assembly Representative?
Variable Label: Identify MP (code)
Values: 1-3, 9, 98, -1
Value Labels: 1=Know but can't remember, 2=Incorrect guess, 3=Correct name, 9=Don't Know, 98=Refused to Answer, -1=Missing Data
Source: Afrobarometer Round 3
Note: Question not asked in Zimbabwe. Interviewer preceded question with the statement: "Now let us speak about the political system in this country." Interviewer recorded respondent's verbatim response, and then coded the answer. "National Assembly Representative" in Benin, Cape Verde, Lesotho, Madagascar, Mali, Namibia, Nigeria, Senegal and South Africa; "Member of Parliament" in Botswana, Ghana, Kenya, Malawi, Mozambique, Tanzania, Uganda and Zambia.

Question Number: 43B1
Question: Can you tell me the name of: Your Local Government Councillor?
Variable Label: Identify Local Government Councillor (verbatim)
Values: String variable
Source: Afrobarometer Round 3

Note: Interviewer recorded respondent's verbatim response, and then coded the answer. Question not asked in Zimbabwe. "Local Government Councillor" in Botswana, Kenya, Malawi, Namibia, Nigeria, South Africa, Tanzania, Uganda, and Zambia; "District Chief Executive" in Ghana and Mozambique; "Village Development Councillor" in Lesotho; "Maire de votre commune" in Benin, Madagascar, and Mali; "Presidente da Assembleia Municipal" in Cape Verde.

Question Number: 43B2
Question: Can you tell me the name of: Your Local Government Councillor?
Variable Label: Identify local government councilor (code)
Values: 1-3, 7, 9, 98, -1
Value Labels: 1=Know but can't remember, 2=Incorrect guess, 3=Correct name, 9=Don't Know, 98=Refused to Answer, -1=Missing Data
Source: Afrobarometer Round 3
Note: Interviewer recorded respondent's verbatim response, and then coded the answer. Question not asked in Zimbabwe. "Local Government Councillor" in Botswana, Kenya, Malawi, Namibia, Nigeria, South Africa, Tanzania, Uganda, and Zambia; "District Chief Executive" in Ghana and Mozambique; "Village Development Councillor" in Lesotho; "Maire de votre commune" in in Benin, Madagascar, and Mali; "Presidente da Assembleia Municipal" in Cape Verde.

Question Number: 43C1
Question: Can you tell me the name of: The Deputy President/Vice President?
Variable Label: Identify Deputy President (verbatim)
Values: String Variable
Source: Afrobarometer Round 3
Note: Interviewer recorded respondent's verbatim response, and then coded the answer. Question not asked in Zimbabwe. "Votre préfet" in Benin; "President of the National Assembly" in Cape Verde, Madagascar, Mali, Mozambique and Senegal; "Vice President" in Botswana, Ghana, Kenya, Malawi, Nigeria, Tanzania and Zambia; "Deputy President" in South Africa and Uganda; "Deputy Prime Minister" in Lesotho; "Prime Minister" in Namibia. Correct answer is Seretse Khama Ian Khama in Botswana; Aristides Lima in Cape Verde; Alhaji Aliu Mahama in Ghana; Moody Awori in Kenya; Lesao Lehohla in Lesotho; Jean Lahiniriko in Madagascar; Cassim Chilumpa in Malawi; Ibrahim Boubacar Keita in Mali; Eduardo Joaquim Mumlembwe in Mozambique; Nahas Angula in Namibia; Alhaji Atiku Abubakar in Nigeria; Pape Diop in Senegal; Pumzile Mlambo-Ngcuka in South Africa; Ali Mohamed Shein in Tanzania; Dr. Gilbert Bukenya in Uganda; Lupando Mwape in Zambia. In Benin, this is a local government position and there are multiple correct responses.
Namibia.

Question Number: 43C2
Question: Can you tell me the name of: The Deputy President/Vice President?
Variable Label: Identify Deputy President (code)
Values: 1-3, 9, 98, -1
Value Labels: 1=Know but can't remember, 2=Incorrect guess, 3=Correct name, 9=Don't Know, 98=Refused to Answer, -1=Missing Data
Source: Afrobarometer Round 3
Note: Interviewer recorded respondent's verbatim response, and then coded the answer. Question not asked in Zimbabwe. "Votre préfet" in Benin; "President of the National Assembly" in Cape Verde, Madagascar, Mali, Mozambique and Senegal; "Vice President" in Botswana, Ghana, Kenya, Malawi, Nigeria, Tanzania and Zambia; "Deputy President" in South Africa and Uganda; "Deputy Prime Minister" in Lesotho; "Prime Minister" in Namibia. Correct answer is Seretse Khama Ian Khama in Botswana; Aristides Lima in Cape Verde; Alhaji Aliu Mahama in Ghana; Moody Awori in Kenya; Lesao Lehohla in Lesotho; Jean Lahiniriko in Madagascar; Cassim Chilumpa in Malawi; Ibrahim Boubacar Keita in Mali; Eduardo Joaquim Mumlembwe in Mozambique; Nahas Angula in Namibia; Alhaji Atiku Abubakar in Nigeria; Pape Diop in Senegal; Pumzile Mlambo-Ngcuka in South Africa; Ali Mohamed Shein in Tanzania; Dr. Gilbert Bukenya in Uganda; Lupando Mwape in Zambia. In Benin, this is a local government position and there are multiple correct responses.

Question Number: 44A1
Question: Do you happen to know: Which political party has the most seats in parliament/the national assembly?
Variable Label: Know: political party w/most seats (verbatim)
Values: String Variable

Source: Afrobarometer Round 3
Note: Question not asked in Zimbabwe. "National Assembly" in Benin, Madagascar, Mali, Namibia, Nigeria, Senegal and South Africa; "Parliament" in Botswana, Cape Verde, Ghana, Kenya, Lesotho, Malawi, Mozambique, Tanzania, Uganda and Zambia. Correct answer is Presidential Movement (UBF, MADEP, FC, IDP, and four small parties) in Benin; BDP in Botswana; PAICV in Cape Verde; New Patriotic Party or NPP in Ghana; NARC in Kenya; Lesotho Congress for Democracy (LCD) in Lesotho; TIM in Madagascar; MCP in Malawi; Hope 2002 Coalition (RPM, CNID, MPR and RDT) in Mali; Frelimo in Mozambique; SWAPO in Namibia; PDP in Nigeria; PDS in Senegal; ANC in South Africa; CCM in Tanzania; NRMO in Uganda; and MMD in Zambia.

Question Number: 44A2
Question: Do you happen to know: Which political party has the most seats in parliament/national assembly?
Variable Label: Know: political party w/most seats (code)
Values: 1-3, 9, 98, -1
Value Labels: 1=Know but can't remember, 2=Incorrect guess, 3=Correct name, 9=Don't Know, 98=Refused to Answer, -1=Missing Data
Source: Afrobarometer Round 3
Note: Question not asked in Zimbabwe. "National Assembly" in Benin, Madagascar, Mali, Namibia, Nigeria, Senegal and South Africa; "Parliament" in Botswana, Cape Verde, Ghana, Kenya, Lesotho, Malawi, Mozambique, Tanzania, Uganda and Zambia. Correct answer is Presidential Movement (UBF, MADEP, FC, IDP, and four small parties) in Benin; BDP in Botswana; PAICV in Cape Verde; New Patriotic Party or NPP in Ghana; NARC in Kenya; Lesotho Congress for Democracy (LCD) in Lesotho; TIM in Madagascar; MCP in Malawi; Hope 2002 Coalition (RPM, CNID, MPR and RDT) in Mali; Frelimo in Mozambique; SWAPO in Namibia; PDP in Nigeria; PDS in Senegal; ANC in South Africa; CCM in Tanzania; NRMO in Uganda; and MMD in Zambia.

Question Number: 44B1
Question: Do you happen to know: How many times someone can legally be elected President/Prime Minister?
Variable Label: Know: Presidential term limits (verbatim)
Values: String variable
Source: Afrobarometer Round 3
Note: Question not asked in Zimbabwe. The word "legally" was not included in Benin, Botswana, Cape Verde, Ghana, Lesotho, Madagascar, Malawi, Mali, Mozambique, Tazania, and Uganda. "Prime Minister" in Lesotho; "President" in all other countries. Correct answer is no limit in Lesotho; three terms in Mozambique; and two terms everywhere else.

Question Number: 44B2
Question: Do you happen to know: How many times someone can be elected President/Prime Minister?
Variable Label: Know: Presidential term limits (code)
Values: 1-3, 9, 98, -1
Value Labels: 1=Know but can't remember, 2=Incorrect guess, 3=Correct name, 9=Don't Know, 98=Refused to Answer, -1=Missing Data
Source: Afrobarometer Round 3
Note: Question not asked in Zimbabwe. The word "legally" was not included in Benin, Botswana, Cape Verde, Ghana, Lesotho, Madagascar, Malawi, Mali, Mozambique, Tazania, and Uganda. "Prime Minister" in Lesotho; "President" in all other countries. Correct answer is no limit in Lesotho; three terms in Mozambique; and two terms everywhere else.

Question Number: 44C1
Question: Do you happen to know: Whose responsibility it is to determine whether or not a law is constitutional?
Variable Label: Know: Determine constitutionality (verbatim)
Values: String variable
Source: Afrobarometer Round 3
Note: Question not asked in Zimbabwe. In general, because of the generally low level of knowledge on this question, "correct" responses were broadly interpreted to include most responses that identified the courts/judiciary (as opposed to e.g., parliament, president, citizens, etc.), even if they did not mention the specific name of the court that is ultimately responsible for these decisions. Please note that some corrections have been made to the coding of responses on this question since individual country-data sets were released, so there may be some differences with the findings in country data sets.

Copyright Afrobarometer

Question Number: 44C2
Question: Do you happen to know: Whose responsibility it is to determine whether or not a law is constitutional?
Variable Label: Know: Determine constitutionality (code)
Values: 1-3, 9, 98, -1
Value Labels: 1=Know but can't remember, 2=Incorrect guess, 3=Correct name, 9=Don't Know, 98=Refused to Answer, -1=Missing Data
Source: Afrobarometer Round 3
Note: Question not asked in Zimbabwe. In general, because of the generally low level of knowledge on this question, "correct" responses were broadly interpreted to include most responses that identified the courts/judiciary (as opposed to e.g., parliament, president, citizens, etc.), even if they did not mention the specific name of the court that is ultimately responsible for these decisions. Please note that some corrections have been made to the coding of responses on this question since individual country-data sets were released, so there may be some differences with the findings in country data sets.

Question Number: Q45
Question: On the whole, how would you rate the freeness and fairness of the last national election, held in [20xx]. Was it:
Variable Label: Elections free and fair
Values: 1-4, 8, 9, 98, -1
Value Labels: 1=completely free and fair, 2=Free and fair, but with minor problems, 3=Free and Fair, with major problems, 4=Not free and fair, 8=Do not understand questions, 9=Don't Know, 98=Refused to Answer, -1=Missing Data
Note: Last election was 2000 in Senegal and Tanzania; 2001 in Zambia; 2002 in Cape Verde, Kenya, Lesotho, Madagascar, Mali, and Uganda; 2003 in Benin and Nigeria, 2004 in Botswana, Ghana, Malawi, Mozambique, Namibia, and South Africa; and 2005 in Zimbabwe

Question Number: Q46
Question: In your opinion how much of a democracy is [Ghana/Kenya/etc.] today?
Variable Label: Extent of democracy
Values: 1-4, 8, 9, 98, -1
Value Labels: 1=Not a democracy, 2=A democracy, with major problems, 3=A democracy, but with minor problems, 4=A full democracy, 8=Do not understand question/democracy, 9=Don't Know, 98=Refused to Answer, -1=Missing Data
Source: Ghana 97
Note: Interviewer stated the word "democracy" in national language (i.e., English, French, Portuguese or Kiswahili), not in a local language.

Question Number: Q47
Question: Overall, how satisfied are you with the way democracy works in [Ghana/Kenya/etc.]? Are you:
Variable Label: Satisfaction with democracy
Values: 0-4, 9, 98, -1
Value Labels: 0=My country is not a democracy, 1=Not at All Satisfied, 2=Not Very Satisfied, 3=Fairly Satisfied, 4=Very Satisfied, 9=Don't Know, 98=Refused to Answer, -1=Missing Data
Source: Eurobarometer
Note: Interviewer stated the word "democracy" in national language (i.e., English, French, Portuguese or Kiswahili), not in a local language.

Question Number: Q48
Question: In your opinion, how likely is it that [Ghana/Kenya, etc.] will remain a democratic country?
Variable Label: Future of democracy
Values: 0-4, 9, 98, -1
Value Labels: 0=Country is not a democracy, 1=Not at All Likely, 2=Not Very Likely, 3=Likely, 4=Very Likely, 9=Don't Know, 98=Refused to Answer, -1=Missing Data
Note: Question not asked in Zimbabwe.

Question Number: Q49
Question: Which of the following statements is closest to your view? Choose Statement A or Statement B.
A: It is important to obey the government in power no matter who you voted for.

B: It is not necessary to obey the laws of a government that I did not vote for.
Variable Label: Obey government always vs. only if vote for it
Values: 1-5, 9, 98, -1
Value Labels: 1=Agree Very Strongly with A, 2=Agree with A, 3=Agree with B, 4=Agree Very Strongly with B, 5=Agree with Neither, 9=Don’t Know, 98=Refused to Answer, -1=Missing Data
Note: Interviewer probed for strength of opinion, asking “Do you agree or agree very strongly?”

Question Number: Q50
Question: Which of the following statements is closest to your view? Choose Statement A or Statement B.
A: It is better to find lawful solutions to problems even if it takes longer.
B: It is sometimes better to ignore the law and solve problems immediately using other means
Variable Label: Lawful vs. immediate solutions to problems
Values: 1-5, 9, 98, -1
Value Labels: 1=Agree Very Strongly with A, 2=Agree with A, 3=Agree with B, 4=Agree Very Strongly with B, 5=Agree with Neither, 9=Don’t Know, 98=Refused to Answer, -1=Missing Data
Note: Interviewer probed for strength of opinion, asking “Do you agree or agree very strongly?”

Question Number: Q51
Question: Which of the following statements is closest to your view. Choose Statement A or Statement B.
A: The use of violence is never justified in [Ghanaian/Kenyan/etc.] politics.
B: In this country, it is sometimes necessary to use violence in support of a just cause.
Variable Label: Violence never justified vs. sometimes necessary
Values: 1-5, 9, 98, -1
Value Labels: 1=Agree Very Strongly with A, 2=Agree with A, 3=Agree with B, 4=Agree Very Strongly with B, 5=Agree with Neither, 9=Don’t Know, 98=Refused to Answer, -1=Missing Data
Source: Zambia96
Note: Interviewer probed for strength of opinion, asking “Do you agree or agree very strongly?”

Question Number: Q52A
Question: For each of the following statements, please tell me whether you disagree or agree: Our constitution expresses the values and hopes of the [Ghanaian/Kenyan/etc.] people.
Variable Label: Constitution expresses values and hopes
Values: 1-5, 9, 98, -1
Value Labels: 1=Strongly Disagree, 2=Disagree, 3=Neither Agree Nor Disagree, 4=Agree, 5=Strongly Agree, 9=Don’t Know, 98=Refused to Answer, -1=Missing Data
Source: SAB
Note: The interviewer probed for strength of opinion.

Question Number: Q52B
Question: For each of the following statements, please tell me whether you disagree or agree: The courts have the right to make decisions that people always have to abide by.
Variable Label: Courts make binding decisions
Values: 1-5, 9, 98, -1
Value Labels: 1=Strongly Disagree, 2=Disagree, 3=Neither Agree Nor Disagree, 4=Agree, 5=Strongly Agree, 9=Don’t Know, 98=Refused to Answer, -1=Missing Data
Source: Afrobarometer Round 2
Note: The interviewer probed for strength of opinion.

Question Number: Q52C
Question: For each of the following statements, please tell me whether you disagree or agree: The police always have the right to make people obey the law.
Variable Label: People must obey the law
Values: 1-5, 9, 98, -1
Value Labels: 1=Strongly Disagree, 2=Disagree, 3=Neither Agree Nor Disagree, 4=Agree, 5=Strongly Agree, 9=Don’t Know, 98=Refused to Answer, -1=Missing Data
Source: Afrobarometer Round 2
Note: The interviewer probed for strength of opinion.

Copyright Afrobarometer

Question Number: Q52D
Question: For each of the following statements, please tell me whether you disagree or agree: The tax department always has the right to make people pay taxes.
Variable Label: People must pay taxes
Values: 1-5, 9, 98, -1
Value Labels: 1=Strongly Disagree, 2=Disagree, 3=Neither Agree Nor Disagree, 4=Agree, 5=Strongly Agree, 9=Don't Know, 98=Refused to Answer, -1=Missing Data
Source: Afrobarometer Round 2
Note: The interviewer probed for strength of opinion.

Question Number: Q53A
Question: In this country, how often: Do people have to be careful of what they say about politics?
Variable Label: Careful about what you say
Values: 0-3, 9, 98, -1
Value Labels: 0=Never, 1=Rarely, 2=Often, 3=Always, 9=Don't Know, 98=Refused to Answer, -1=Missing Data
Source: SAB

Question Number: Q53B
Question: In this country, how often: Does competition between political parties lead to conflict?
Variable Label: Party competition leads to conflict
Values: 0-3, 9, 98, -1
Value Labels: 0=Never, 1=Rarely, 2=Often, 3=Always, 9=Don't Know, 98=Refused to Answer, -1=Missing Data
Source: Afrobarometer Round 2

Question Number: Q53C
Question: In this country, how often: Does the President/Prime Minister ignore the constitution?
Variable Label: President ignores the constitution
Values: 0-3, 9, 98, -1
Value Labels: 0=Never, 1=Rarely, 2=Often, 3=Always, 9=Don't Know, 98=Refused to Answer, -1=Missing Data
Source: Afrobarometer Round 2
Note: "Prime Minister" in Lesotho; "President" in all other countries.

Question Number: Q53D
Question: In this country, how often: Are people treated unequally under the law?
Variable Label: People are treated unequally
Values: 0-3, 9, 98, -1
Value Labels: 0=Never, 1=Rarely, 2=Often, 3=Always, 9=Don't Know, 98=Refused to Answer, -1=Missing Data
Source: Afrobarometer Round 2

Question Number: Q54A
Question: Please tell me if the following things are worse or better now than they were a few years ago, or are they about the same: Freedom to say what you think?
Variable Label: Present vs. past: Free to say what you think
Values: 1-5, 9, 98, -1
Value Labels: 1=Much worse, 2=Worse, 3=Same, 4=Better, 5=Much better, 9=Don't Know, 98=Refused to Answer, -1=Missing Data
Source: NDB
Note: Interviewer probed for strength of opinion.

Question Number: Q54B
Question: Please tell me if the following things are worse or better now than they were a few years ago, or are they about the same: Freedom to join any political organization you want?
Variable Label: Present vs. past: Free to join any organization
Values: 1-5, 9, 98, -1
Value Labels: 1=Much worse, 2=Worse, 3=Same, 4=Better, 5=Much better, 9=Don't Know, 98=Refused to Answer, -1=Missing Data
Source: NDB
Note: Interviewer probed for strength of opinion.

Question Number: Q54C
Question: Please tell me if the following things are worse or better now than they were a few years ago, or are they about the same: Freedom from being arrested when you are innocent?
Variable Label: Present vs. past: Fear of unjust arrest
Values: 1-5, 9, 98, -1
Value Labels: 1=Much worse, 2=Worse, 3=Same, 4=Better, 5=Much better, 9=Don't Know, 98=Refused to Answer, -1=Missing Data
Source: SAB
Note: Interviewer probed for strength of opinion.

Question Number: Q54D
Question: Please tell me if the following things are worse or better now than they were a few years ago, or are they about the same: Freedom to choose who to vote for without feeling pressured?
Variable Label: Present vs. past: Voting freedom
Values: 1-5, 9, 98, -1
Value Labels: 1=Much worse, 2=Worse, 3=Same, 4=Better, 5=Much better, 9=Don't Know, 98=Refused to Answer, -1=Missing Data
Source: NDB
Note: Interviewer probed for strength of opinion.

Question Number: Q54E
Question: Please tell me if the following things are worse or better now than they were a few years ago, or are they about the same: The ability of ordinary people to influence what government does?
Variable Label: Present vs. past: Ability to influence government
Values: 1-5, 9, 98, -1
Value Labels: 1=Much worse, 2=Worse, 3=Same, 4=Better, 5=Much better, 9=Don't Know, 98=Refused to Answer, -1=Missing Data
Source: Afrobarometer Round 2
Note: Interviewer probed for strength of opinion.

Question Number: Q54F
Question: Please tell me if the following things are worse or better now than they were a few years ago, or are they about the same: Safety from crime and violence?
Variable Label: Present vs. past: Safety from crime and violence
Values: 1-5, 9, 98, -1
Value Labels: 1=Much worse, 2=Worse, 3=Same, 4=Better, 5=Much better, 9=Don't Know, 98=Refused to Answer, -1=Missing Data
Source: SAB
Note: Interviewer probed for strength of opinion.

Question Number: Q54G
Question: Please tell me if the following things are worse or better now than they were a few years ago, or are they about the same: Equal and fair treatment for all people by government?
Variable Label: Present vs. past: Equal treatment for all
Values: 1-5, 9, 98, -1
Value Labels: 1=Much worse, 2=Worse, 3=Same, 4=Better, 5=Much better, 9=Don't Know, 98=Refused to Answer, -1=Missing Data
Source: NDB
Note: Interviewer probed for strength of opinion.

Question Number: Q55A
Question: How much do you trust each of the following, or haven't you heard enough about them to say: The President/Prime Minister?
Variable Label: Trust the President
Values: 0-3, 9, 98, -1
Value Labels: 0=Not at all, 1=Just a little, 2=Somewhat, 3=A lot, 9=Don't Know/Haven't Heard Enough, 98=Refused to Answer, -1=Missing Data

Copyright Afrobarometer

Source: Zambia96
Note: "Prime Minister" in Lesotho; "President" in all other countries.

Question Number: Q55B
Question: How much do you trust each of the following, or haven't you heard enough about them to say: The Parliament/National Assembly?
Variable Label: Trust Parliament/National Assembly
Values: 0-3, 9, 98, -1
Value Labels: 0=Not at all, 1=Just a little, 2=Somewhat, 3=A lot, 9=Don't Know/Haven't Heard Enough, 98=Refused to Answer, -1=Missing Data
Source: Adapted from Zambia96
Note: "National Assembly" in Benin, Cape Verde, Madagascar, Mali, Mozambique, Namibia, Nigeria, Senegal and South Africa; "Parliament" in Botswana, Ghana, Kenya, Lesotho, Malawi, Tanzania, Uganda, Zambia, and Zimbabwe.

Question Number: Q55C
Question: How much do you trust each of the following, or haven't you heard enough about them to say: The Electoral Commission?
Variable Label: Trust the Electoral Commission
Values: 0-3, 9, 98, -1
Value Labels: 0=Not at all, 1=Just a little, 2=Somewhat, 3=A lot, 9=Don't Know/Haven't Heard Enough, 98=Refused to Answer, -1=Missing Data
Source: Adapted from Zambia96.
Note: The official name of each country's national electoral commission was inserted.

Question Number: Q55D
Question: How much do you trust each of the following, or haven't you heard enough about them to say: Your Elected Local Government Council?
Variable Label: Trust your local council
Values: 0-3, 9, 98, -1
Value Labels: 0=Not at all, 1=Just a little, 2=Somewhat, 3=A lot, 9=Don't Know/Haven't Heard Enough, 98=Refused to Answer, -1=Missing Data
Source: Adapted from Zambia96
Note: "Elected Local Government Council" in Benin, Botswana, Kenya, Madagascar, Malawi, Mali, Mozambique, Namibia, Nigeria, Senegal, South Africa, Tanzania, Uganda, Zambia, and Zimbabwe; "Assembly Men/Women" in Ghana and Mozambique; "Village Development Council" in Lesotho; "Assembleia Municipal" in Cape Verde.

Question Number: Q55E
Question: How much do you trust each of the following, or haven't you heard enough about them to say: The Ruling Party?
Variable Label: Trust the Ruling Party
Values: 0-3, 9, 98, -1
Value Labels: 0=Not at all, 1=Just a little, 2=Somewhat, 3=A lot, 9=Don't Know/Haven't Heard Enough, 98=Refused to Answer, -1=Missing Data
Source: Adapted from Zambia96

Question Number: Q55F
Question: How much do you trust each of the following, or haven't you heard enough about them to say: Opposition Political Parties?
Variable Label: Trust opposition political parties
Values: 0-3, 9, 98, -1
Value Labels: 0=Not at all, 1=Just a little, 2=Somewhat, 3=A lot, 9=Don't Know/Haven't Heard Enough, 98=Refused to Answer, -1=Missing Data
Source: Adapted from Zambia 96

Question Number: Q55G
Question: How much do you trust each of the following, or haven't you heard enough about them to say: The Army?

Copyright Afrobarometer

Variable Label: Trust the military
Values: 0-3, 9, 98, -1
Value Labels: 0=Not at all, 1=Just a little, 2=Somewhat, 3=A lot, 9=Don't Know/Haven't Heard Enough, 98=Refused to Answer, -1=Missing Data
Source: Ghana99

Question Number: Q55H
Question: How much do you trust each of the following, or haven't you heard enough about them to say: The Police?
Variable Label: Trust the police
Values: 0-3, 9, 98, -1
Value Labels: 0=Not at all, 1=Just a little, 2=Somewhat, 3=A lot, 9=Don't Know/Haven't Heard Enough, 98=Refused to Answer, -1=Missing Data
Source: Zambia 96

Question Number: Q55I
Question: How much do you trust each of the following, or haven't you heard enough about them to say: Courts of Law?
Variable Label: Trust courts of law
Values: 0-3, 9, 98, -1
Value Labels: 0=Not at all, 1=Just a little, 2=Somewhat, 3=A lot, 9=Don't Know/Haven't Heard Enough, 98=Refused to Answer, -1=Missing Data
Source: Zambia 96

Question Number: Q55J
Question: How much do you trust each of the following, or haven't you heard enough about them to say: Government broadcasting service (GBC TV or radio)?
Variable Label: Trust government broadcasting service
Values: 0-3, 9, 98, -1
Value Labels: 0=Not at all, 1=Just a little, 2=Somewhat, 3=A lot, 9=Don't Know/Haven't Heard Enough, 98=Refused to Answer, -1=Missing Data
Source: SAB
Note: Specific examples of government broadcast media were inserted for each country.

Question Number: Q55K
Question: How much do you trust each of the following, or haven't you heard enough about them to say: Independent broadcasting services (e.g. TV3 , FM radio)?
Variable Label: Trust independent broadcasting services
Values: 0-3, 9, 98, -1
Value Labels: 0=Not at all, 1=Just a little, 2=Somewhat, 3=A lot, 9=Don't Know/Haven't Heard Enough, 98=Refused to Answer, -1=Missing Data
Source: Zambia96
Note: Specific examples of independent broadcast media were inserted for each country.

Question Number: Q55L
Question: How much do you trust each of the following, or haven't you heard enough about them to say: Government newspapers (Graphic)?
Variable Label: Trust government newspapers
Values: 0-3, 9, 97, 98, -1
Value Labels: 0=Not at all, 1=Just a little, 2=Somewhat, 3=A lot, 9=Don't Know/Haven't Heard Enough, 97=Not asked, 98=Refused to Answer, -1=Missing Data
Source: Zambia96
Note: Question not asked in Kenya, Malawi and South Africa. Specific examples of government newspapers were inserted for each country.

Question Number: Q55M
Question: How much do you trust each of the following, or haven't you heard enough about them to say: Independent newspapers (e.g. Ghanaian Chronicle, Independent)?

Variable Label: Trust independent newspapers
Values: 0-3, 9, 98, -1
Value Labels: 0=Not at all, 1=Just a little, 2=Somewhat, 3=A lot, 9=Don't Know/Haven't Heard Enough, 98=Refused to Answer, -1=Missing Data
Source: Adapted from SAB
Note: Specific examples of independent newspapers were inserted for each country.

Question Number: Q56A
Question: How many of the following people do you think are involved in corruption, or haven't you heard enough about them to say: The President/Prime Minister and Officials in his Office?
Variable Label: Corruption: Office of the Presidency
Values: 0-3, 9, 98, -1
Value Labels: 0=None, 1=Some of them, 2=Most of them, 3=All of them, 9=Don't Know, 98=Refused to Answer, -1=Missing Data
Source: SAB
Note: "Prime Minister" in Lesotho; "President" in all other countries.

Question Number: Q56B
Question: How many of the following people do you think are involved in corruption, or haven't you heard enough about them to say: Members of Parliament/National Assembly Representatives?
Variable Label: Corruption: Members of Parliament
Values: 0-3, 9, 98, -1
Value Labels: 0=None, 1=Some of them, 2=Most of them, 3=All of them, 9=Don't Know, 98=Refused to Answer, -1=Missing Data
Source: SAB
Note: "National Assembly Representatives" in Benin, Madagascar, Mali, Namibia, Nigeria, Senegal and South Africa; "Members of Parliament" in Botswana, Cape Verde, Ghana, Kenya, Lesotho, Malawi, Mozambique, Tanzania, Uganda, Zambia, and Zimbabwe.

Question Number: Q56C
Question: How many of the following people do you think are involved in corruption, or haven't you heard enough about them to say: elected local government councilors?
Variable Label: Corruption: local government councilors
Values: 0-3, 9, 98, -1
Value Labels: 0=None, 1=Some of them, 2=Most of them, 3=All of them, 9=Don't Know, 98=Refused to Answer, -1=Missing Data
Source: SAB
Note: "Elected Local Government Councilors" in Benin, Botswana, Kenya, Madagascar, Malawi, Mali, Mozambique, Namibia, Nigeria, Senegal, South Africa, Tanzania, Uganda, Zambia, and Zimbabwe; "Assembly Man/Woman in Ghana"; "Elected Village Development Councilors" in Lesotho; "Vereadores" in Cape Verde.

Question Number: Q56D
Question: How many of the following people do you think are involved in corruption, or haven't you heard enough about them to say: National government officials?
Variable Label: Corruption: National government officials
Values: 0-3, 9, 98, -1
Value Labels: 0=None, 1=Some of them, 2=Most of them, 3=All of them, 9=Don't Know, 98=Refused to Answer, -1=Missing Data
Source: SAB

Question Number: Q56E
Question: How many of the following people do you think are involved in corruption, or haven't you heard enough about them to say: Local government officials?
Variable Label: Corruption: Local government officials
Values: 0-3, 9, 98, -1
Value Labels: 0=None, 1=Some of them, 2=Most of them, 3=All of them, 9=Don't Know, 98=Refused to Answer, -1=Missing Data
Source: SAB

Copyright Afrobarometer

Question Number: Q56F
Question: How many of the following people do you think are involved in corruption, or haven't you heard enough about them to say: Police?
Variable Label: Corruption: Police
Values: 0-3, 9, 98, -1
Value Labels: 0=None, 1=Some of them, 2=Most of them, 3=All of them, 9=Don't Know, 98=Refused to Answer, -1=Missing Data
Source: SAB

Question Number: Q56G
Question: How many of the following people do you think are involved in corruption, or haven't you heard enough about them to say: Tax Officials (e.g. VATS/IRS officials)
Variable Label: Corruption: Tax Officials
Values: 0-3, 9, 98, -1
Value Labels: 0=None, 1=Some of them, 2=Most of them, 3=All of them, 9=Don't Know, 98=Refused to Answer, -1=Missing Data
Source: SAB
Note: Specific examples of tax agencies were inserted in each country.

Question Number: Q56H
Question: How many of the following people do you think are involved in corruption, or haven't you heard enough about them to say: Judges and Magistrates?
Variable Label: Corruption: Judges and Magistrates
Values: 0-3, 9, 98, -1
Value Labels: 0=None, 1=Some of them, 2=Most of them, 3=All of them, 9=Don't Know, 98=Refused to Answer, -1=Missing Data
Source: SAB

Question Number: Q56I
Question: How many of the following people do you think are involved in corruption, or haven't you heard enough about them to say: Health workers?
Variable Label: Corruption: Health Workers
Values: 0-3, 9, 98, -1
Value Labels: 0=None, 1=Some of them, 2=Most of them, 3=All of them, 9=Don't Know, 98=Refused to Answer, -1=Missing Data
Source: SAB

Question Number: Q56J
Question: How many of the following people do you think are involved in corruption, or haven't you heard enough about them to say: Teachers and school administrators?
Variable Label: Corruption: Teachers and school administrators
Values: 0-3, 9, 98, -1
Value Labels: 0=None, 1=Some of them, 2=Most of them, 3=All of them, 9=Don't Know, 98=Refused to Answer, -1=Missing Data
Source: Adapted from SAB

Question Number: Q57A
Question: In the past year, how often (if ever) have you had to pay a bribe, give a gift, or do a favor to government officials in order to: Get a document or a permit?
Variable Label: Pay bribe for: Document or permit
Values: 0-3, 7, 9, 98, -1
Value Labels: 0=Never, 1=Once or Twice, 2=A Few Times, 3=Often, 7=No experience with this in the past year, 9=Don't Know, 98=Refused to Answer, -1=Missing Data
Source: NDB

Copyright Afrobarometer

Question Number: Q57B
Question: In the past year, how often (if ever) have you had to pay a bribe, give a gift, or do a favor to government officials in order to: Get a child into school?
Variable Label: Pay bribe for: School placement
Values: 0-3, 7, 9, 98, -1
Value Labels: 0=Never, 1=Once or Twice, 2=A Few Times, 3=Often, 7=No experience with this in the past year, 9=Don't Know, 98=Refused to Answer, -1=Missing Data
Source: NDB

Question Number: Q57C
Question: In the past year, how often (if ever) have you had to pay a bribe, give a gift, or do a favor to government officials in order to: Get a household service (like piped water, electricity, or phone)?
Variable Label: Pay bribe for: household service
Values: 0-3, 7, 9, 98, -1
Value Labels: 0=Never, 1=Once or Twice, 2=A Few Times, 3=Often, 7=No experience with this in the past year, 9=Don't Know, 98=Refused to Answer, -1=Missing Data
Source: NDB

Question Number: Q57D
Question: In the past year, how often (if ever) have you had to pay a bribe, give a gift, or do a favor to government officials in order to: Get medicine or medical attention?
Variable Label: Pay bribe for: get medicine or medical attention
Values: 0-3, 7, 9, 98, -1
Value Labels: 0=Never, 1=Once or Twice, 2=A Few Times, 3=Often, 7=No experience with this in the past year, 9=Don't Know, 98=Refused to Answer, -1=Missing Data
Source: NDB

Question Number: Q57E
Question: In the past year, how often (if ever) have you had to pay a bribe, give a gift, or do a favor to government officials in order to: Avoid a problem with the police (like passing a checkpoint or avoiding a fine or arrest)?
Variable Label: Pay bribe for: Avoid problem with police
Values: 0-3, 7, 9, 98, -1
Value Labels: 0=Never, 1=Once or Twice, 2=A Few Times, 3=Often, 7=No experience with this in the past year, 9=Don't Know, 98=Refused to Answer, -1=Missing Data
Source: NDB

Question Number: Q57F
Question: And during the [20xx] election, how often (if ever) did a candidate or someone from a political party offer you something, like food or a gift, in return for your vote?
Variable Label: Election incentives offered
Values: 0-3, 7, 9, 98, -1
Value Labels: 0=Never, 1=Once or Twice, 2=A Few Times, 3=Often, 7=No experience with this in the past year, 9=Don't Know, 98=Refused to Answer, -1=Missing Data
Source: NDB
Note: Last election was 2000 in Senegal and Tanzania; 2001 in Zambia; 2002 in Cape Verde, Kenya, Lesotho, Madagascar, Mali, and Uganda; 2003 in Benin and Nigeria; 2004 in Botswana, Ghana, Malawi, Mozambique, Namibia, and South Africa; and 2005 in Zimbabwe

Question Number: Q58A
Question: For each of the following please indicate whether you think the act is not wrong at all, wrong but understandable, or wrong and punishable: A government official gives a job to someone from his family who does not have adequate qualifications.
Variable Label: Right or wrong: job for relative
Values: 1-3, 9, 98, -1
Value Labels: 1=Not Wrong at All, 2=Wrong But Understandable, 3=Wrong and Punishable, 9=Don't Know, 98=Refused to Answer, -1=Missing Data
Source: Afrobarometer Round 3

Question Number: Q58B
Question: For each of the following please indicate whether you think the act is not wrong at all, wrong but understandable, or wrong and punishable: A government official demands a favour or an additional payment for some service that is part of his job.
Variable Label: Right or wrong: favor for service
Values: 1-3, 9, 98, -1
Value Labels: 1=Not Wrong at All, 2=Wrong But Understandable, 3=Wrong and Punishable, 9=Don't Know, 98=Refused to Answer, -1=Missing Data
Source: Afrobarometer Round 3

Question Number: Q58C
Question: For each of the following please indicate whether you think the act is not wrong at all, wrong but understandable, or wrong and punishable: A public official decides to locate a development project in an area where his friends and supporters lived.
Variable Label: Right or wrong: development for supporters
Values: 1-3, 9, 98, -1
Value Labels: 1=Not Wrong at All, 2=Wrong But Understandable, 3=Wrong and Punishable, 9=Don't Know, 98=Refused to Answer, -1=Missing Data
Source: Afrobarometer Round 3

Question Number: Q59
Question: Which of the following statements is closest to your view? Choose Statement A or Statement B.
A: Our elected officials should listen to constituents' views and do what they demand.
B: Our elected leaders should follow their own ideas in deciding what is best for the country.
Variable Label: Elected leaders listen vs. follow own ideas
Values: 1-5, 9, 98, -1
Value Labels: 1=Agree Very Strongly with A, 2=Agree with A, 3=Agree with B, 4=Agree Very Strongly with B, 5=Agree with Neither, 9=Don't Know, 98=Refused to Answer, -1=Missing Data
Note: Interviewer probed for strength of opinion, asking "Do you agree or agree very strongly?" Question not asked in Zimbabwe.

Question Number: Q60
Question: How much time should your Member of Parliament/National Assembly Representative spend in this constituency to visit the community and its citizens?
Variable Label: Time MP should spend
Values: 0-4, 9, 98, -1
Value Labels: 0=Never/Not Necessary, 1=At Least Once a Year, 2=At Least Once a Month, 3=At Least Weekly, 4= All of the Time, 9=Don't Know, 98=Refused to Answer, -1=Missing Data
Note: "National Assembly Representative" in Benin, Madagascar, Mali, Nigeria, Senegal and South Africa; "Member of Parliament" in Botswana, Cape Verde, Ghana, Kenya, Lesotho, Malawi, Mozambique, Tanzania, Uganda, Zambia, and Zimbabwe. In Namibia, the question read: "How much time should representatives to the National Assembly spend visiting communities and citizens?"

Question Number: Q61
Question: How much time does your Member of Parliament/National Assembly Representative spend in this constituency?
Variable Label: Time MP does spend
Values: 0-4, 9, 98, -1
Value Labels: 0=Never 1=At Least Once a Year, 2=At Least Once a Month , 3=At Least Weekly, 4=Here Almost All of the Time, 9=Don't Know, 98=Refused to Answer, -1=Missing Data
Note: "National Assembly Representative" in Benin, Madagascar, Mali, Nigeria, Senegal and South Africa; "Member of Parliament" in Botswana, Cape Verde, Ghana, Kenya, Lesotho, Malawi, Mozambique, Tanzania, Uganda, Zambia, and Zimbabwe. In Namibia, the question read: "How much time do representatives to the National Assembly spend visiting communities and citizens?"

Question Number: Q62A
Question: How much of the time do think the following try their best to listen to what people like you have to say: Members of Parliament/National Assembly Representatives?

Variable Label: MPs listen
Values: 0-3, 9, 98, -1
Value Labels: 0=Never 1=Only Sometimes, 2=Often, 3=Always, 9=Don't Know, 98=Refused to Answer, -1=Missing Data
Note: "National Assembly Representatives" in Benin, Madagascar, Mali, Namibia, Nigeria, Senegal and South Africa; "Members of Parliament" in Botswana, Cape Verde, Ghana, Kenya, Lesotho, Malawi, Mozambique, Tanzania, Uganda, Zambia, and Zimbabwe.

Question Number: Q62B
Question: How much of the time do think the following try their best to listen to what people like you have to say: Elected Local Government Councilors?
Variable Label: Local government councilors listen
Values: 0-3, 9, 98, -1
Value Labels: 0=Never 1=Only Sometimes, 2=Often, 3=Always, 9=Don't Know, 98=Refused to Answer, -1=Missing Data
Note: "Elected Local Government Councilors" in Benin, Botswana, Kenya, Madagascar, Malawi, Mali, Mozambique, Namibia, Nigeria, Senegal, South Africa, Tanzania, Uganda, Zambia, and Zimbabwe; "Assembly Man/Woman" in Ghana. Elected Village Development Councilors in Lesotho. Vereadores in Cape Verde.

Question Number: Q63PT1
Question: In your opinion, what are the most important problems facing this country that government should address?
Variable Label: Most important problems – 1st response
Values: 0-31, 340, 995, 998-999, -1
Value Labels: 0=Nothing/No problems, 1=Management of the economy,2=Wages, income and salaries, 3=Unemployment, 4=Poverty/destitution, 5=Rates and taxes, 6=Loans /credit , 7=Farming/agriculture, 8=Food shortage/famine, 9=Drought, 10=Land, 11=Transportation, 12=Communications, 13=Infrastructure/roads, 14=Education, 15=Housing, 16=Electricity, 17=Water supply, 18=Orphans/street children/homeless children, 19= Services/other, 20=Health, 21=AIDS, 22=Sickness/Disease, 23=Crime and Security, 24=Corruption, 25=Political Violence, 26=Political instability/ethnic tensions, 27=Discrimination/inequality, 28=Gender issues/women's rights, 29=Democracy/political rights, 30=War(international), 31=Civil War, 340=Increase of fuel price, 995=Other, 998=Refused to answer, 999=Don't know, -1=Missing data
Source: SAB
Note: Respondent could give up to three responses.

Question Number: Q63PT2
Question: In your opinion, what are the most important problems facing this country that government should address?
Variable Label: Most important problems – 2nd response
Values: 1-31, 340, 995, 996, -1
Value Labels: 1=Management of the economy,2=Wages, income and salaries, 3=Unemployment, 4=Poverty/destitution, 5=Rates and taxes, 6=Loans /credit , 7=Farming/agriculture, 8=Food shortage/famine, 9=Drought, 10=Land, 11=Transportation, 12=Communications, 13=Infrastructure/roads, 14=Education, 15=Housing, 16=Electricity, 17=Water supply, 18=Orphans/street children/homeless children, 19= Services/other, 20=Health, 21=AIDS, 22=Sickness/Disease, 23=Crime and Security, 24=Corruption, 25=Political Violence, 26=Political instability/ethnic tensions, 27=Discrimination/inequality, 28=Gender issues/women's rights, 29=Democracy/political rights, 30=War(international), 31=Civil War, 340=Increase of fuel price, 995=Other, 996=No further response, -1=Missing data
Source: SAB
Note: Respondent could give up to three responses.

Question Number: Q63PT3
Question: In your opinion, what are the most important problems facing this country that government should address?
Variable Label: Most important problems – 3rd response
Values: 1-31, 340, 995, 996, -1
Value Labels: 1=Management of the economy,2=Wages, income and salaries, 3=Unemployment, 4=Poverty/destitution, 5=Rates and taxes, 6=Loans /credit , 7=Farming/agriculture, 8=Food shortage/famine,

9=Drought, 10=Land, 11=Transportation, 12=Communications, 13=Infrastructure/roads, 14=Education, 15=Housing, 16=Electricity, 17=Water supply, 18=Orphans/street children/homeless children, 19= Services/other, 20=Health, 21=AIDS, 22=Sickness/Disease, 23=Crime and Security, 24=Corruption, 25=Political Violence, 26=Political instability/ethnic tensions, 27=Discrimination/inequality, 28=Gender issues/women's rights, 29=Democracy/political rights, 30=War(international), 31=Civil War, 340=Increase of fuel price, 995=Other, 996=No further response, -1=Missing data
Source: SAB
Note: Respondent could give up to three responses.

Question Number: Q64
Question: Taking the Problem you mentioned first, how likely is it that the government will solve your most important problem within the next few years?
Variable Label: Government solve MIP
Values: 0-3, 7, 9, 98, -1
Value Labels: 0= Not At All Likely, 1=Not Very Likely, 2=Likely, 3=Very Likely, 7=Not Applicable, 9=Don't Know/Haven't heard enough, 98=Refused to Answer, -1=Missing Data
Source: SAB
Note: Interviewer probed for strength of opinion.

Question Number: Q65A
Question: Now let's speak about the present government of this country. How well or badly would you say the current government is handling the following matters, or haven't you heard enough about them to say: Managing the economy?
Variable Label: Handling managing the economy
Values: 1-4, 9, 98, -1
Value Labels: 1=Very Badly, 2=Fairly Badly, 3=Fairly Well, 4=Very Well, 9=Don't Know/Haven't heard enough, 98=Refused to Answer, -1=Missing Data
Source: SAB
Note: Interviewer probed for strength of opinion.

Question Number: Q65B
Question: How well or badly would you say the current government is handling the following matters, or haven't you heard enough about them to say: Creating jobs?
Variable Label: Handling creating jobs
Values: 1-4, 9, 98, -1
Value Labels: 1=Very Badly, 2=Fairly Badly, 3=Fairly Well, 4=Very Well, 9=Don't Know/Haven't heard enough, 98=Refused to Answer, -1=Missing Data
Source: NDB
Note: Interviewer probed for strength of opinion.

Question Number: Q65C
Question: How well or badly would you say the current government is handling the following matters, or haven't you heard enough about them to say: Keeping prices stable?
Variable Label: Handling keeping prices stable
Values: 1-4, 9, 98, -1
Value Labels: 1=Very Badly, 2=Fairly Badly, 3=Fairly Well, 4=Very Well, 9=Don't Know/Haven't heard enough, 98=Refused to Answer, -1=Missing Data
Source: NDB
Note: Interviewer probed for strength of opinion.

Question Number: Q65D
Question: How well or badly would you say the current government is handling the following matters, or haven't you heard enough about them to say: Narrowing gaps between rich and poor?
Variable Label: Handling narrowing income gaps
Values: 1-4, 9, 98, -1
Value Labels: 1=Very Badly, 2=Fairly Badly, 3=Fairly Well, 4=Very Well, 9=Don't Know/Haven't heard enough, 98=Refused to Answer, -1=Missing Data
Source: SAB

Note: Interviewer probed for strength of opinion.

Question Number: Q65E
Question: How well or badly would you say the current government is handling the following matters, or haven't you heard enough about them to say: Reducing crime?
Variable Label: Handling reducing crime
Values: 1-4, 9, 98, -1
Value Labels: 1=Very Badly, 2=Fairly Badly, 3=Fairly Well, 4=Very Well, 9=Don't Know/Haven't heard enough, 98=Refused to Answer, -1=Missing Data
Source: NDB
Note: Interviewer probed for strength of opinion.

Question Number: Q65F
Question: How well or badly would you say the current government is handling the following matters, or haven't you heard enough about them to say: Improving basic health services?
Variable Label: Handling improving basic health services
Values: 1-4, 9, 98, -1
Value Labels: 1=Very Badly, 2=Fairly Badly, 3=Fairly Well, 4=Very Well, 9=Don't Know/Haven't heard enough, 98=Refused to Answer, -1=Missing Data
Source: NDB
Note: Interviewer probed for strength of opinion.

Question Number: Q65G
Question: How well or badly would you say the current government is handling the following matters, or haven't you heard enough about them to say: Addressing educational needs?
Variable Label: Handling addressing educational needs
Values: 1-4, 9, 98, -1
Value Labels: 1=Very Badly, 2=Fairly Badly, 3=Fairly Well, 4=Very Well, 9=Don't Know/Haven't heard enough, 98=Refused to Answer, -1=Missing Data
Source: NDB
Note: Interviewer probed for strength of opinion.

Question Number: Q65H
Question: How well or badly would you say the current government is handling the following matters, or haven't you heard enough about them to say: Delivering household water?
Variable Label: Handling delivering household water
Values: 1-4, 9, 98, -1
Value Labels: 1=Very Badly, 2=Fairly Badly, 3=Fairly Well, 4=Very Well, 9=Don't Know/Haven't heard enough, 98=Refused to Answer, -1=Missing Data
Source: SAB
Note: Interviewer probed for strength of opinion.

Question Number: Q65I
Question: How well or badly would you say the current government is handling the following matters, or haven't you heard enough about them to say: Ensuring everyone has enough to eat?
Variable Label: Handling ensuring enough to eat
Values: 1-4, 9, 98, -1
Value Labels: 1=Very Badly, 2=Fairly Badly, 3=Fairly Well, 4=Very Well, 9=Don't Know/Haven't heard enough, 98=Refused to Answer, -1=Missing Data
Source: SAB
Note: Interviewer probed for strength of opinion.

Question Number: Q65J
Question: How well or badly would you say the current government is handling the following matters, or haven't you heard enough about them to say: Fighting corruption in government?
Variable Label: Handling fighting corruption
Values: 1-4, 9, 98, -1

Value Labels: 1=Very Badly, 2=Fairly Badly, 3=Fairly Well, 4=Very Well, 9=Don't Know/Haven't heard enough, 98=Refused to Answer, -1=Missing Data
Source: SAB
Note: Interviewer probed for strength of opinion.

Question Number: Q65K
Question: How well or badly would you say the current government is handling the following matters, or haven't you heard enough about them to say: Combating HIV/AIDS?
Variable Label: Handling combating HIV/AIDS
Values: 1-4, 9, 98, -1
Value Labels: 1=Very Badly, 2=Fairly Badly, 3=Fairly Well, 4=Very Well, 9=Don't Know/Haven't heard enough, 98=Refused to Answer, -1=Missing Data
Source: SAB
Note: Interviewer probed for strength of opinion.

Question Number: Q66
Question: Which of these statements is closest to your view? Choose Statement A or Statement B.
A: The government should devote many more resources to combating AIDS, even if this means that less money is spent on things like education.
B: There are many other problems facing this country beside AIDS; even if people are dying in large numbers, the government needs to keep its focus on solving other problems.
Variable Label: Resources to combat AIDS vs. other problems
Values: 1-5, 9, 98, -1
Value Labels: 1=Agree Very Strongly with A, 2=Agree with A, 3=Agree with B, 4=Agree Very Strongly with B, 5=Agree with Neither, 9=Don't Know, 98=Refused to Answer, -1=Missing Data
Source: Afrobarometer Round 2
Note: Interviewer probed for strength of opinion, asking "Do you agree or agree very strongly?"

Question Number: Q67A
Question: What about local government? How well or badly would you say your local government is handling the following matters, or haven't you heard enough about them to say: Maintaining our roads?
Variable Label: Local govt. handling maintaining roads
Values: 1-4, 9, 98, -1
Value Labels: 1=Very Badly, 2=Fairly Badly, 3=Fairly Well, 4=Very Well, 9=Don't Know, 98=Refused to Answer, -1=Missing Data
Source: Afrobarometer Round 3

Question Number: Q67B
Question: What about local government? How well or badly would you say your local government is handling the following matters, or haven't you heard enough about them to say: Keeping our community clean?
Variable Label: Local govt. handling keeping community clean
Values: 1-4, 9, 98, -1
Value Labels: 1=Very Badly, 2=Fairly Badly, 3=Fairly Well, 4=Very Well, 9=Don't Know, 98=Refused to Answer, -1=Missing Data
Source: Afrobarometer Round 3

Question Number: Q67C
Question: What about local government? How well or badly would you say your local government is handling the following matters, or haven't you heard enough about them to say: Collecting Local Taxes?
Variable Label: Local govt. handling collecting local taxes
Values: 1-4, 9, 98, -1
Value Labels: 1=Very Badly, 2=Fairly Badly, 3=Fairly Well, 4=Very Well, 9=Don't Know, 98=Refused to Answer, -1=Missing Data
Source: Afrobarometer Round 3

Question Number: Q67D
Question: What about local government? How well or badly would you say your local government is handling the following matters, or haven't you heard enough about them to say: Deciding how to spend local revenues?

Variable Label: Local govt. handling spending decisions
Values: 1-4, 9, 98, -1
Value Labels: 1=Very Badly, 2=Fairly Badly, 3=Fairly Well, 4=Very Well, 9=Don't Know, 98=Refused to Answer, -1=Missing Data
Source: Afrobarometer Round 3

Question Number: Q68A
Question: Do you approve or disapprove of the way the following people have performed their jobs over the past twelve months, or haven't you heard enough about them to say: [President/Prime Minister's name]?
Variable Label: Performance: President
Values: 1-4, 9, 98, -1
Value Labels: 1=Strongly Disapprove, 2=Disapprove, 3=Approve, 4=Strongly Approve, 9=Don't Know/Haven't heard enough, 98=Refused to Answer, -1=Missing Data
Source: SAB
Note: Interviewer probed for strength of opinion. President Kerekou in Benin; President Mogae in Botswana; President Pedro Pires in Cape Verde; President Kufuor in Ghana; President Kibaki in Kenya; Prime Minister Mosisili in Lesotho; President Ravalomanana in Madagascar; President Mutharika in Malawi; President Touré in Mali; President Guebuza in Mozambique; President Pohamba in Namibia; President Obasanjo in Nigeria; President Wade in Senegal; President Mbeki in South Africa; President Mkapa in Tanzania; President Museveni in Uganda; President Mwanawasa in Zambia; and President Mugabe in Zimbabwe.

Question Number: Q68B
Question: Do you approve or disapprove of the way the following people have performed their jobs over the past twelve months, or haven't you heard enough about them to say: Your Members of Parliament/National Assembly Representative?
Variable Label: Performance: MP/National Assembly Rep.
Values: 1-4, 9, 98, -1
Value Labels: 1=Strongly Disapprove, 2=Disapprove, 3=Approve, 4=Strongly Approve, 9=Don't Know/Haven't heard enough, 98=Refused to Answer, -1=Missing Data
Source: SAB
Note: Interviewer probed for strength of opinion. "Your National Assembly Representative" in Nigeria and Senegal; "National Assembly Representatives" in Benin, Cape Verde, Madagascar, Mali, Mozambique, Namibia and South Africa; "Your Member of Parliament" Kenya, Tanzania, Zambia and Zimbabwe; "The Members of Parliament" in Botswana, Ghana, Lesotho, Malawi and Uganda.

Question Number: Q68C
Question: Do you approve or disapprove of the way the following people have performed their jobs over the past twelve months, or haven't you heard enough about them to say: Your Assembly Man/Woman/Local Government Councilor?
Variable Label: Performance: local government councilor
Values: 1-4, 9, 98, -1
Value Labels: 1=Strongly Disapprove, 2=Disapprove, 3=Approve, 4=Strongly Approve, 9=Don't Know/Haven't heard enough, 98=Refused to Answer, -1=Missing Data
Source: SAB
Note: Interviewer probed for strength of opinion. "Your Elected Local Government Councilor" in Benin, Botswana, Kenya, Malawi, Mali, Namibia, Nigeria, Senegal, South Africa, Tanzania, Uganda, Zambia, and Zimbabwe; "Elected local government councilors" in Mozambique; "Your Assembly Man/Woman" in Ghana; "Your elected Village Development Councilor" in Lesotho; "Os Vereadores" in Cape Verde; "Les maires et leurs conseillers elus" in Madagascar.

Question Number: Q69A
Question: Can you tell me whether the [Ghanaian/Kenyan/etc.] government has a policy to provide: Free primary education, that is, parents do not have to pay school fees?
Variable Label: Policy knowledge: Free primary education.
Values: 1-2, 9, 98, -1
Value Labels: 1=Incorrect answer, 2=Correct Answer, 9=Don't Know, 98=Refused to Answer, -1=Missing Data
Source: Afrobarometer Round 3
Note: Question not asked in Zimbabwe. Correct answer is no in Mali and Senegal, yes in all other countries.

Question Number: Q69B
Question: Can you tell me whether the [Ghanaian/Kenyan/etc.] government has a policy to provide: Free health care at public clinics, that is, no fees for visits or medicine?
Variable Label: Policy knowledge: Free health care
Values: 1-2, 9, 98, -1
Value Labels: 1=Incorrect answer, 2=Correct Answer, 9=Don't Know, 98=Refused to Answer, -1=Missing Data
Source: Afrobarometer Round 3
Note: Question not asked in Zimbabwe. Correct answer is no in Benin, Botswana, Ghana, Kenya, Lesotho, Madagascar, Mali, Mozambique, Senegal, Tanzania, Uganda and Zambia and yes in Cape Verde, Malawi, Namibia, Nigeria, and South Africa.

Question Number: Q70A
Question: How likely do you think it would be that the authorities could enforce the law if a top government official committed a serious crime?
Variable Label: Enforce law: Top official commits serious crime
Values: 1-4, 9, 98, -1
Value Labels: 1=Not at all likely, 2=Not very likely, 3=Likely, 4=Very Likely, 9=Don't Know, 98=Refused to Answer, -1=Missing Data
Source: Afrobarometer Round 3

Question Number: Q70B
Question: How likely do you think it would be that the authorities could enforce the law if a person like you committed a serious crime?
Variable Label: Enforce law: You commit serious crime
Values: 1-4, 9, 98, -1
Value Labels: 1=Not at all likely, 2=Not very likely, 3=Likely, 4=Very Likely, 9=Don't Know, 98=Refused to Answer, -1=Missing Data
Source: Afrobarometer Round 1

Question Number: Q70C
Question: How likely do you think it would be that the authorities could enforce the law if a top official did not pay a tax on some of the income they earned?
Variable Label: Enforce law: Top official doesn't pay tax
Values: 1-4, 9, 98, -1
Value Labels: 1=Not at all likely, 2=Not very likely, 3=Likely, 4=Very Likely, 9=Don't Know, 98=Refused to Answer, -1=Missing Data
Source: Afrobarometer Round 3

Question Number: Q70D
Question: How likely do you think it would be that the authorities could enforce the law if a person like you did not pay a tax on some of the income you earned?
Variable Label: Enforce law: You don't pay tax
Values: 1-4, 9, 98, -1
Value Labels: 1=Not at all likely, 2=Not very likely, 3=Likely, 4=Very Likely, 9=Don't Know, 98=Refused to Answer, -1=Missing Data
Source: Afrobarometer Round 2

Question Number: Q71A
Question: Based on your experience, how easy or difficult is to obtain the following services? Or do you never try and get these services from government: An identity document (such as a birth certificate, driver's license, passport or voter card)?
Variable Label: Difficulty to obtain identity document
Values: 1-4, 7, 9, 98, -1
Value Labels: 1=Very Difficult, 2=Difficult, 3=Easy, 4=Very Easy, 7=Never Try, 9=Don't Know, 98=Refused to Answer, -1=Missing Data
Source: Adapted from Afrobarometer Round 1

Question Number: Q71B
Question: Based on your experience, how easy or difficult is to obtain the following services? Or do you never try and get these services from government: Household services (like piped water, electricity or telephone)?
Variable Label: Difficulty to obtain household services
Values: 1-4, 7, 9, 98, -1
Value Labels: 1=Very Difficult, 2=Difficult, 3=Easy, 4=Very Easy, 7=Never Try, 9=Don't Know, 98=Refused to Answer, -1=Missing Data
Source: Adapted from SAB

Question Number: Q71C
Question: Based on your experience, how easy or difficult is to obtain the following services? Or do you never try and get these services from government: Help from the police when you need it?
Variable Label: Difficulty to obtain help from the police
Values: 1-4, 7, 9, 98, -1
Value Labels: 1=Very Difficult, 2=Difficult, 3=Easy, 4=Very Easy, 7=Never Try, 9=Don't Know, 98=Refused to Answer, -1=Missing Data
Source: Afrobarometer Round 2

Question Number: Q71D
Question: Based on your experience, how easy or difficult is to obtain the following services? Or do you never try and get these services from government: A place in primary school for a child?
Variable Label: Difficulty to obtain primary school placement
Values: 1-4, 7, 9, 98, -1
Value Labels: 1=Very Difficult, 2=Difficult, 3=Easy, 4=Very Easy, 7=Never Try, 9=Don't Know, 98=Refused to Answer, -1=Missing Data
Source: SAB

Question Number: Q71E
Question: Based on your experience, how easy or difficult is to obtain the following services? Or do you never try and get these services from government: Medical treatment at a nearby clinic?
Variable Label: Difficulty to obtain medical treatment
Values: 1-4, 7, 9, 98, -1
Value Labels: 1=Very Difficult, 2=Difficult, 3=Easy, 4=Very Easy, 7=Never Try, 9=Don't Know, 98=Refused to Answer, -1=Missing Data
Source: SAB

Question Number: Q72A
Question: Why do you never try to get: An identity document?
Variable Label: Why not try to get: identity document
Values: 1-5, 7, 9, 98, -1
Value Labels: 1=I don't need it/use it, 2=I don't know how, 3=Govt does not provide it, 4=No personal connections in right places, 5=Some other response, 7=N/A, 9=Don't Know, 98=Refused to Answer, -1=Missing Data
Source: Afrobarometer Round 3
Note: Interviewer was instructed as follows "Ask only if response to Q71A was 7=Never try. If any other response was given to Q71A, select 7=Not applicable."

Question Number: Q72B
Question: Why do you never try to get: Household services?
Variable Label: Why not try to get: household services.
Values: 1-5, 7, 9, 98, -1
Value Labels: 1=I don't need it/use it, 2=I don't know how, 3=Govt does not provide it, 4=No personal connections in right places, 5=Some other response, 7=N/A, 9=Don't Know, 98=Refused to Answer, -1=Missing Data
Source: Afrobarometer Round 3
Note: Interviewer was instructed as follows "Ask only if response to Q71B was 7=Never try. If any other response was given to Q71B, select 7=Not applicable."

Copyright Afrobarometer

Question Number: Q72C
Question: Why do you never try to get: Help from the police when you need it?
Variable Label: Why not try to get: Help from police
Values: 1-5, 7, 9, 98, -1
Value Labels: 1=I don't need it/use it, 2=I don't know how, 3=Govt does not provide it, 4=No personal connections in right places, 5=Some other response, 7=N/A, 9=Don't Know, 98=Refused to Answer, -1=Missing Data
Source: Afrobarometer Round 3
Note: Interviewer was instructed as follows "Ask only if response to Q71C was 7=Never try. If any other response was given to Q71C, select 7=Not applicable."

Question Number: Q73A
Question: Have you encountered any of these problems with your local public schools during the past 12 months: Services are too expensive / Unable to pay?
Variable Label: Problems with schools: too expensive.
Values: 0-3, 7, 9, 98, -1
Value Labels: 0=Never, 1=Once or twice, 2=A few times, 3=Often, 7=No experience with public schools in the past twelve months, 9=Don't Know, 98=Refused to Answer, -1=Missing Data
Source: Afrobarometer Round 3

Question Number: Q73B
Question: Have you encountered any of these problems with your local public schools during the past 12 months: Lack of textbooks or other supplies?
Variable Label: Problems with schools: textbooks and supplies.
Values: 0-3, 7, 9, 98, -1
Value Labels: 0=Never, 1=Once or twice, 2=A few times, 3=Often, 7=No experience with public schools in the past twelve months, 9=Don't Know, 98=Refused to Answer, -1=Missing Data
Source: Afrobarometer Round 3

Question Number: Q73C
Question: Have you encountered any of these problems with your local public schools during the past 12 months: Poor teaching?
Variable Label: Problems with schools: poor teaching.
Values: 0-3, 7, 9, 98, -1
Value Labels: 0=Never, 1=Once or twice, 2=A few times, 3=Often, 7=No experience with public schools in the past twelve months, 9=Don't Know, 98=Refused to Answer, -1=Missing Data
Source: Afrobarometer Round 3

Question Number: Q73D
Question: Have you encountered any of these problems with your local public schools during the past 12 months: Absent teachers?
Variable Label: Problems with schools: Absent teachers.
Values: 0-3, 7, 9, 98, -1
Value Labels: 0=Never, 1=Once or twice, 2=A few times, 3=Often, 7=No experience with public schools in the past twelve months, 9=Don't Know, 98=Refused to Answer, -1=Missing Data
Source: Afrobarometer Round 3

Question Number: Q73E
Question: Have you encountered any of these problems with your local public schools during the past 12 months: Overcrowded classrooms?
Variable Label: Problems with schools: overcrowded classes.
Values: 0-3, 7, 9, 98, -1
Value Labels: 0=Never, 1=Once or twice, 2=A few times, 3=Often, 7=No experience with public schools in the past twelve months, 9=Don't Know, 98=Refused to Answer, -1=Missing Data
Source: Afrobarometer Round 3

Question Number: Q73F
Question: Have you encountered any of these problems with your local public schools during the past 12 months: Poor conditions of facilities?

Copyright Afrobarometer

Variable Label: Problems with schools: poor facilities.
Values: 0-3, 7, 9, 98, -1
Value Labels: 0=Never, 1=Once or twice, 2=A few times, 3=Often, 7=No experience with public schools in the past twelve months, 9=Don't Know, 98=Refused to Answer, -1=Missing Data
Source: Afrobarometer Round 3

Question Number: Q73G
Question: Have you encountered any of these problems with your local public schools during the past 12 months: Demands for illegal payments?
Variable Label: Problems with schools: illegal payments.
Values: 0-3, 7, 9, 98, -1
Value Labels: 0=Never, 1=Once or twice, 2=A few times, 3=Often, 7=No experience with public schools in the past twelve months, 9=Don't Know, 98=Refused to Answer, -1=Missing Data
Source: Afrobarometer Round 3

Question Number: Q74A
Question: Have you encountered any of these problems with your local public clinic or hospital during the past 12 months: Services are too expensive / Unable to pay?
Variable Label: Problems with public clinic: too expensive.
Values: 0-3, 7, 9, 98, -1
Value Labels: 0=Never, 1=Once or twice, 2=A few times, 3=Often, 7=No experience with clinics in the past twelve months, 9=Don't Know, 98=Refused to Answer, -1=Missing Data
Source: Afrobarometer Round 3

Question Number: Q74B
Question: Have you encountered any of these problems with your local public clinic or hospital during the past 12 months: Lack of medicines or other supplies?
Variable Label: Problems with public clinic: Lack of medicines/supplies.
Values: 0-3, 7, 9, 98, -1
Value Labels: 0=Never, 1=Once or twice, 2=A few times, 3=Often, 7=No experience with clinics in the past twelve months, 9=Don't Know, 98=Refused to Answer, -1=Missing Data
Source: Afrobarometer Round 3

Question Number: Q74C
Question: Have you encountered any of these problems with your local public clinic or hospital during the past 12 months: Lack of attention or respect from staff?
Variable Label: Problems with public clinic: Lack of attention/respect.
Values: 0-3, 7, 9, 98, -1
Value Labels: 0=Never, 1=Once or twice, 2=A few times, 3=Often, 7=No experience with clinics in the past twelve months, 9=Don't Know, 98=Refused to Answer, -1=Missing Data
Source: Afrobarometer Round 3

Question Number: Q74D
Question: Have you encountered any of these problems with your local public clinic or hospital during the past 12 months: Absent doctors?
Variable Label: Problems with public clinic: Absent doctors.
Values: 0-3, 7, 9, 98, -1
Value Labels: 0=Never, 1=Once or twice, 2=A few times, 3=Often, 7=No experience with clinics in the past twelve months, 9=Don't Know, 98=Refused to Answer, -1=Missing Data
Source: Afrobarometer Round 3

Question Number: Q74E
Question: Have you encountered any of these problems with your local public clinic or hospital during the past 12 months: Long waiting time?
Variable Label: Problems with public clinic: Long waiting time.
Values: 0-3, 7, 9, 98, -1
Value Labels: 0=Never, 1=Once or twice, 2=A few times, 3=Often, 7=No experience with clinics in the past twelve months, 9=Don't Know, 98=Refused to Answer, -1=Missing Data

Copyright Afrobarometer

Source: Afrobarometer Round 3

Question Number: Q74F
Question: Have you encountered any of these problems with your local public clinic or hospital during the past 12 months: Dirty facilities?
Variable Label: Problems with public clinic: Dirty facilities.
Values: 0-3, 7, 9, 98, -1
Value Labels: 0=Never, 1=Once or twice, 2=A few times, 3=Often, 7=No experience with clinics in the past twelve months, 9=Don't Know, 98=Refused to Answer, -1=Missing Data
Source: Afrobarometer Round 3

Question Number: Q74G
Question: Have you encountered any of these problems with your local public clinic or hospital during the past 12 months: Demands for illegal payments?
Variable Label: Problems with public clinic: illegal payments.
Values: 0-3, 7, 9, 98, -1
Value Labels: 0=Never, 1=Once or twice, 2=A few times, 3=Often, 7=No experience with clinics in the past twelve months, 9=Don't Know, 98=Refused to Answer, -1=Missing Data
Source: Afrobarometer Round 3

Question Number: Q75A
Question: What, if anything, would you do to try and resolve each of the following situations: You were waiting for a government permit or license, but kept encountering delays?
Variable Label: What do: Waiting for permit.
Values: 1-7, 9, 98, -1
Value Labels: 1= Don't worry, things will be resolved given enough time, 2= Lodge complaint through proper channels or procedures, 3= Use connections with influential people, 4= Offer tip or bribe, 5= Join in Public Protest, 6=Other, 7=Nothing, because nothing can be done, 9=Don't Know, 98=Refused to Answer, -1=Missing Data
Source: Afrobarometer Round 3

Question Number: Q75B
Question: What, if anything, would you do to try and resolve each of the following situations: Election officials left your name off the voters roll?
Variable Label: What do: Name missing.
Values: 1-7, 9, 98, -1
Value Labels: 1= Don't worry, things will be resolved given enough time, 2= Lodge complaint through proper channels or procedures, 3= Use connections with influential people, 3= Use connections with influential people, 4= Offer tip or bribe, 5= Join in Public Protest, 6=Other, 7=Nothing, because nothing can be done, 9=Don't Know, 98=Refused to Answer, -1=Missing Data
Source: Afrobarometer Round 3

Question Number: Q75C
Question: What, if anything, would you do to try and resolve each of the following situations: You suspected a school or clinic official of stealing?
Variable Label: What do: Suspect school official.
Values: 1-7, 9, 98, -1
Value Labels: 1= Don't worry, things will be resolved given enough time, 2= Lodge complaint through proper channels or procedures, 3= Use connections with influential people, 3= Use connections with influential people, 4= Offer tip or bribe, 5= Join in Public Protest, 6=Other, 7=Nothing, because nothing can be done, 9=Don't Know, 98=Refused to Answer, -1=Missing Data
Source: Afrobarometer Round 3

Question Number: Q75D
Question: What, if anything, would you do to try and resolve each of the following situations: The police wrongly arrested someone in your family?
Variable Label: What do: Wrongful arrest.
Values: 1-7, 9, 98, -1

Copyright Afrobarometer

Value Labels: 1= Don't worry, things will be resolved given enough time, 2= Lodge complaint through proper channels or procedures, 3= Use connections with influential people, 3= Use connections with influential people, 4= Offer tip or bribe, 5= Join in Public Protest, 6=Other, 7=Nothing, because nothing can be done, 9=Don't Know, 98=Refused to Answer, -1=Missing Data
Source: Afrobarometer Round 3

Question Number: Q75E
Question: What, if anything, would you do to try and resolve each of the following situations: Someone wrongly seized your family's land?
Variable Label: What do: Land seized.
Values: 1-7, 9, 98, -1
Value Labels: 1= Don't worry, things will be resolved given enough time, 2= Lodge complaint through proper channels or procedures, 3= Use connections with influential people, 3= Use connections with influential people, 4= Offer tip or bribe, 5= Join in Public Protest, 6=Other, 7=Nothing, because nothing can be done, 9=Don't Know, 98=Refused to Answer, -1=Missing Data
Source: Afrobarometer Round 3

Question Number: Q76A
Question: Who should be responsible for making sure that, once elected, Members of Parliament/National Assembly Representatives do their jobs?
Variable Label: Who responsible: MPs do jobs
Values: 0-3, 9, 98, -1
Value Labels: 0= The President/Executive, 1= The Parliament, 2= Their Political Party, 3= The Voters, 9=Don't Know, 98=Refused to Answer, -1=Missing Data
Source: Afrobarometer Round 3
Note: "National Assembly Representatives" in Benin, Madagascar, Mali, Mozambique, Namibia, Nigeria, Senegal and South Africa; "Members of Parliament" in Botswana, Cape Verde, Ghana, Kenya, Lesotho, Malawi, Tanzania, Uganda, Zambia, and Zimbabwe.

Question Number: Q76B
Question: Who should be responsible for making sure that, once elected, Local Government Councilors do their jobs?
Variable Label: Who responsible: Local councilors do jobs
Values: 0-3, 9, 98, -1
Value Labels: 0= The President/Executive, 1= The Local Council, 2= Their Political Party, 3= The Voters, 9=Don't Know, 98=Refused to Answer, -1=Missing Data
Source: Afrobarometer Round 3
Note: "Local Government Councilors" in Benin, Botswana, Kenya, Madagascar, Malawi, Mali, Mozambique, Namibia, Nigeria, Senegal, South Africa, Tanzania, Uganda, Zambia, and Zimbabwe; "Assembly Man/Woman" in Ghana; "Elected Village Development Councilors" in Lesotho; "Vereadores" in Cape Verde.

Question Number: Q77A
Question: Think about how elections work in practice in this country. How well do elections ensure that the Members of Parliament/Nation Assembly Representatives reflect the views of voters?
Variable Label: Elections ensure parliament reflects voters.
Values: 0-3, 9, 98, -1
Value Labels: 0= Not at all well, 1= Not very well, 2= Well, 3= Very Well, 9=Don't Know, 98=Refused to Answer, -1=Missing Data
Source: Afrobarometer Round 3
Note: "National Assembly Representatives" in Benin, Madagascar, Mali, Mozambique, Namibia, Nigeria, Senegal and South Africa; "Members of Parliament" in Botswana, Cape Verde, Ghana, Kenya, Lesotho, Malawi, Tanzania, Uganda, Zambia, and Zimbabwe.

Question Number: Q77B
Question: Think about how elections work in practice in this country. How well do elections enable voters to remove from office leaders who do not do what the people want?
Variable Label: Elections enable voters to remove leaders.
Values: 0-3, 9, 98, -1

Value Labels: 0= Not at all well, 1= Not very well, 2= Well, 3= Very Well, 9=Don't Know, 98=Refused to Answer, -1=Missing Data
Source: Afrobarometer Round 3

Question Number: Q78A
Question: In your opinion, how often do politicians do each of the following: Make promises simply to get elected?
Variable Label: Politicians make promises to get elected
Values: 0-3, 9, 98, -1
Value Labels: 0=Never, 1=Rarely, 2=Often, 3=Always, 9=Don't Know, 98=Refused to Answer, -1=Missing Data
Source: Afrobarometer Round 3

Question Number: Q78B
Question: In your opinion, how often do politicians do each of the following: Offer gifts to voters during election campaigns?
Variable Label: Politicians give gifts in campaigns.
Values: 0-3, 9, 98, -1
Value Labels: 0=Never, 1=Rarely, 2=Often, 3=Always, 9=Don't Know, 98=Refused to Answer, -1=Missing Data
Source: Afrobarometer Round 3

Question Number: Q78C
Question: In your opinion, how often do politicians do each of the following: Keep their campaign promises after elections?
Variable Label: Politicians keep campaign promises
Values: 0-3, 9, 98, -1
Value Labels: 0=Never, 1=Rarely, 2=Often, 3=Always, 9=Don't Know, 98=Refused to Answer, -1=Missing Data
Source: Afrobarometer Round 3

Question Number: Q78D
Question: In your opinion, how often do politicians do each of the following: Do their best to deliver development after elections?
Variable Label: Politicians deliver development.
Values: 0-3, 9, 98, -1
Value Labels: 0=Never, 1=Rarely, 2=Often, 3=Always, 9=Don't Know, 98=Refused to Answer, -1=Missing Data
Source: Afrobarometer Round 3

Question Number: Q79
Question: What is your tribe? You know, your ethnic or cultural group.
Variable Label: Tribe or ethnic group
Values: 1-3, 100-113, 120-130, 140-160, 169-178, 180-185, 200-246, 248-258, 260-271, 280-298, 300-309, 320-342, 344-358, 360-369, 380-398, 405-420, 422-433, 435-439, 500-511, 517, 550-551, 553-556, 558-568, 570-573, 575, 800-835, 860-875, 990, 995, 997, 998, 999, -1
Value Labels: 1=English, 2=Francais, 3=Portugais, 100=Afrikaans/Afrikaner/Boer, 101=Ndebele, 102=Xhosa, 103=Pedi/Spedi/North Sotho, 104=Sesotho/Sotho/South Sotho, 105=Setswana/Tswana, 106=Shangaan, 107=Swazi, 108=Venda, 109=Zulu, 110=Other White/European, 111=Couloured, 112=Indian, 113=English, 120=Fon, 121=Adja, 122=Bariba, 123=Dendi , 124=Yoruba, 125=Ditamari, 126=Peulh, 127=Yoa, 128=Haoussa, 129=Idé, 130=Lamba, 140=Mokgatla, 141=Mokwena, 142=Mongwato, 143=Mongwaketse, 144=Motlokwa, 145=Moherero, 146=Morolong, 147=Mosarwa, 148=Mokalanga, 149=Molete, 150=Mmirwa, 151=Motswapong, 152=Mosobea, 153=Motawana, 154=Mokgalagadi, 155=Moyei, 156=Mohurutshe, 157=Mombukushu, 158=Monajwa, 159=Mokhurutshe, 160=Motalaote, 169=Related to island origin, 170=African, 171=American or European, 172=Related to age, 173=Related to gender, 174=Related to occupation/profession, 175=Related to religion, 176=Related to class, 177=related to race, 178=Related to politics/partisan, 180=Akan, 181=Ewe, 182=Ga/Damgbe, 183= Other northern languages, 184=Gagomba, 185=Dagaati, 200=Kikuyu, 201=Luo, 202=Luhya, 203=Kamba, 204=Meru, 205=Kisii, 206=Kalenjin, 207=Masaai, 208=MijiKenda, 209=Taita, 210=Somali, 211=Pokot, 212=Turkana, 213=Bajuni, 214=Kuria, 215=Teso, 216=Rendille, 217=Embu, 218=Borana, 219=Samburu,

220=Bakoena, 221=Basiea, 222=Bafokeng, 223=Bahlakoana, 224=Batsoeneng, 225=Batloung, 226=Bataung, 227=Baphuthing, 228=Batebele, 229=Bathepu, 230=Batlokoa, 231=Bakhatla, 232=Makholokoe, 233=Makhoakhoa, 234=Barolong, 235=Banareng, 236=Mapele, 237=Bakubung, 238=Maswati, 239=Mchegu, 240=Antakarana, 241=Antambahoaka, 242=Antandroy, 243=Antanosy, 244=Antefasy , 245=Antemoro , 246=Antesaka , 248=Bara, 249=Betsileo, 250=Betsimisaraka , 251=Bezanozano , 252=Mahafaly, 253=Merina, 254=Sakalava , 255=Sihanaka , 256=Tanala, 257=Tsimihety, 258=Vezo, 260=Tumbuka, 261=Nkhonde, 262=Lambya, 263=Chewa, 264=Yao, 265=Ngoni, 266=Lomwe, 267=Mang'anja, 268=Sena, 269=Sukwa, 270=Senga, 271=Tonga, 280=Bambara, 281=Peuhl/Fulfulde, 282=Senufo, 283=Mianka, 284=Mossi, 285=Sonink, 286=Malink, 287=Khasonk, 288=Dogon, 289=Bobo, 290=Bozo, 291=Arabe, 292=Maure, 293=Kakolo, 294=Samoko, 295=Sonrha, 296=Bella, 297=Tamasheq, 298=Dafing, 300=Makua, 301=Sena, 302=Ndau, 303=Nyanja, 304=Changana, 305=Chope, 306=Bitonga, 307=Makonde, 308=Chuabo, 309=Ajua, 320=Oshiwambo, 321=Herero, 322=Caprivian, 323=Rukwangali, 324=German, 325=Afrikaaner, 326=English, 327=Nama, 328=Damara, 329=Lozi, 330=Subia, 331=White, 332=Black, 333=Coloured, 334=Baster, 335=Setswana, 336=Angolan:Portuguese, 337=Angolan:Rumbundu, 338=Ndembele (Zimbabwe), 339=Kavango, 340=Hausa, 341=Igbo, 342=Yoruba, 344=Efik, 345=Ebira, 346=Fulani, 347=Isoko, 348=Ibibio, 349=Kanuri, 350=Tiv, 351=Nupe, 352=Ijaw, 353=Edo, 354=Igala, 355=Urhobo, 356=Idoma, 357=Itsekiri, 358=Ikwere, 360=Wolof, 361=Pular, 362=Serer, 363=Mandinka, 364=Soninke, 365=Diola, 366=Manjack, 367=Bambara, 368=Bainouk, 369=Bassari, 380=Mnyakyusa, 381=Mchaga, 382=Mhaya, 383=Mngoni, 384=Mkwere, 385=Mpare, 386=Mhehe, 387=Mmakonde, 388=Myamwezi, 389=Msukuma, 390=Mmasai, 391=Mmeru, 392=Mkurya, 393=Mgogo, 394=Mluguru, 395=Mfipa, 396=Mmanyema, 397=Mnyiramba, 398=Mnyaturu, 405=Akaramojonj, 406=Alur, 407=Ateso, 408=Japadhola, 409=Jonam, 410=Kakwa, 411=Kumam, 412=Kuksabin, 413=Luo, 414=Lugbara, 415=Mugwere, 416=Madi, 417=Muganda, 418=Mugishu, 419=Musoga, 420=Bemba, 422=Tonga, 423=Lozi, 424=Chewa, 425=Nsenga, 426=Tumbuka, 427=Kaonde, 428=Luvale, 429=Namwanga, 430=Mambwe, 431=Lenje, 432=Lunda, 433=Ngoni, 435=Bisa, 436=Ila, 437=Nkoya, 438=Lala, 439=Mbunda, 500=Munyoli, 501=Mufumbira, 502=Muhororo, 503=Mukiga, 504=Mukonzo, 505=Munyarwanda, 506=Munyoro, 507=Mutagwenda, 508=Mutooro, 509=Mwamba, 510=Munyankole, 511=Samia, 517=Mutagwenda, 550=Awori, 551=Tapa, 553=Kalabari, 554=Birom, 555=Shuwa-Arab, 556=Jukun, 558=Gwari, 559=Anang, 560=Ekoi, 561=Ukwani, 562=Igede, 563=Ekpeye, 564=Taroh, 565=Ogoni, 566=Sayawa, 567=Okpella, 568=Okirika, 570=Bavudie, 571=Mapokwana, 572=Mantsosa, 573=Baropoli, 575=Balafe, 800=Arab, 801=Swahili, 802=Indian, 803=Digo, 804=Giriama, 805=Duruma, 806=Chonyi, 807=Mijikenda, 808=Arab, 809=Gunya, 810=Teso, 811=Wanga, 812=Kabarasi, 813=Bukusu, 814=Ombuya, 815=Nyala, 816=Vatsotso, 817=Tachoni, 818=Nyore, 819=Maragoli, 820=Marama, 821=Sabaot, 822=Nandi, 823=Kipsigis, 824=Tugen, 825=Keiyo, 826=Ethiopian/Kenyan, 827=Burji, 828=Murule, 829=Dagodia, 830=Gari, 831=Mupule, 832=Shabelle, 833=Gabawen, 834=Garmug, 835=Maimai, 860=Senga, 861=Swahili, 862=Nyanja, 863=Soli, 864=Chokwe, 865=Nyika, 866=Luchazi, 867=Lamba, 868=Kunda, 869=Tabwa, 870=Takaleya, 871=Aushi, 872=Kalunda, 873=Kwamashi, 874=Lambia, 875=Chisinga, 990=National identity only, 995=Other, 997=Not Asked, 998=Refused, 999=Don't know,-1=Missing Data
Source: SAB
Note: Interviewer entered respondent's exact response. If respondent answered "Don't know," said s/he does not belong to any group, or refused to answer, questions 80, 81, and 82 were marked as "Not Applicable" and the interview continued with question 83.

Question Number: Q80A
Question: Think about the condition of ____________ [respondent's identity group] Are their economic conditions worse, the same as, or better than other groups in this country?
Variable Label: Ethnic group's economic conditions
Values: 1-5, 7, 9, 98, -1
Value Labels: 1=Much Better, 2=Better, 3=Same, 4=Worse, 5=Much Worse, 7=Not Applicable, 9=Don't Know, 98=Refused to Answer, -1=Missing Data
Source: SAB
Note: Interviewer probed for strength of opinion. If respondent had not identified a group on question 79, this question was marked as "Not Applicable." Question not asked in Zimbabwe.

Question Number: Q80B
Question: Think about the condition of ____________ [respondent's identity group] Do they have less, the same, or more influence in politics than other groups in this country?
Variable Label: Ethnic group's political influence
Values: 1-5, 7, 9, 98, -1

Value Labels: 1=Much Better, 2=Better, 3=Same, 4=Worse, 5=Much Worse, 7=Not Applicable, 9=Don't Know, 98=Refused to Answer, -1=Missing Data
Source: SAB
Note: Interviewer probed for strength of opinion. If respondent had not identified a group on question 79, this question was marked as "Not Applicable." Question not asked in Zimbabwe.

Question Number: Q81
Question: How often are ___________s [respondent's identity group] treated unfairly by the government?
Variable Label: Ethnic group treated unfairly
Values: 0-3, 7, 9, 98, -1
Value Labels: 0=Never, 1=Sometimes, 2=Often, 3=Always, 7=Not Applicable, 9=Don't Know, 98=Refused to Answer, -1=Missing Data
Source: SAB
Note: Interviewer probed for strength of opinion. If respondent had not identified a group on question 79, this question was marked as "Not Applicable." Question not asked in Zimbabwe.

Question Number: Q82
Question: Let us suppose that you had to choose between being a [Ghanaian/Kenyan/etc.] and being a ________ [respondent's identity group]. Which of these two groups do you feel most strongly attached to?
Variable Label: Ethnic or national identity
Values: 1-5, 7, 9, 98, -1
Value Labels: 1=I Feel Only (r's group), 2=I Feel More (r's group) than [Ghanaian/Kenyan/etc.], 3=I Feel Equally [Ghanaian/Kenyan/etc.] and (r's groups), 4=I Feel More [Ghanaian/Kenyan/etc.] than (r's groups), 5=I feel only [Ghanaian/Kenyan/etc.], 7=Not Applicable, 9=Don't Know, 98=Refused to Answer, -1=Missing Data
Source: SAB
Note: Interviewer probed for strength of opinion. If respondent had not identified a group on question 79, this question was marked as "Not Applicable." Question not asked in Zimbabwe.

Question Number: Q83
Question: Generally speaking, would you say that most people can be trusted or that you must be very careful in dealing with people?
Variable Label: Most people can be trusted
Values: 0-1, 9, 98, -1
Value Labels: 0=You must be very careful, 1=Most people can be trusted, 9=Don't Know, 98=Refused to Answer, -1=Missing Data
Note: Question not asked in Zimbabwe.

Question Number: Q84A
Question: How much do you trust each of the following types of people: Your relatives?
Variable Label: Trust relatives
Values: 0-3, 9, 98, -1
Value Labels: 0=Not at all, 1=Just a little, 2=I trust them somewhat, 3=I trust them a lot, 9=Don't know, 98=Refused to Answer, -1=Missing Data
Note: Question not asked in Zimbabwe.

Question Number: Q84B
Question: How much do you trust each of the following types of people: Your neighbors?
Variable Label: Trust neighbors
Values: 0-3, 9, 98, -1
Value Labels: 0=Not at all, 1=Just a little, 2=I trust them somewhat, 3=I trust them a lot, 9=Don't know, 98=Refused to Answer, -1=Missing Data
Note: Question not asked in Zimbabwe.

Question Number: Q84C
Question: How much do you trust each of the following types of people: People from your own ethnic group?.
Variable Label: Trust people from your ethnic group
Values: 0-3, 9, 97, 98, -1

Copyright Afrobarometer

Value Labels: 0=Not at all, 1=Just a little, 2=I trust them somewhat, 3=I trust them a lot, 9=Don't know, 98=Refused to Answer, -1=Missing Data
Note: Question not asked in Zimbabwe.

Question Number: Q84D
Question: How much do you trust each of the following types of people: [Ghanaian/Kenyan/etc.] from other ethnic groups?
Variable Label: Trust people from other ethnic groups
Values: 0-3, 9, 97, 98, -1
Value Labels: 0=Not at all, 1=Just a little, 2=Somewhat, 3=A lot, 9=Don't know, 98=Refused to Answer, -1=Missing Data
Note: Question not asked in Zimbabwe.

Question Number: Q85
Question: Do you feel close to any particular political party?
Variable Label: Close to political party
Values: 0-1, 8-9, -1
Value Labels: 0=No, not close to any party, 1=Yes, 8=Refused to Answer, 9=Don't Know, -1=Missing Data
Source: Zambia 96

Question Number: Q86
Question: Which party is that?
Variable Label: Which party
Values: 100-107, 109, 111-145, 148, 166-172, 180-184, 201-213, 220-225, 228-231, 240-253, 260-270, 280-285, 287-293, 300-306, 320-328, 340-357, 360-369, 380-397, 420-426, 431, 440-445, 512-517, 850, 851, 995, 997, 998, 999, -1
Value Labels: 100=African Christian Democratic Party (ACDP), 101=African Muslim Party, 102=African National Congress (ANC), 103=Afrikaner Unity Movement, 104=Alliance of Free Democrats, 105=Azanian People's Organization (AZAPO), 106=Christian Party, 107=Freedom Front, 109=Minority Front, 111=Independent Democrats (ID), 112=Inkatha Freedom Party (IFP), 113=Minority Front, 114=National Democratic Convention (NADECO), 115=New National Party (NNP), 116=Pan-Africanist Congress (PAC), 117=Progressive Independent Movement, 118=United Christian Democratic Party (UCDP), 119=United Democratic Movement (UDM), 120=Nicephore SOGLO, 121=Adrien HOUNGBEDJI, 122=Bruno AMOUSSOU, 123=Daniel TAWEMA, 124=Sacca LAFIA, 125=Sefou FAGBOHOUN, 126=KEREKOU, 127=Pascal FANTODJI, 128=CAR, 129=Yayi Boni, 130=RDL, 131=URP, 132=IPD, 133=FADES, 134=EDD, 135=MAP, 136=CAPP SURU, 137=PDB, 138=UPD, 139=PNE, 140=Botswana Alliance Movement (BAM), 141=Botswana Congress Party (BCP), 142=Botswana Democratic Party (BDP), 143=Botswana National Front (BNF), 144=Botswana Independence Party (BIP), 145=MELS, 148=Botswana Peoples Party (BPP), 166=PAICV, 167=MPD, 168=PCD, 169=PRD, 170=UCID, 171=PSD, 172=PTS, 180=CPP, 181=NDC, 182=NPP, 183=PNC, 184=NRP, 201= National Rainbow Coalition (NARC), 202= Liberal Democratic Party (LDP), 203= Democratic Party of Kenya (DP), 204= Ford-Kenya (Ford – K), 205= Ford People (Ford-P), 206= Kenya African National Union (KANU), 207= National Party of Kenya, 208=Shirikisho, 209= Ford Asili (Ford – A), 210= Labour Party of Kenya, 211=SAFINA, 212=Social Democratic Party (SDP), 213=NAK, 220=BNP, 221=BCP, 222=LCP, 223=Popular Front for Democracy (PFD), 224=Marematlou Freedom Party (MFP), 225=Lesotho Peoples Congress (LPC), 228=Lesotho Workers Party (LWP), 229=Basotho African Congress (BFC), 230=Christian Democratic Party (CDP), 231=African National Congress (ANC), 240=Tim, 241=Arema, 242=Affa (CRN), 243=RPSD-Vaovao, 244=Leader-Fanilo, 245=Avi, 246=Akfm Fanavaozana, 247=Teza, 248=Grad Iloafo, 249=UNDD, 250=MFM, 251=Monima, 252=Ame, 253=Independent, 260=Alliance For Democracy (AFORD), 261=Democratic Progressive Party (DPP), 262=MAFUNDE, 263=Malawi Congress Party (MCP), 264=Movement for Genuine Democracy (Mgode),265=National Congress Party (NCP), 266=National Democratic Alliance (NDA), 267=PETRA, 268=Peoples Progressive Movement (PPM), 269=Republic Party (RP), 270=United Democratic Front (UDF), 280=ADEMA, 281=RPM / IBK, 282=CNID, 283=US-RDA, 284=BARA, 285=PARENA, 287=MPR, 288=BDIA, 289=UDO, 290=RND, 291=MIRIA, 292=URD, 293=Mouvement citoyen/ATT, 300=FRELIMO, 301=RENAMO, 302=PIMO, 303=PDD, 304=MBG, 305=PALMO, 306=PASOMO, 320=SWAPO, 321=Democratic Turnhalle Alliance (DTA), 322=Congress of Democrats (CoD), 323=United Democratic Front (UDF), 324=National Unity Democratic Group (Nudo), 325=Namibia Democratic Movement for Change (NDMC), 326=Monitor Action Group (MAG), 327=Republican Party (RP), 328=South West African National Union (SWANU), 340=PDP, 341=AD, 342=ANPP, 343=APGA, 344=PAC, 345=APLG, 346=JP, 347=CPN, 348=NCP, 349=UNPP, 350=NPC, 351=NAP,

352=PRP, 353=MDJ, 354=GPN, 355=PSD, 356=Action Alliance (AA), 357=PSP, 360=PDS, 361=PS, 362=AFP, 363=URD, 364=AJ, 365=PIT, 366=JEF JEL, 367=LD/MPT, 368=Idrissa Seck, 369=Opposition/CPC, 380= Chama cha Mapinduzi (CCM), 381= Civic United Front (CUF), 382= Chama cha Demokrasia na Maendeleo (CHADEMA), 383= Union for Multiparty Democracy (UMD), 384= National Convention for Construction and Reform (NCCR-M), 385= National League for Democracy (NLD), 386= United Peoples' Democratic Party (UPDP), 387= National Reconstruction Alliance (NRA), 388= Tanzania Democratic Alliance Party (TADEA), 389= Tanzania Labour Party (TLP), 390= United Democratic Party (UDP), 391= Demokrasia Makini (MAKINI), 392= Haki na Ustawi (CHAUSTA), 393= The Forum for Restoration of Democracy (FORD), 394= Democratic Party (DP), 395= Progressive Party of Tanzania (PPT), 396=Jahazi Asilia, 397= Sauti ya Umma (SAUTI), 420=Forum for Democracy and Development (FDD), 421=Heritage Party, 422= Movement for Multiparty Democracy (MMD), 423= Patriotic Front (PF), 424= United National Independence Party (UNIP), 425= United Party for National Development (UPND), 426= Zambian Republican Party (ZRP), 431= National Party (NP), 440=ZANU-PF, 441=MDC, 442=ZANU Ndonga, 443=ZUD, 444=ZIYA, 445=NAGG, 512=National Resistence Movement Organisation (NRMO), 513=Forum for Democratic Change (FDC), 514=Uganda Peoples Congress (UPC), 515=Democratic Party (DP), 516=Conservation Party (CP), 517=JEEMA, 850=United Independent Front, 851=United Party of South Africa, 995=Other, 997=Not applicable, 998=Refused, 999=Don't know, -1=Missing Data
Source: Zambia 96
Note: If response to Q85 was "No," "Don't Know," or "Refused to answer," Interviewer marked 7="Not Applicable."

Question Number: Q87
Question: Do you feel very close to this party, somewhat close, or not very close?
Variable Label: How close to this political party
Values: 1-3, 7, 9, 98, -1
Value Labels: 1=Not very close, 2=Somewhat close, 3=Very close, 7=Not Applicable, 9=Don't Know, 98=Refused to Answer, -1=Missing Data
Source: Zambia 96
Note: If response to Q85 was "No," "Don't Know," or "Refused to answer," Interviewer marked 7="Not Applicable."

Question Number: Q90
Question: What is the highest level of education you have completed?
Variable Label: Education of respondent
Values: 0-9, 98-99, -1
Value Labels: 0=No formal schooling, 1=Informal schooling (including Koranic schooling), 2=Some primary schooling, 3=Primary school completed, 4=Some secondary school/ High school, 5=Secondary school completed/High school, 6=Post-secondary qualifications, other than university e.g. a diploma or degree from a technical/polytechnic/college, 7=Some university, 8=University completed, 9=Post-graduate, 98=Refused to Answer, 99=Don't Know, -1=Missing Data

Question Number: Q91
Question: What is your religion, if any?
Variable Label: Religion of respondent
Values: 0, 2-15, 100-108, 200, 241-242, 320-321, 342, 360-363, 995, 998-999, -1
Value Labels: 0=None, 2=Catholic, 3=Protestant (Mainstream), 4=Protestant (Evangelical/Pentecostal), 5=African Independent Church, 6=Traditional religion, 7=Hindu, 8=Agnostic (Do not know if there is a God), 9=Atheist (Do not believe in a God), 10=Christian (General), 11=Muslim, Sunni, 12=Muslim, Shiite, 13=Jehovah's Witness, 14=Seventh Day Adventist, 15=Muslim (general/other), 100=Muslim (general), 101=Assembly of God, 102=Dutch Reform/NG, 103=ZCC, 104=Church of Christ, 105=St. John, 106=AME, 107=VGK,108=Universal Church, 200=Muslim general, 241=Anglican, 242=Protestant flm, 320=Protestant, 321=Other Churches, 342=Other Muslim, 360=Muslim Tijane, 361=Muslim Mouride, 362=Muslim Layene, 363=Muslim Khadre, 995=Other, 998=Refused to Answer, 999=Don't Know, -1=Missing Data
Source: SAB

Question Number: Q92
Question: Excluding weddings and funerals, how often do you attend religious services?

Variable Label: How often attend religious services
Values: 1-6, 9, 98, -1
Value Labels: 1=Never, 2=About once a year or less, 3=About once every several months, 4=About once a month, 5=About once a week, 6=More than once a week, 9=Don’t Know, 98=Refused to Answer, -1=Missing Data
Source: SAB

Question Number: Q93A
Question: Which of these things do you personally own? Book, you know, a reading book.
Variable Label: Own book.
Values: 0-1, 9, 98, -1
Value Labels: 0=No (Don’t own), 1=Yes (Do Own), 9=Don’t Know, 98=Refused to Answer, -1=Missing Data
Source: Afrobarometer Round 3

Question Number: Q93B
Question: Which of these things do you personally own? Radio.
Variable Label: Own radio.
Values: 0-1, 9, 98, -1
Value Labels: 0=No (Don’t own), 1=Yes (Do Own), 9=Don’t Know, 98=Refused to Answer, -1=Missing Data
Source: Afrobarometer Round 3

Question Number: Q93C
Question: Which of these things do you personally own? Television.
Variable Label: Own television.
Values: 0-1, 9, 98, -1
Value Labels: 0=No (Don’t own), 1=Yes (Do Own), 9=Don’t Know, 98=Refused to Answer, -1=Missing Data
Source: Afrobarometer Round 3

Question Number: Q93D
Question: Which of these things do you personally own? Bicycle.
Variable Label: Own bicycle.
Values: 0-1, 9, 98, -1
Value Labels: 0=No (Don’t own), 1=Yes (Do Own), 9=Don’t Know, 98=Refused to Answer, -1=Missing Data
Source: Afrobarometer Round 3

Question Number: Q93E
Question: Which of these things do you personally own? Motorcycle.
Variable Label: Own motorcycle.
Values: 0-1, 9, 98, -1
Value Labels: 0=No (Don’t own), 1=Yes (Do Own), 9=Don’t Know, 98=Refused to Answer, -1=Missing Data
Source: Afrobarometer Round 3

Question Number: Q93F
Question: Which of these things do you personally own? Motor vehicle / car.
Variable Label: Own Motor vehicle or car.
Values: 0-1, 9, 98, -1
Value Labels: 0=No (Don’t own), 1=Yes (Do Own), 9=Don’t Know, 98=Refused to Answer, -1=Missing Data
Source: Afrobarometer Round 3

Question Number: Q94
Question: Do you have a job that pays a cash income? Is it full-time or part-time? And are you presently looking for a job (even if you are presently working)?
Variable Label: Employment status.
Values: 0-5, 9, 98, -1
Value Labels: 0=No (not looking), 1=No (looking), 2=Yes, part time (not looking), 3=Yes, part time (looking), 4=Yes, full time (not looking), 5=Yes, full time (looking), 9=Don’t Know, 98=Refused to Answer, -1=Missing Data
Source: SAB

Question Number: Q95
Question: What is your main occupation? (If unemployed, retired, or disabled, what was your last main occupation?)
Variable Label: Main occupation
Values: 0-25, 201, 220-225, 320-322, 340-342, 990-993, 995, 998-999, -1
Value Labels: 0=Never had a job, 1=Subsistence Farmer (produces only for home consumption), 2=Peasant Farmer (produces both for own consumption and some surplus produce for sale), 3=Commercial Farmer (produces mainly for sale), 4=Farm worker, 5=Fisherman, 6=Trader/Hawker/Vendor, 7=Miner, 8=Domestic Worker/Maid/Char/Househelp, 9=Armed Services/Police/Security Personnel, 10=Artisan/skilled manual worker - formal sector, 11= Artisan/skilled manual worker - informal sector, 12=Clerical Worker, 13= Unskilled manual in the formal sector, 14= Unskilled manual worker in the informal sector, 15= Businessperson (works in the company of others), 16= Businessperson (Owns small business of less than 10 employees), 17=Businessperson (Owns large business of more than 10 employees), 18= Professional Worker (e.g., lawyer, accountant, nurse, engineer, etc.), 19= Supervisor/Foreman, 20=Teacher, 21=Government Worker, 22=Retail worker, 23= Student, 24=Housewife/Works In the Household, 25=Pastoralist/herder/raise livestock, 201=Pastoralist, 220=Priest, 221=Tradtional healer, 222=Sells homemade beer, 223=Herdboy, 224=Disabled, 225=Politician, 320=Employee at NGO, 321=Artisan/skilled manual worker: not sure formal or informal, 322=Unskilled manual worker: not sure formal or informal, 340=Clergy/Imam/Pastor, 341=Musician, 342=Politician, 990=Unemployed, 991=Retired, 992=Disabled, 993=Anything, 995=Other, 998=Refused to Answer, 999=Don't Know, -1=Missing Data

Question Number: Q96A
Question: In the last month, how much of the time: Has your physical health reduced the amount of work you would normally do inside or outside your home?
Variable Label: Physical health
Values: 0-3, 9, 98, -1
Value Labels: 0=Never, 1=Just once or twice, 2=Many times, 3=Always, 9=Don't Know, 98=Refused to Answer, -1=Missing Data
Source: SAB

Question Number: Q96b
Question: In the last month, how much of the time: Have you been so worried or anxious that you have felt tired, worn out, or exhausted?
Variable Label: Mental health
Values: 0-3, 9, 98, -1
Value Labels: 0=Never, 1=Just once or twice, 2=Many times, 3=Always, 9=Don't Know, 98=Refused to Answer, -1=Missing Data
Source: SAB

Question Number: Q97
Question: Do you know a close friend or relative who has died of AIDS?
Variable Label: Know died of AIDS
Values: 0-1, 8-9, -1
Value Labels: 0=No, 1=Yes, 8=Refused to Answer, 9=Don't Know, -1=Missing Data
Source: SAB

Question Number: Q98
Question: How many close friends or relatives do you know who have died of AIDS?
Variable Label: How many died of AIDS
Values: 0-79, 97-99, -1
Value Labels: 997=Not applicable, 998=Refused to Answer, 999=Don't Know, -1=Missing Data
Source: SAB
Note: If response to Q97 was "No", "Don't know" or "Refused to answer", Interviewer marked 997=Not applicable.

Question Number: Q99
Question: If a presidential election were held tomorrow, which party's candidate would you vote for?

Copyright Afrobarometer

Variable Label: Vote for which party
Values: 100-130, 132-137, 140-144, 146-148, 166-172, 180-186, 201-213, 220-226, 228-231, 240-253, 260-270, 280-293, 300-306, 320-330, 340-357, 360-369, 380-397, 420-426, 431, 440-445, 512-517, 850, 851, 990,995, 997, 998, 999, -1
Value Labels: 100=African Christian Democratic Party (ACDP), 101=African Muslim Party, 102=African National Congress (ANC), 103=Afrikaner Unity Movement, 104=Alliance of Free Democrats, 105=Azanian People's Organization (AZAPO), 106=Christian Party, 107=Democratic Alliance-Democratic Party (DA-DP), 108=Federal Alliance, 109=Federal Democrats, 110-Freedom Front, 111=Independent Democrats (ID), 112=Inkatha Freedom Party (IFP), 113=Minority Front, 114=National Democratic Convention (NADECO), 115=New National Party (NNP), 116=Pan-Africanist Congress (PAC), 117=Progressive Independent Movement, 118=United Christian Democratic Party (UCDP), 119=United Democratic Movement (UDM), 120=Nicephore SOGLO, 121=Adrien HOUNGBEDJI, 122=Bruno AMOUSSOU, 123=Daniel TAWEMA, 124=Sacca LAFIA, 125=Sefou FAGBOHOUN, 126=KEREKOU, 127=Pascal FANTODJI, 128=Lionel AGBO, 129=Yayi Boni, 130=RDL, 132=IPD, 133=FADES, 134=EDD, 135=MAP, 136=CAPP SURU, 137=PDB, 140=Botswana Alliance Movement (BAM), 141=Botswana Congress Party (BCP), 142=Botswana Democratic Party (BDP), 143=Botswana National Front (BNF), 144=Botswana Independence Party (BIP), 146=New Democratic Front (UDF), 147=United Socialist Party (USP), 148=Botswana Peoples Party (BPP), 166=PAICV, 167=MPD, 168=PCD, 169=PRD, 170=UCID, 171=PSD, 172=PTS, 180=CPP, 181=NDC, 182=NPP, 183=PNC, 184=NRP, 185=DPP, 186=GCPP, 201= National Rainbow Coalition (NARC), 202= Liberal Democratic Party (LDP), 203= Democratic Party of Kenya (DP), 204= Ford- Kenya (Ford – K), 205= Ford People (Ford-P), 206= Kenya African National Union (KANU), 207= National Party of Kenya (NPK), 208=Shirikisho, 209= Ford Asili (Ford – A), 210= Labour Party of Kenya, 211=SAFINA, 212=Social Democratic Party (SDP), 213=NAK, 220=Basotho National Party (BNP), 221=Basotho Congress Party (BCP), 222=Lesotho Congress for Democracy (LCD), 223=Popular Front for Democracy (PFD), 224=Marematlou Freedom Party (MFP), 225=Lesotho Peoples Congress (LPC), 226=National Progressive Party (NPP), 228=Lesotho Workers Party (LWP), 229=Basotho African Congress (BFC), 230=Christian Democratic Party (CDP), 231=African National Congress (ANC), 240=Tim, 241=Arema, 242=Affa , 243=RPSD-Vaovao, 244=Leader, 245=Avi, 246=Akfm Fanavaozana, 247=Teza, 248=Grad Iloafo, 249=UNDD, 250=MFM, 251=Monima, 252=Ame, 253=Independent, 260=Alliance For Democracy (AFORD), 261=Democratic Progressive Party (DPP), 262=MAFUNDE, 263=Malawi Congress Party (MCP), 264=Movement for Genuine Democracy (Mgode),265=National Congress Party (NCP), 266=National Democratic Alliance (NDA), 267=PETRA, 268=Peoples Progressive Movement (PPM), 269=Republic Party (RP), 270=United Democratic Front (UDF), 280=ADEMA, 281=RPM / IBK, 282=CNID, 283=US-RDA, 284=BARA, 285=PARENA, 286=SADI, 287=MPR, 288=BDIA, 289=UDD, 290=RND, 291=MIRIA, 292=URD, 293=Mouvement citoyen/ATT, 300=FRELIMO, 301=RENAMO, 302=PIMO, 303=PDD, 304=MBG, 305=PALMO, 306=PASOMO, 320=SWAPO, 321=Democratic Turnhalle Alliance (DTA), 322=Congress of Democrats (CoD), 323=United Democratic Front (UDF), 324=National Unity Democratic Group (Nudo), 325=Namibia Democratic Movement for Change (NDMC), 326=Monitor Action Group (MAG), 327=Republican Party (RP), 328=South West African National Union (SWANU), 329=Any opposition party, 330=Ruling Party, 340=PDP, 341=AD, 342=ANPP, 343=APGA, 344=PAC, 345=APLG, 346=JP, 347=CPN, 348=NCP, 349=UNPP, 350=NPC, 351=NAP, 352=PRP, 353=MDJ, 354=GPN, 355=PSD, 356=Action Alliance (AA), 357=PSP, 360=PDS, 361=PS, 362=AFP, 363=URD, 364=AJ, 365=PIT, 366=JEF JEL, 367=LD/MPT, 368=Idrissa Seck, 369=Opposition/CPC, 380= Chama cha Mapinduzi (CCM), 381= Civic United Front (CUF), 382= Chama cha Demokrasia na Maendeleo (CHADEMA), 383= Union for Multiparty Democracy (UMD), 384= National Convention for Construction and Reform (NCCR-M), 385= National League for Democracy (NLD), 386= United Peoples' Democratic Party (UPDP), 387= National Reconstruction Alliance (NRA), 388= Tanzania Democratic Alliance Party (TADEA), 389= Tanzania Labour Party (TLP), 390= United Democratic Party (UDP), 391= Demokrasia Makini (MAKINI), 392= Haki na Ustawi (CHAUSTA), 393= The Forum for Restoration of Democracy (FORD), 394= Democratic Party (DP), 395= Progressive Party of Tanzania (PPT), 396=Jahazi Asilia, 397= Sauti ya Umma (SAUTI), 420=Forum for Democracy and Development (FDD), 421=Heritage Party, 422= Movement for Multiparty Democracy (MMD), 423= Patriotic Front (PF), 424= United National Independence Party (UNIP), 425= United Party for National Development (UPND), 426= Zambian Republican Party (ZRP), 431= National Party (NP), 440=ZANU-PF, 441=MDC, 442=ZANU Ndonga, 443=ZUD, 444=ZIYA, 445=NAGG, 512=National Resistence Movement Organisation (NRMO), 513=Forum for Democratic Change (FDC), 514=Uganda Peoples Congress (UPC), 515=Democratic Party (DP), 516=Conservation Party (CP), 517=JEEMA, 850=United Independent Front, 851=United Party of South Africa, 990=Would vote for candidate and not party, 995=Other, 997=Would not vote, 998=Refused, 999=Don't know, -1=Missing Data
Source: Zambia 96
Note: In Cape Verde, the question read: "Se houvesse amanhã uma eleição legislativa, em que partido votaria?" In Lesotho, the question read: "If general elections were held tomorrow, which party would you vote for?" In South

Africa, it read “Which party would you vote for if a national election were held tomorrow?” In Zambia it read: “Which party would you vote for if a presidential election were held tomorrow?”

Question Number: Q100
Question: Who do you think sent us to do this interview?
Variable Label: Perceived survey sponsor
Values: 0-24, 220-222, 320, 380, 381, 995, 998-999, -1
Value Labels: 0=No one, 1=Government (general), 2=Government (Federal/national), 3=Government (Regional/provincial), 4=Government (Local), 5=President or Prime Minister's Office, 6=Parliament, 7=Government Census/ Statistics Office, 8=National Intelligence/ Secret Service, 9=Education or Social Affairs Department/ Ministry, 10=Tax or Finance Department/ Ministry, 11=Health Department/ Ministry, 12=Other Government department/ Ministry, 13=Constitutional Commission, 14=Electoral Commission, 15=National Planning Commission, 16=Public Utility Company, 17=NGO, 18=Political party/politicians, 19=Research company/ organization/ programme (including the Afrobarometer National Partner), 20=Newspapers/media, 21=University/ school/ college, 22=Private Company, 23=International Organization, 24=God or a Religious organization, 220=Principal Chief, 221=Head Chief, 222=His Majesty King Letsie III, 320=Armed Forces (Police, Army), 380=Government of Zanzibar (SMZ), 381=Human Rights Commission, 995=Other, 998=Refused to Answer, 999=Don't know, -1=Missing Data
Source: Afrobarometer Round 2
Note: Interviewer entered verbatim response

Question Number: Endtime
Question: Time interview ended
Variable Label: Time interview ended
Value Labels: NA
Note: Answered by interviewer. Entered hour and minute, 24 hour clock.

Question Number: Length
Question: Length of interview
Variable Label: Length of interview
Value Labels: NA
Note: Answered by interviewer. Entered hour and minute.

Question Number: Q101
Question: Respondent’s gender
Variable Label: Gender of respondent
Values: 1, 2
Value Labels: 1=Male, 2=Female
Source: SAB
Note: Answered by interviewer.

Question Number: Q102
Question: Respondent’s race
Variable Label: Race of respondent
Values: 1-6, 95
Value Labels: 1=Black/African, 2=White/European, 3=Coloured/Mixed race, 4=Arab/Lebanese/North African, 5=South Asian (Indian, Pakistani, etc.), 6=East Asian(Chinese, Korean, Indonesian, etc.), 95=Other
Source: SAB
Note: Answered by interviewer.

Question Number: Q103
Question: Which languages was the interview conducted in?
Variable Label: Language of interview
Values: 1-4, 100-118, 120-127, 140, 165, 180-185, 200-206, 209-221, 240-241, 260-271, 280-281, 283, 285-289, 292-297, 300-310, 312, 320-328, 331-338, 340-343, 360-369, 406-407, 413-414, 416-423, 440-444, 501, 506, 508, 510, 815, 995, -1
Value Labels: 1=English, 2=French, 3=Portuguese, 4=Kiswahili, 100=Afrikaans, 101=Ndebele, 102=Xhosa, 103=Pedi/Spedi/North Sotho, 104=Sesotho/Sotho/South Sotho, 105=Setswana/Tswana, 106=Shangaan, 107=Swazi,

108=Venda, 109=Zulu, 110=English and Afrikaans, 111=English and Ndebele, 112=English and Xhosa, 113=English and Pedi/N. Sotho, 114=English and Sesotho/S. Sotho, 115=English and Setswana, 116=English and Shangaan, 117=English and Venda, 118=English and Zulu, 120=Fon, 121=Adja, 122=Bariba, 123=Dendi, 124=Yoruba, 125=Otamari, 126=Peulh, 127=Yoa, 140=Setswana, 165=Creole, 180=Akan, 181=Ewe, 182=Ga/, 183=Dagbani, 184=Husa, 185=Other, 200=Kikuyu, 201=Luo, 202=Luhya, 203=Kamba, 204=Meru/Embu, 205=Kisii, 206=Kalenjin, 209=Taita, 210=Somali, 211=Mijikenda, 212=Gunya, 213=Giriama, 214=Kiswahili/Luhya, 215=English/Swahili, 216=Sabaot, 217=Burji, 218=Kuria, 219=Maragoli, 220=Sesotho, 221=Sephuthi, 240=Malg officiel, 241=dialect malg, 260=Tumbuka, 261=Nkhonde, 262=Lambya, 263=Chewa, 264=Yao, 265=Ngoni, 266=Lomwe, 267=Manga'nja, 268=Sena, 269=Sukwa, 270=Senga, 271=Tonga, 280=Bambara, 281=Peuhl/Fulfulde, 283=Mianka, 285=Sonink, 286=Malink, 287=Khasonk, 288=Dogon, 289=Bobo, 292=Maure, 293=Kakolo, 294=Samoko, 295=Sonrha, 296=Bella, 297=Tamasheq, 300=Makua, 301=Sena, 302=Ndau, 303=Nyanja, 304=Changana, 305=Chope, 306=Bitonga, 307=Makonde, 308=Chuabo, 309=Ajua, 310=Quimuane, 312=Nhungue, 320=Nama/Damara, 321=Afrikaans, 322=Rukwangali, 323=Silozi, 324=Masubia, 325=German, 326=Oshiwambo, 327=Otjiherero, 328=Setswana , 331=Mbukushu/Thimbukushu, 332=English and Oshiwambo, 333=English and Afrikaans, 334=Afrikaans and Oshiwambo, 335=English and local languages, 336=Afrikaans and local languages, 337=English, Afrikaans and local languages, 338=More than one local language, 340=Hausa, 341=Igbo, 342=Yoruba, 343=Pidgin English, 360=Wolof, 361=Pular, 362=Serer, 363=Mandinka, 364=Sonike, 365=Diola, 366=Manjack, 367=Bamabara, 368=Bainouk, 369=Bassari, 406=Alur, 407=Ateso, 413=Luo, 414=Lugbara, 416=Madi, 417=Luganda, 418= Lumasaba, 419=Lusoga, 420=Bemba, 421=Nyanja, 422=Tonga, 423=Lozi, 440=Ndebele, 441=Shona, 442=Venda, 443=Kalanga, 444=Tonga, 501=Rufumbira, 506=Runyoro-Rutooro, 508=Rutooro, 510=Runyankole-Rukiga, 815=Boran, 995=other, -1=Missing Data
Source: SAB
Note: Answered by interviewer.

Question Number: Q104
Question: Were there any other people immediately present who might be listening during the interview?
Variable Label: Others present
Values: 1-5, -1
Value Labels: 1=No one, 2=Spouse, 3=Children only, 4=A few others, 5=Small crowd, -1=Missing Data
Source: Nigeria00
Note: Answered by interviewer.

Question Number: Q105A
Question: Did the respondent check with others for information to answer any question?
Variable Label: Check with others
Values: 0, 1, -1
Value Labels: 0=No, 1=Yes, -1=Missing Data
Source: Nigeria00
Note: Answered by interviewer.

Question Number: Q105B
Question: Do you think that anyone influenced the respondent's answers during the interview?
Variable Label: Influence by others
Values: 0, 1, -1
Value Labels: 0=No, 1=Yes, -1=Missing Data
Source: SAB99
Note: Answered by interviewer.

Question Number: Q105C
Question: Were you approached by community and/or political party representatives?
Variable Label: Approached by community/ party representatives
Values: 0, 1
Value Labels: 0=No, 1=Yes
Source: SAB
Note: Answered by interviewer.

Copyright Afrobarometer

Question Number: Q105D
Question: Did you feel threatened during the interview?
Variable Label: Feel threatened
Values: 0, 1
Value Labels: 0=No, 1=Yes
Source: SAB
Note: Answered by interviewer.

Question Number: Q105E
Question: Were you physically threatened during the interview?
Variable Label: Physically threatened
Values: 0, 1
Value Labels: 0=No, 1=Yes
Source: SAB
Note: Answered by interviewer.

Question Number: Q106
Question: What proportion of the questions do you feel the respondent had difficulty answering?
Variable Label: Proportion difficulty answering
Values: 0-4, -1
Value Labels: 0=None, 1=Few, 2=Some, 3=Most, 4=All, -1=Missing Data
Source: Nigeria00
Note: Answered by interviewer.

Question Number: Q107A
Question: Which questions did the respondent have trouble answering?
Variable Label: Trouble answering- first response
Values: 0-100, -1
Value Labels: 0-100, -1=Missing Data
Source: Nigeria00
Note: Answered by interviewer.

Question Number: Q107B
Question: Which questions did the respondent have trouble answering?
Variable Label: Trouble answering- second response
Values: 0-100, -1
Value Labels: 0-100, -1=Missing Data
Source: Nigeria00
Note: Answered by interviewer.

Question Number: Q107C
Question: Which questions did the respondent have trouble answering?
Variable Label: Trouble answering- third response
Values: 0-100, -1
Value Labels: 0-100, -1=Missing Data
Source: Nigeria00
Note: Answered by interviewer.

Question Number: Q108A
Question: What was the respondent's attitude toward you during the interview? Was he or she: friendly, in between, or hostile?
Variable Label: Respondent friendly
Values: 1-3
Value Labels: 1=Friendly, 2=In between, 3=Hostile
Source: SAB
Note: Answered by interviewer.

Question Number: Q108B
Question: What was the respondent's attitude toward you during the interview? Was he or she: interested, in between, or bored?
Variable Label: Respondent interested
Values: 1-3
Value Labels: 1=Interested, 2=In between, 3=Bored
Source: SAB
Note: Answered by interviewer.

Question Number: Q108C
Question: What was the respondent's attitude toward you during the interview? Was he or she: cooperative, in between, or uncooperative?
Variable Label: Respondent cooperative
Values: 1-3
Value Labels: 1=Cooperative, 2=In between, 3=Uncooperative
Source: SAB
Note: Answered by interviewer.

Question Number: Q108D
Question: What was the respondent's attitude toward you during the interview? Was he or she: patient, in between, or impatient?
Variable Label: Respondent patient
Values: 1-3
Value Labels: 1=Patient, 2=In between, 3=Impatient
Source: SAB
Note: Answered by interviewer.

Question Number: Q108E
Question: What was the respondent's attitude toward you during the interview? Was he or she: at ease, in between, or suspicious?
Variable Label: Respondent at ease
Values: 1-3
Value Labels: 1=At ease, 2=In between, 3=Suspicious
Source: SAB
Note: Answered by interviewer.

Question Number: Q108F
Question: What was the respondent's attitude toward you during the interview? Was he or she: honest, in between, or misleading?
Variable Label: Respondent honest
Values: 1-3
Value Labels: 1=Honest, 2=In between, 3=Misleading
Source: SAB
Note: Answered by interviewer.

Question Number: Q110
Question: Interviewer's number
Variable Label: Interviewer's number
Values: BEN01-BEN18, BOT01-BOT24, CVE01-CVE24, GHA01-GHA44, KEN01-KEN83, LES01-LES16, MAD01-MAD28, MLI10-MLI93, MOZ01-MOZ52, MW01-MW16, NAM02-NAM73, NIG00-NIG22, SAF1-SAF99, SEN01-SEN23, TAN01-TAN53, UGA11-UGA84, ZAM01-ZAM53, ZIM01-ZIM20
Source: SAB
Note: Answered by interviewer.

Question Number: Q111
Question: Interviewer's age

Variable Label: Interviewer's age
Values: 24-45
Source: SAB
Note: Answered by interviewer.

Question Number: Q112
Question: Interviewer's gender
Variable Label: Interviewer's gender
Values: 1, 2, -1
Value Labels: 1=Male, 2=Female, -1=Missing Data
Source: SAB
Note: Answered by interviewer.

Question Number: Q113
Question: Do you come from a rural or urban area?
Variable Label: Interviewer urban or rural
Values: 1, 2
Value Labels: 1=Rural, 2=Urban
Source: SAB
Note: Answered by interviewer.

Question Number: Q114
Question: Interviewer's home language
Variable Label: Interviewer's home language
Values: 1-4, 100-109, 120-127, 140, 144-145, 147, 165, 180-185, 200-212, 220, 240-241, 260-271, 280-298, 300-309, 312, 320-328, 340-369, 380-398, 406-407, 413-414, 417-425, 427-428, 440-444, 503, 506,510, 550-569, 580, 995, -1
Value Labels: 1=English, 2=French, 3=Portuguese, 4=Kiswahili, 100=Afrikaans, 101=Ndebele, 102=Xhosa, 103=Pedi/Spedi/North Sotho, 104=Sesotho/Sotho/South Sotho, 105=Setswana/Tswana, 106=Shangaan, 107=Swazi, 108=Venda, 109=Zulu, 120=Fon, 121=Adja, 122=Bariba, 123=Dendi, 124=Yoruba, 125=Otamari, 126=Peulh, 127=Yoa, 140=Setswana, 144=Sekalanga, 145=Seherero, 147=Sebirwa, 165=Creole, 180=Akan, 181=Ewe, 182=Ga, 183=Dagbani, 184=Hausa, 185=Other, 200=Kikuyu, 201=Luo, 202=Luhya, 203=Kamba, 204=Meru/Embu, 205=Kisii, 206=Kalenjin, 207=Masai/Samburu, 208=MijiKenda, 209=Taita, 210=Somali, 211=Maragoli, 212=Luo/English, 220=Sesotho, 240=Malgasy officiel, 241=dialecte, 260=Tumbuka, 261=Nkhonde, 262=Lambya, 263=Chewa, 264=Yao, 265=Ngoni, 266=Lomwe, 267=Manga'nja, 268=Sena, 269=Sukwa, 270=Senga, 271=Tonga, 280=Bambara, 281=Peuhgl/Fulfulde, 282=Senufo, 283=Mianka, 284=Mossi, 285=Soninke, 286=Malinke, 287=Khasonke, 288=Dogon, 289=Bobo, 290=Bozo, 291=Arabe, 292=Maure, 293=Kakolo, 294=Samoko, 295=Sonrhai, 296=Bella, 297=Tamasheq, 298=Dafing, 300=Makua, 301=Sena, 302=Ndau, 303=Nyanja, 304=Changana, 305=Chope, 306=Bitonga, 307=Makonde, 308=Chuabo, 309=Ajua, 312=Quimuane, 320=Nama/Damara, 321=Afrikaans, 322=Rukwangali, 323=Silozi, 324=Masubia, 325=German, 326=Oshiwambo, 327=Otjiherero, 328=Setswana , 340=Hausa, 341=Igbo, 342=Yoruba, 343=Pidgin English, 344=Efik, 345=Ebira, 346=Fulani, 347=Isoko, 348=Ibibio, 349=Kanuri, 350=Tiv, 351=Nupe, 352=Ijaw, 353=Edo, 354=Igala, 355=Urhobo, 356=Idoma, 357=Bassa, 358=Ikwere, 359=Ukwani, 360=Wolof, 361=Pular, 362=Serer, 363=Mandinka, 364=Sonike, 365=Diola, 366=Manjack, 367=Bambara, 368=Bainouk, 369=Bassari, 380=Kinyakyusa, 381=Kichaga, 382=Kihaya, 383=Kingoni, 384=Kikwere, 385=Kipare, 386=Kihehe, 387=Kimakonde, 388=Kinyamwezi, 389=Kisukuma, 390=Kimasai, 391=Kimeru, 392=Kikurya, 393=Kigogo, 394=Kiluguru, 395=Kifipa, 396=Kimanyema, 397=Kinyiramba, 398=Kinyaturu, 406=Alkur, 407=Ateso, 413=Luo, 414=Lugbara, 417=Luganda, 418= Lumasaba, 419=Lusoga, 420=Bemba, 421=Nyanja, 422=Tonga, 423=Lozi, 424=Chewa, 425=Nsenga, 427=Kaonde, 428=Luvale, 440=Ndebele, 441=Shona, 442=Venda, 443=Kalanga, 444=Tonga, 503=Rukiga, 506=Runyoro-Rutooro, 510=Runyankole-Rukiga, 550=Anang, 551=Ron, 552=Geomai, 553=Kadara, 554=Pyam, 555=Bahumono, 556=Boki, 557=Yakurr, 558=Esan, 559=Bijim, 560=Ekpeye, 561=Jaba, 562=Birom, 563=Igede, 564=Pyem, 565=Taroh, 566=Korro, 567=Ogoni, 568=Mbembe, 569=Sayawa, 580=Itsekiri, 995=other, -1=Missing Data
Source: SAB
Note: Answered by interviewer.

Question Number: Q115
Question: Interviewer's highest level of education
Variable Label: Interviewer's education
Values: 3-9, -1
Value Labels: 3=Primary school completed, 4=Some high school, 5=High school completed, 6=Post secondary qualifications (not university), 7=Some university, college, 8=University, college completed, 9=Post graduate, -1=Missing Data
Source: SAB
Note: Answered by interviewer.

Question Number: Q116A
Question: Were the following services present in the primary sampling unit/enumeration area: Post-office?
Variable Label: Post-office in the PSU/EA
Values: 0, 1, 9, -1
Value Labels: 0=No, 1=Yes, 9=Can't determine, -1=Missing Data
Source: SAB
Note: Question was filled in conjunction with field supervisor.

Question Number: Q116B
Question: Were the following services present in the primary sampling unit/enumeration area: School?
Variable Label: School in the PSU/EA
Values: 0, 1, 9, -1
Value Labels: 0=No, 1=Yes, 9=Can't determine, -1=Missing Data
Source: SAB
Note: Question was filled in conjunction with field supervisor.

Question Number: Q116C
Question: Were the following services present in the primary sampling unit/enumeration area: Police station?
Variable Label: Police station in the PSU/EA
Values: 0, 1, 9, -1
Value Labels: 0=No, 1=Yes, 9=Can't determine, -1=Missing Data
Source: SAB
Note: Question was filled in conjunction with field supervisor.

Question Number: Q116D
Question: Were the following services present in the primary sampling unit/enumeration area: Electricity grid that most houses could access?
Variable Label: Electricity grid in the PSU/EA
Values: 0, 1, 9, -1
Value Labels: 0=No, 1=Yes, 9=Can't determine, -1=Missing Data
Source: SAB
Note: Question was filled in conjunction with field supervisor.

Question Number: Q116E
Question: Were the following services present in the primary sampling unit/enumeration area: Piped water system that most houses could access?
Variable Label: Piped water system in the PSU/EA
Values: 0, 1, 9, -1
Value Labels: 0=No, 1=Yes, 9=Can't determine, -1=Missing Data
Source: SAB
Note: Question was filled in conjunction with field supervisor.

Question Number: Q116F
Question: Were the following services present in the primary sampling unit/enumeration area: Sewage system that most houses could access?
Variable Label: Sewage system in the PSU/EA
Values: 0, 1, 9, -1
Value Labels: 0=No, 1=Yes, 9=Can't determine, -1=Missing Data

Source: SAB
Note: Question was filled in conjunction with field supervisor.

Question Number: Q116G
Question: Were the following services present in the primary sampling unit/enumeration area: Health clinic?
Variable Label: Health clinic in the PSU/EA
Values: 0, 1, 9, -1
Value Labels: 0=No, 1=Yes, 9=Can't determine, -1=Missing Data
Source: SAB
Note: Question was filled in conjunction with field supervisor.

Question Number: Q116H
Question: Were the following services present in the primary sampling unit/enumeration area: Recreational facilities, e.g., a sports field?
Variable Label: Recreational facilities in the PSU/EA
Values: 0, 1, 9, -1
Value Labels: 0=No, 1=Yes, 9=Can't determine, -1=Missing Data
Source: SAB
Note: Question was filled in conjunction with field supervisor.

Question Number: Q116I
Question: Were the following services present in the primary sampling unit/enumeration area: Any churches, mosques, temples or other places of worship?
Variable Label: Places of worship in the PSU/EA
Values: 0, 1, 9, -1
Value Labels: 0=No, 1=Yes, 9=Can't determine, -1=Missing Data
Source: SAB
Note: Question was filled in conjunction with field supervisor.

Question Number: Q116J
Question: Were the following services present in the primary sampling unit/enumeration area: Any town halls or community buildings that can be used for meetings?
Variable Label: Community buildings in the PSU/EA
Values: 0, 1, 9, -1
Value Labels: 0=No, 1=Yes, 9=Can't determine, -1=Missing Data
Source: SAB
Note: Question was filled in conjunction with field supervisor.

Question Number: Q116K
Question: Were the following services present in the primary sampling unit/enumeration area: Market stalls (selling groceries and/or clothing)?
Variable Label: Market stalls in the PSU/EA
Values: 0, 1, 9, -1
Value Labels: 0=No, 1=Yes, 9=Can't determine, -1=Missing Data
Source: SAB
Note: Question was filled in conjunction with field supervisor.

Question Number: Q117A
Question: In the PSU/EA, did you (or any of your colleagues) see: Any policemen or police vehicles?
Variable Label: Police in the PSU/EA
Values: 0, 1, 9, -1
Value Labels: 0=No, 1=Yes, 9=Can't determine, -1=Missing Data
Source: SAB
Note: Question was filled in conjunction with field supervisor.

Question Number: Q117B
Question: In the PSU/EA, did you (or any of your colleagues) see: Any soldiers or army vehicles?

Variable Label: Soldiers/army in the PSU/EA
Values: 0, 1, 9, -1
Value Labels: 0=No, 1=Yes, 9=Can't determine, -1=Missing Data
Source: SAB
Note: Question was filled in conjunction with field supervisor.

Question Number: Q118
Question: Thinking of your journey here: Was the road at the start point in the PSU/EA paved/ tarred/ concrete?
Variable Label: Tarred/Paved road
Values: 0, 1, -1
Value Labels: 0=No, 1=Yes, -1=Missing Data
Source: SAB
Note: Question was filled in conjunction with field supervisor.

Question Number: withinwt
Variable Label: Within country weight
Note: This variable adjusts the distribution of each country sample to take account of over- or under-samples with respect to region, urban-rural distribution, or other factors as indicated below.

Cape Verde: In Cape Verde, the weighting variable adjusts the distribution of the sample to take account of small over- or under-samples across the four islands included in the sample, and in the urban-rural distribution.

		Unweighted Proportion	Weighted Proportion
**Island**			
Santo Antão	Urban	3.7	3.3
	Rural	13.9	9.7
São Vicente	Urban	16.3	14.2
	Rural	1.3	1.2
Santiago-Interior	Urban	5.7	6.4
	Rural	21.1	30.0
Santiago-Praia	Urban	17.8	21.5
	Rural	2.5	3.2
Fogo	Urban	3.8	1.9
	Rural	13.8	8.6

Kenya*:* In Kenya, the weighting variable adjusts the distribution of the sample to take account of over- or under-samples with respect to province and urban-rural distribution.

		Unweighted Proportion	Weighted Proportion
**Province**			
Central	Urban	1.9	1.3
	Rural	10.0	11.8
Coast	Urban	5.0	3.2
	Rural	5.0	5.5
Eastern	Urban	1.9	1.0
	Rural	13.1	15.2
Nairobi	Urban	11.3	7.4
	Rural	0.0	0.0
North Eastern	Urban	0.6	0.5
	Rural	4.4	2.4
Nyanza	Urban	2.5	1.5
	Rural	11.9	13.9
Rift Valley	Urban	4.4	3.4

Copyright Afrobarometer

	Rural	16.7	20.9
Western	Urban	1.9	1.0
	Rural	9.4	10.9

Lesotho: In Lesotho, the weighting variable adjusts the distribution of the sample to take account of a rural under-sample in two districts.

	Unweighted Proportion	Weighted Proportion
Berea		
Urban	2.8	2.7
Rural	9.0	8.7
Butha Buthe		
Urban	3.4	3.3
Rural	4.0	4.0
Leribe		
Urban	4.8	4.7
Rural	7.8	10.7
Mafeteng		
Urban	3.4	3.3
Rural	9.0	8.7
Maseru		
Urban	5.5	5.3
Rural	11.0	10.7
Mohale’s Hoek		
Urban	3.4	3.3
Rural	7.6	7.3
Mokhotlong		
Urban	2.8	2.7
Rural	4.1	4.0
Thaba-Tseka		
Urban	2.8	2.7
Rural	5.5	5.3
Qacha’s Nek		
Urban	2.8	2.7
Rural	2.8	2.7
Quthing		
Urban	2.8	2.7
Rural	4.8	4.7

Mali: In Mali, the weighting variable adjusts the distribution of the sample to take account of over- or under-samples with respect to region.

	Unweighted Proportion	Weighted Proportion
**Region**		
Bamako		
Urban	10.3%	10.4%
Rural	0.0%	0.0%
Gao		
Urban	1.4%	1.4%
Rural	2.7%	2.6%
Kayes		
Urban	2.6%	2.6%
Rural	10.9%	11.4%
Kidal		
Urban	0.6%	0.2%

Rural	1.9%	0.3%
Koulikoro		
Urban	2.6%	2.6%
Rural	12.9%	13.4%
Mopti		
Urban	1.9%	2.0%
Rural	12.9%	13.1%
Ségou		
Urban	3.2%	3.0%
Rural	14.1%	14.0%
Sikasso		
Urban	3.9%	3.9%
Rural	13.5%	14.3%
Tombouctou		
Urban	0.7%	1.0%
Rural	3.9%	3.9%

Nigeria: In Nigeria, the weighting variable adjusts the distribution of the sample to take account of an over-sample in Bayelsa, Delta and Rivers states in South South Region, and an undersample in Northwest Region.

		Unweighted Proportion	Weighted Proportion
**Region**			
Lagos	Urban	8.7	9.3
	Rural	0.6	0.7
North Central	Urban	5.2	5.6
	Rural	7.9	8.4
North East	Urban	4.4	4.7
	Rural	8.1	8.7
North West	Urban	8.0	8.9
	Rural	11.8	13.3
South East	Urban	5.6	6.0
	Rural	5.6	6.0
South South	Urban	9.4	6.1
	Rural	12.4	9.1
South West	Urban	7.4	7.9
	Rural	4.8	5.2

South Africa: In South Africa, the weighting variable adjusts the distribution of the sample based on individual selection probabilities (i.e., based on province, gender, urban-rural distribution, and size of household and enumeration area).

		Unweighted Proportion	Weighted Proportion
**Gender**			
	Male	50.0	50.1
	Female	50.0	49.9
**Location**			
	Urban	53.3	49.6
	Rural	15.0	14.8
	Rural Farm	4.3	4.3
	Informal	8.2	7.6
	Institution	0.3	0.5
	Tribal	18.7	23.1

	Other	0.2	0.2
**Province**			
	Eastern Cape	16.8	13.4
	Free State	7.8	6.6
	Gauteng	16.8	15.2
	Kwazulu Natal	16.2	16.9
	Limpopo	11.2	17.2
	Mpumalanga	7.8	8.5
	North West	8.5	10.4
	Northern Cape	3.3	4.8
	Western Cape	11.5	6.9
**Race**			
	Black/African	75.2	78.2
	White/European	11.8	11.0
	Coloured/Mixed Race	10.0	7.9
	Arab/Lebanese/North Afr.	0.0	0.0
	South Asian	2.8	2.8
	East Asian	0.0	0.0
	Other	0.1	0.1

Tanzania: In Tanzania, The weighting variable adjusts the distribution of the sample to take account of an over-sample in Zanzibar relative to the Mainland, and to correct the urban-rural distribution.

		**Unweighted Proportion**	**Weighted Proportion**
**Region**			
Mainland	Urban	20.9	22.0
	Rural	73.0	75.2
Zanzibar	Urban	1.8	1.1
	Rural	4.3	1.8

Uganda: In Uganda, the weighting variable adjusts the distribution of the sample to take account of oversamples or undersamples with respect to urban or rural location.

	**Unweighted Proportion**	**Weighted Proportion**
Urban	30.0	12.4
Rural	70.0	87.6

Note: There were no within-country weights in Benin, Botswana, Ghana, Madagascar, Malawi, Mozambique, Namibia, Senegal, Zambia and Zimbabwe.

Question Number: acrosswt
Variable Label: Across country weight (N=1200 for all)
Note: This weight adjusts all country samples to the same size, N=1200.

**Country**	**N Unweighted**	**Weight**	**N Weighted**
Benin	1198	1.002	1200
Botswana	1200	1.000	1200
Cape Verde	1256	0.955	1200

Ghana	1197	1.003	1200
Kenya	1278	0.939	1200
Lesotho	1161	1.034	1200
Madagascar	1350	0.889	1200
Malawi	1200	1.000	1200
Mali	1244	0.965	1200
Mozambique	1198	1.002	1200
Namibia	1200	1.000	1200
Nigeria	2363	0.508	1200
Senegal	1200	1.000	1200
South Africa	2400	0.5	1200
Tanzania	1304	0.920	1200
Uganda	2400	0.5	1200
Zambia	1200	1.000	1200
Zimbabwe	1048	1.145	1200

Question Number: combinwt
Variable Label: Combined weight (withinwt*acrosswt)
Note: This weight combines the within-country weights with the across-country weights to produce a single weighting factor that can be applied in analyzing the results.
