# Supplementary material for: Mancala board games and origins of entrepreneurship in Africa
Source: PLoS One. 2020 Oct 15;15(10):e0240790. doi: 10.1371/journal.pone.0240790 (PMC7561206; doi:10.1371/journal.pone.0240790)
Supplement: S1 File — This zip file contains the underlying datasets, R code and the STATA do-file used to replicate the results of the manuscript. (ZIP) [file pone.0240790.s004.zip › replicationfiles/Readme.docx]

**Readme file for “Mancala board games and entrepreneurship in Africa”**

The replication files folder contains all data, do files, R scripts, graphs and tables presented in the paper.

First run the R scripts in the folder “R”. Run the ggAVPLOTS.R, a function for added variable plots. Then run the OriginsRCode.R script. Make sure to change the path to the directory containing the replication files.

The R scripts have all the results as in the paper. However, the tables in the paper are based on the Stata do file in the “do” folder. Run the do file “Originspaper.do” after changing the path on line 11 to your directory containing the replication files.

The “dta” folder contains all datasets used in the paper including the mancala database which has additional data. The “shp” folder contains all the shapefiles for the spatial data presented in the paper including the spatial location of the mancala games and the ethnic groups. It also contains the QGIS program for creating figure 3.
