# Supplementary material for: Mancala board games and origins of entrepreneurship in Africa
Source: PLoS One. 2020 Oct 15;15(10):e0240790. doi: 10.1371/journal.pone.0240790 (PMC7561206; doi:10.1371/journal.pone.0240790)
Supplement: S1 File — This zip file contains the underlying datasets, R code and the STATA do-file used to replicate the results of the manuscript. (ZIP) [file pone.0240790.s004.zip › replicationfiles/tables/gamesinculturefinal.rtf]

	(1)	(2)	(3)	(4)	
	SocialComplexityDummy	SocialComplexityDummy	EconomicComplexityDummy	EconomicComplexityDummy	
Game complexity	-0.0522	0.00806	-0.180	-0.118	
	(0.110)	(0.117)	(0.112)	(0.114)	
N	81	76	81	76	
R2	0.003	0.050	0.032	0.107	
adj. R2	-0.010	0.010	0.020	0.070	
Standard errors in parentheses
* p < 0.05, ** p < 0.01, *** p < 0.001
