# Supplementary material for: Mancala board games and origins of entrepreneurship in Africa
Source: PLoS One. 2020 Oct 15;15(10):e0240790. doi: 10.1371/journal.pone.0240790 (PMC7561206; doi:10.1371/journal.pone.0240790)
Supplement: S1 File — This zip file contains the underlying datasets, R code and the STATA do-file used to replicate the results of the manuscript. (ZIP) [file pone.0240790.s004.zip › replicationfiles/tables/originsmodelfinal.rtf]

	(1)	(2)	(3)	(4)	(5)	
	occupationdummy	occupationdummy	occupationdummy	occupationdummy	occupationdummy	
Game complexity	0.0548	0.0191	-0.0589*			
	(0.0596)	(0.0441)	(0.0263)			
						
Islam		-0.0709	-0.0452*	-0.0459*	-0.0332	
		(0.0361)	(0.0196)	(0.0198)	(0.0454)	
						
frac_ethnicity_in_district		0.0859**	0.137***	0.186***	0.187***	
		(0.0317)	(0.0269)	(0.0362)	(0.0360)	
						
SlaveTrade		-0.0141	0.00329	-0.000538	0.000320	
		(0.0292)	(0.0335)	(0.0339)	(0.0337)	
						
AgDependenceDummy		-0.0244	-0.00404	-0.00348	-0.00403	
		(0.0367)	(0.0355)	(0.0358)	(0.0359)	
						
Game complexity				0.00695	0.00976	
				(0.0405)	(0.0398)	
						
1.GameTypeComplexityDummy#c.frac_ethnicity_in_district				-0.0987	-0.0997	
				(0.0513)	(0.0514)	
						
1.GameTypeComplexityDummy#c.Islam					-0.0173	
					(0.0466)	
N	3576	2917	2917	2917	2917	
R2	0.003	0.307	0.349	0.350	0.350	
adj. R2	0.003	0.303	0.341	0.342	0.342	
Standard errors in parentheses
* p < 0.05, ** p < 0.01, *** p < 0.001
