# Supplementary material for: Mancala board games and origins of entrepreneurship in Africa
Source: PLoS One. 2020 Oct 15;15(10):e0240790. doi: 10.1371/journal.pone.0240790 (PMC7561206; doi:10.1371/journal.pone.0240790)
Supplement: S1 File — This zip file contains the underlying datasets, R code and the STATA do-file used to replicate the results of the manuscript. (ZIP) [file pone.0240790.s004.zip › replicationfiles/tables/originsmodelfinalMigrants.rtf]

	(1)	(2)	(3)	(4)	(5)	
	occupationdummy	occupationdummy	occupationdummy	occupationdummy	occupationdummy	
Game complexity	0.0287	-0.0450	-0.0566			
	(0.0718)	(0.0532)	(0.0371)			
						
Islam		-0.0804	-0.0505	-0.0441	-0.0782	
		(0.0409)	(0.0269)	(0.0259)	(0.0752)	
						
frac_ethnicity_in_district		0.0748	0.108**	0.210***	0.208***	
		(0.0419)	(0.0362)	(0.0535)	(0.0523)	
						
SlaveTrade		-0.0320	0.0566	0.0454	0.0433	
		(0.0453)	(0.0389)	(0.0361)	(0.0362)	
						
AgDependenceDummy		-0.00510	-0.00838	0.00156	0.00294	
		(0.0518)	(0.0499)	(0.0477)	(0.0475)	
						
Game complexity				0.0426	0.0359	
				(0.0467)	(0.0445)	
						
1.GameTypeComplexityDummy#c.frac_ethnicity_in_district				-0.187**	-0.184**	
				(0.0615)	(0.0604)	
						
1.GameTypeComplexityDummy#c.Islam					0.0457	
					(0.0801)	
N	2011	1585	1585	1585	1585	
R2	0.001	0.349	0.387	0.391	0.391	
adj. R2	0.000	0.340	0.374	0.378	0.378	
Standard errors in parentheses
* p < 0.05, ** p < 0.01, *** p < 0.001
