# Supplementary figures and images for: Mancala board games and origins of entrepreneurship in Africa
Source: PLoS One. 2020 Oct 15;15(10):e0240790. doi: 10.1371/journal.pone.0240790 (PMC7561206; doi:10.1371/journal.pone.0240790)

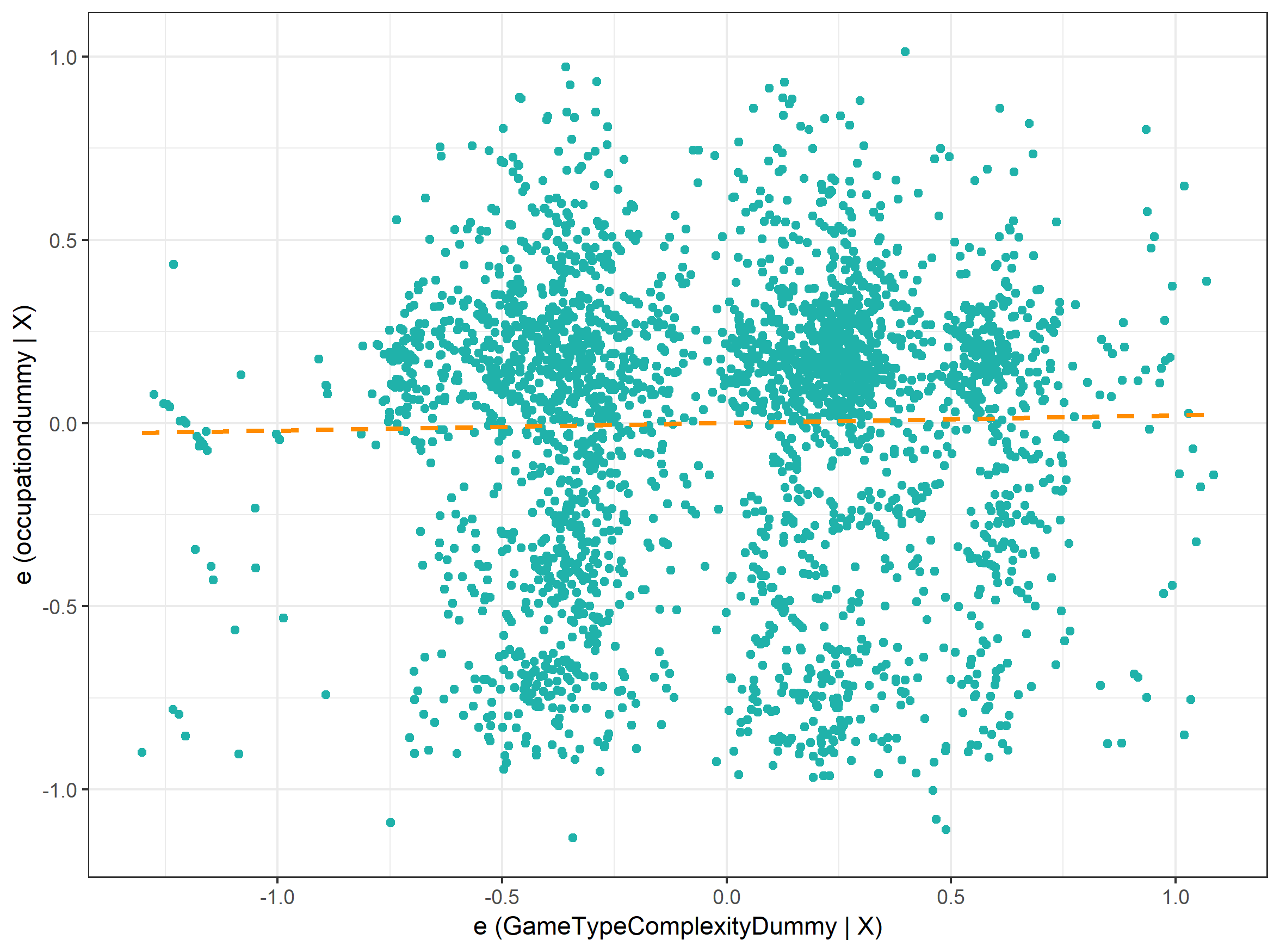

Supplement: S1 File — This zip file contains the underlying datasets, R code and the STATA do-file used to replicate the results of the manuscript. (ZIP) [file pone.0240790.s004.zip › replicationfiles/Graphs/AvPlotOccTypeComplexity.png]

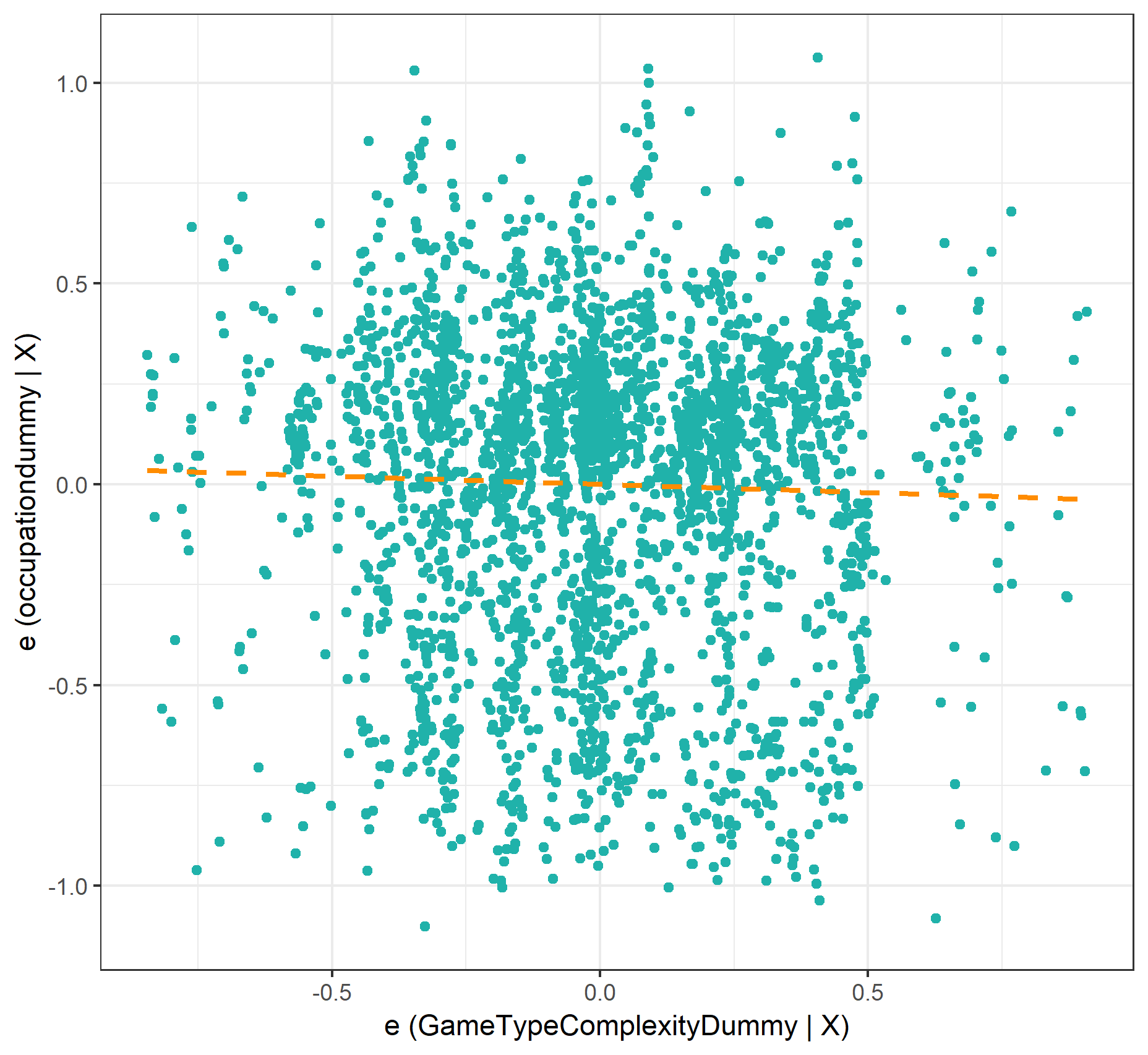

Supplement: S1 File — This zip file contains the underlying datasets, R code and the STATA do-file used to replicate the results of the manuscript. (ZIP) [file pone.0240790.s004.zip › replicationfiles/Graphs/AvPlotOccTypeComplexityCountryFE.png]

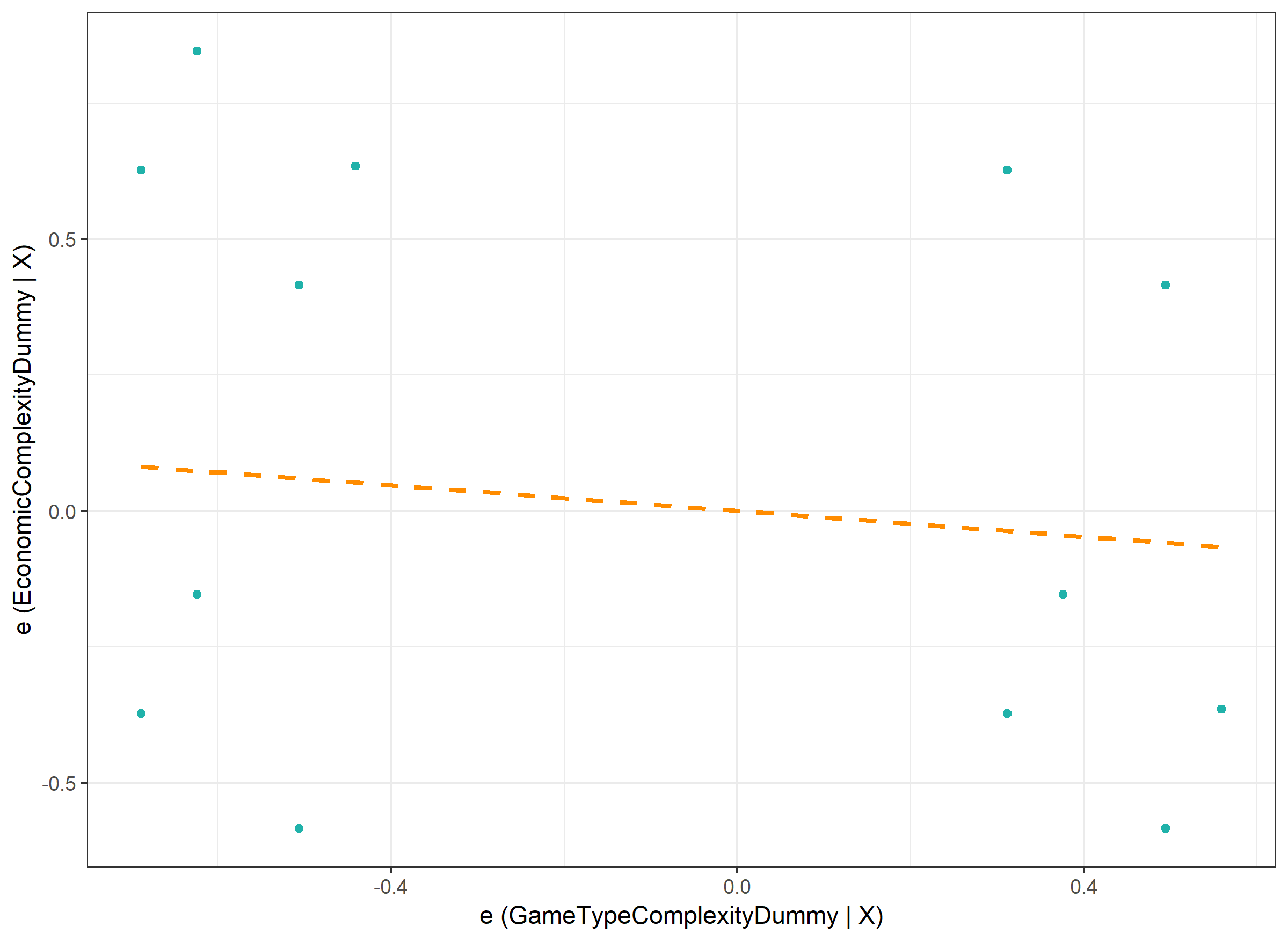

Supplement: S1 File — This zip file contains the underlying datasets, R code and the STATA do-file used to replicate the results of the manuscript. (ZIP) [file pone.0240790.s004.zip › replicationfiles/Graphs/AvPlotsHistoricalEcon.png]

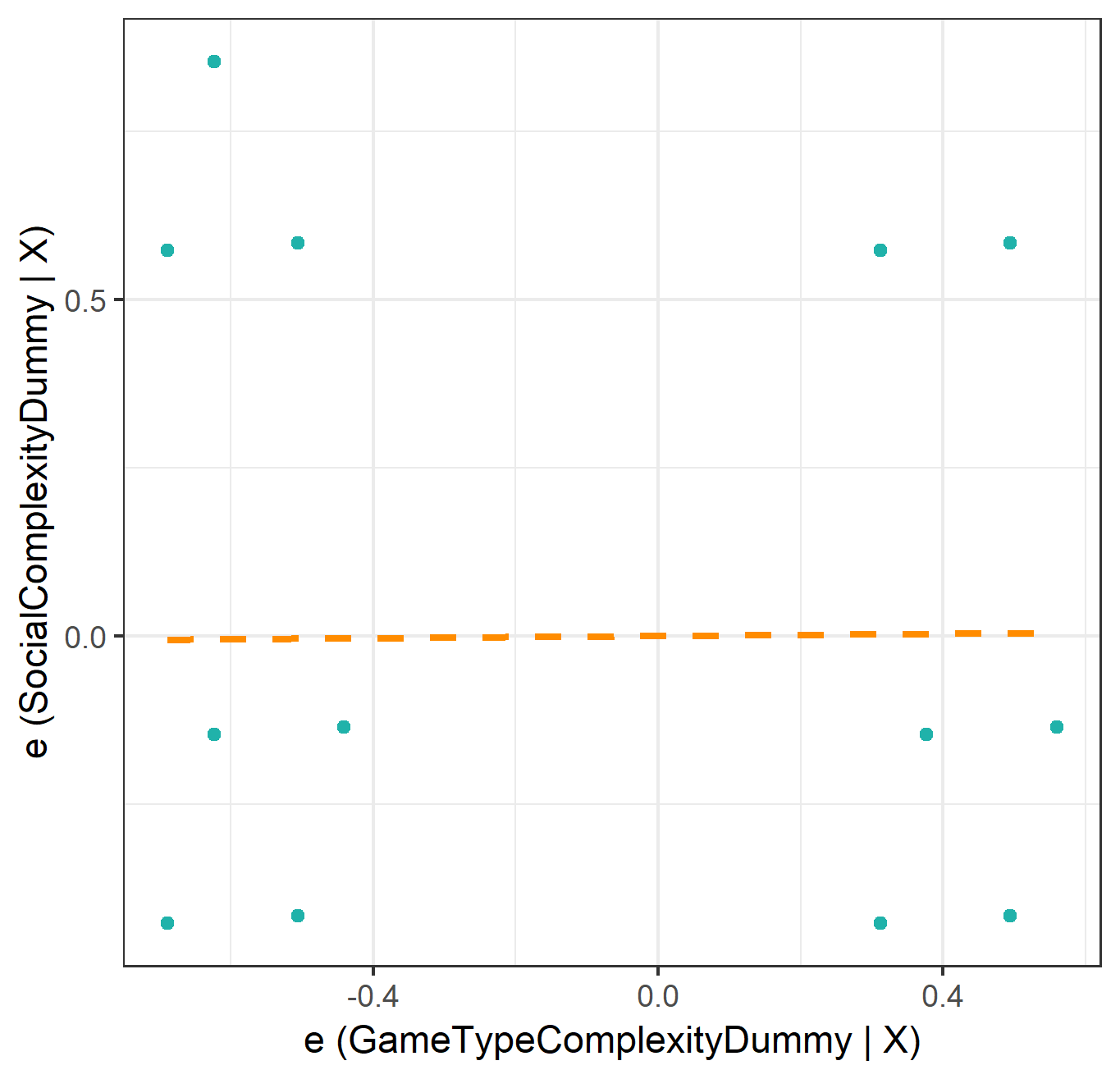

Supplement: S1 File — This zip file contains the underlying datasets, R code and the STATA do-file used to replicate the results of the manuscript. (ZIP) [file pone.0240790.s004.zip › replicationfiles/Graphs/AvPlotsHistoricalSoc.png]

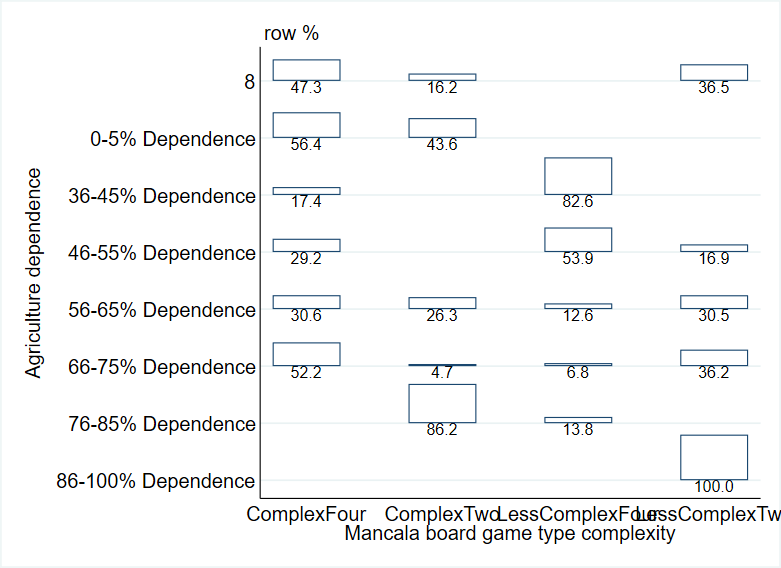

Supplement: S1 File — This zip file contains the underlying datasets, R code and the STATA do-file used to replicate the results of the manuscript. (ZIP) [file pone.0240790.s004.zip › replicationfiles/Graphs/ComtemporaryGameComplexityandAg.png]

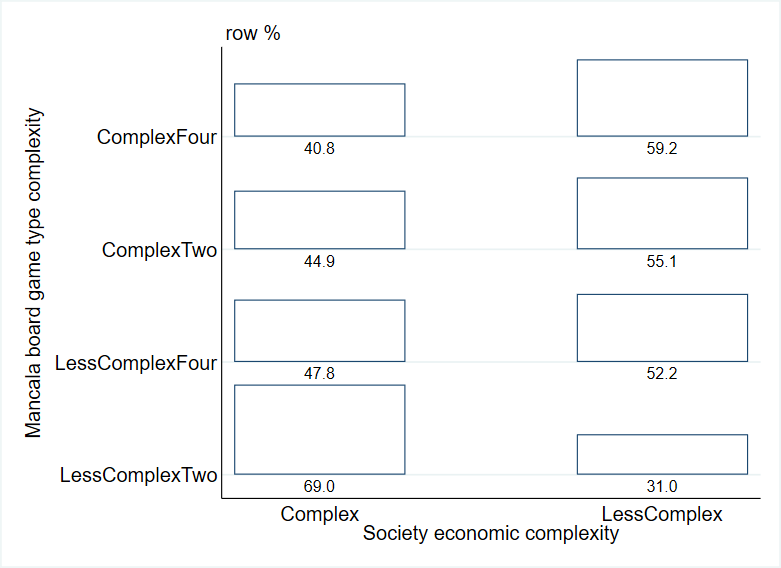

Supplement: S1 File — This zip file contains the underlying datasets, R code and the STATA do-file used to replicate the results of the manuscript. (ZIP) [file pone.0240790.s004.zip › replicationfiles/Graphs/ComtemporaryGameComplexityandEconCompl.png]

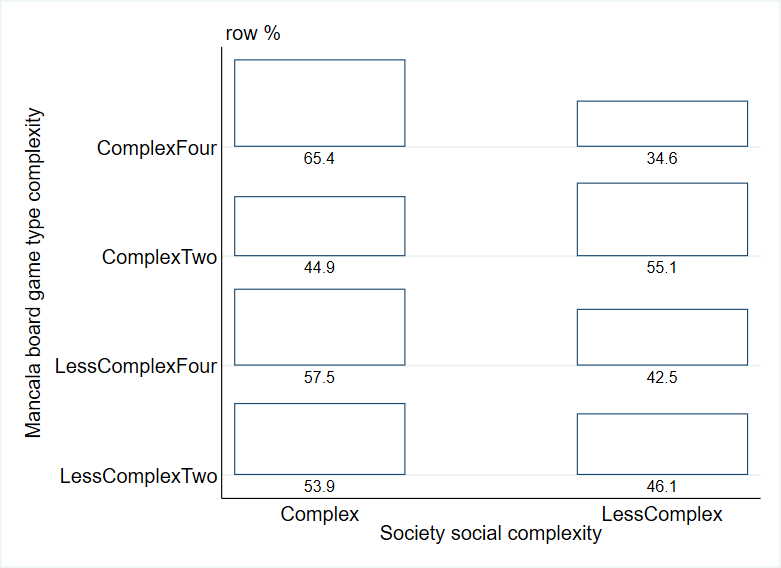

Supplement: S1 File — This zip file contains the underlying datasets, R code and the STATA do-file used to replicate the results of the manuscript. (ZIP) [file pone.0240790.s004.zip › replicationfiles/Graphs/ComtemporaryGameComplexityandSocialCompl.png]

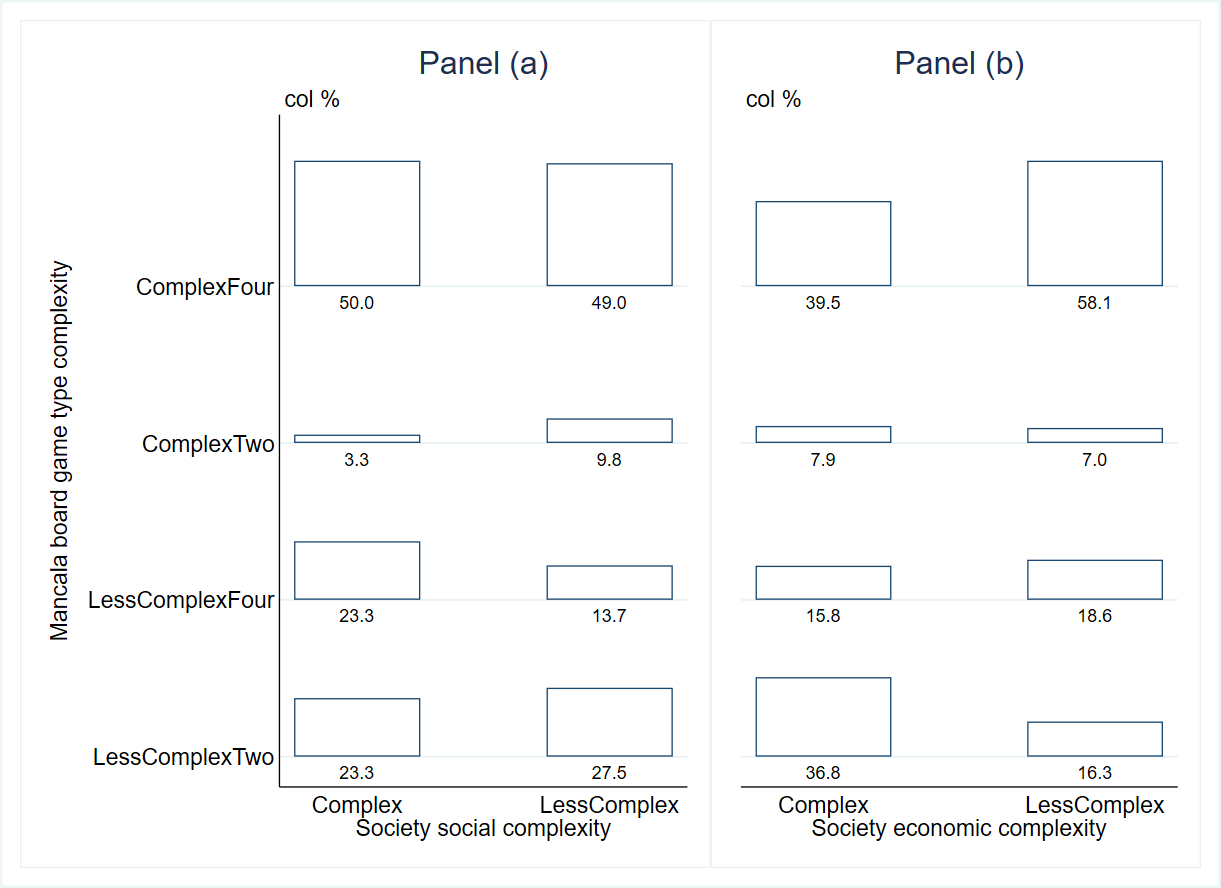

Supplement: S1 File — This zip file contains the underlying datasets, R code and the STATA do-file used to replicate the results of the manuscript. (ZIP) [file pone.0240790.s004.zip › replicationfiles/Graphs/Fig4Final.png]

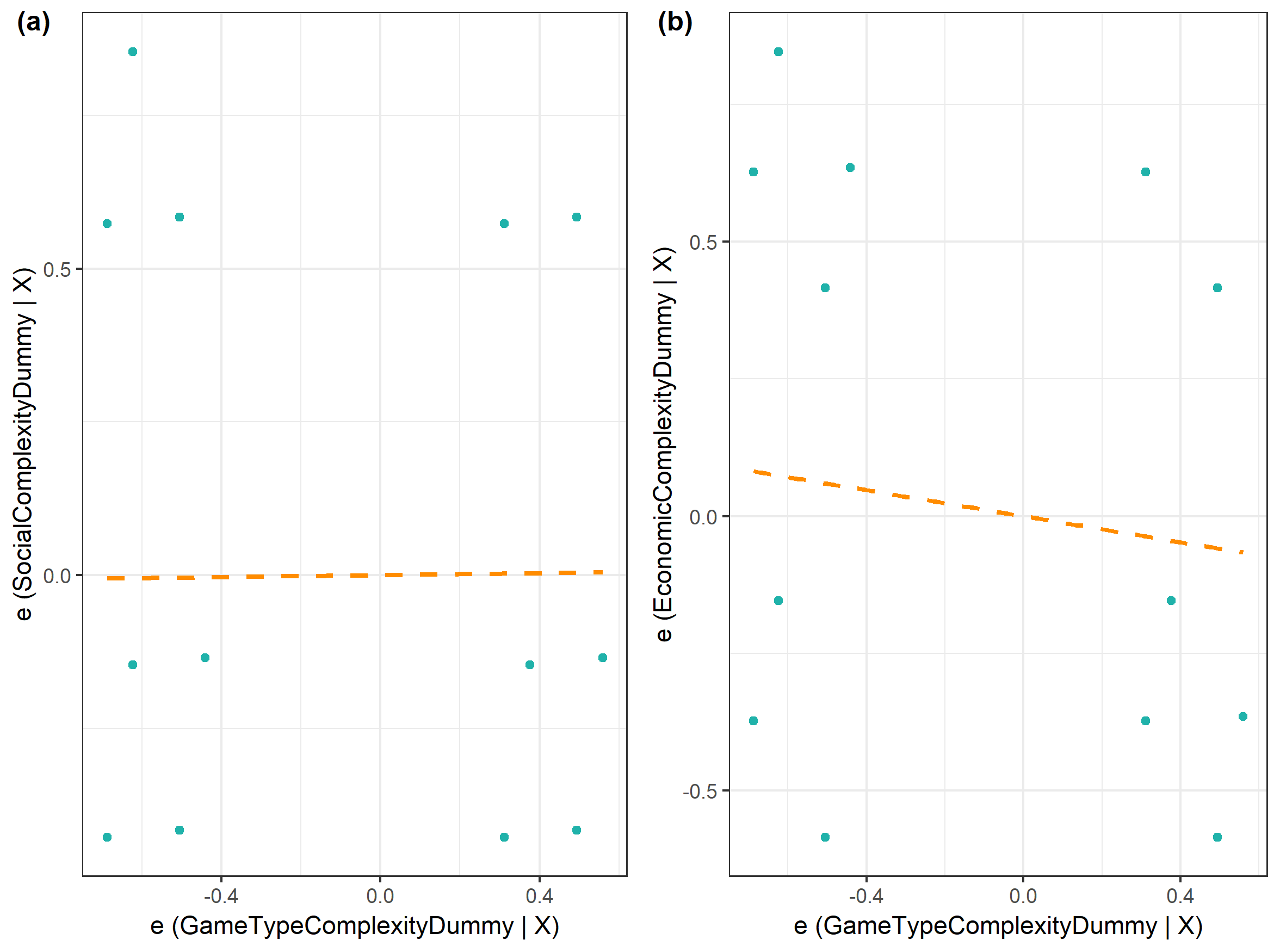

Supplement: S1 File — This zip file contains the underlying datasets, R code and the STATA do-file used to replicate the results of the manuscript. (ZIP) [file pone.0240790.s004.zip › replicationfiles/Graphs/Fig5Final.png]

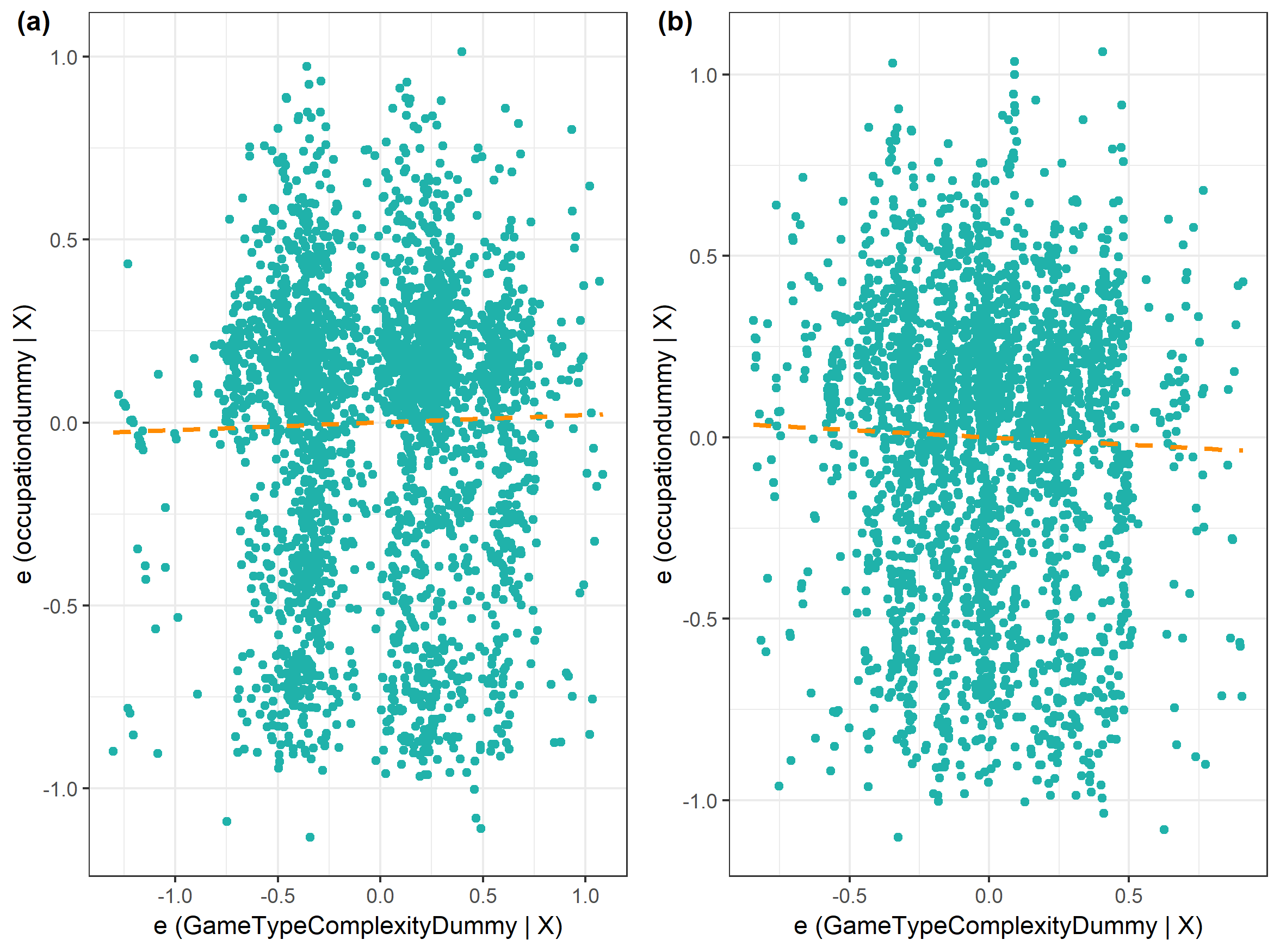

Supplement: S1 File — This zip file contains the underlying datasets, R code and the STATA do-file used to replicate the results of the manuscript. (ZIP) [file pone.0240790.s004.zip › replicationfiles/Graphs/Fig6Final.png]

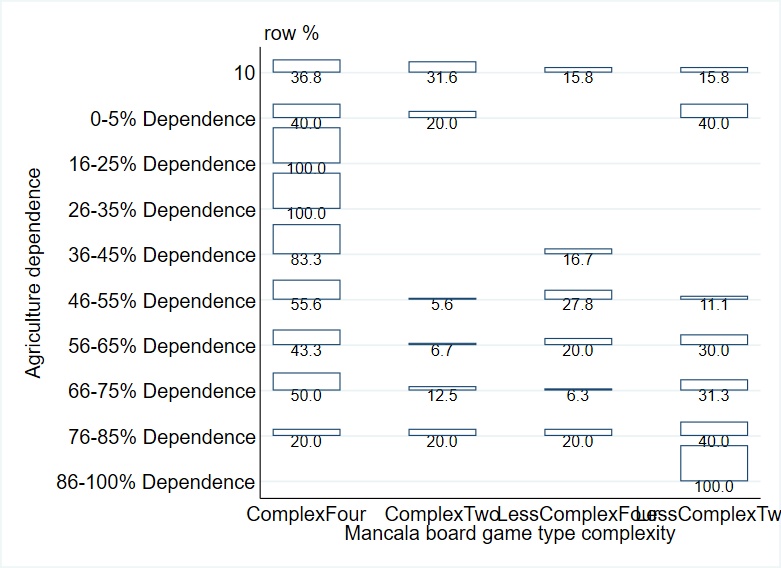

Supplement: S1 File — This zip file contains the underlying datasets, R code and the STATA do-file used to replicate the results of the manuscript. (ZIP) [file pone.0240790.s004.zip › replicationfiles/Graphs/GameComplexityandAg.png]

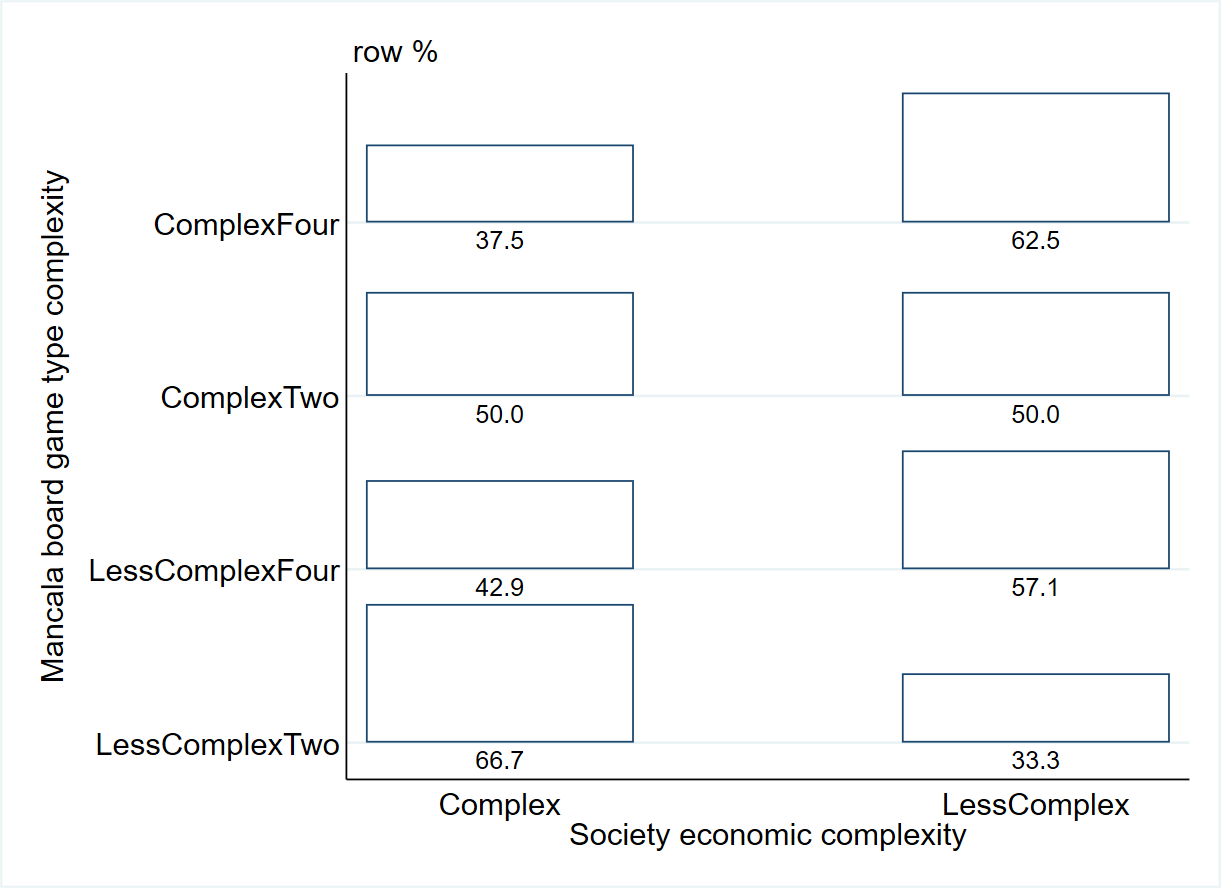

Supplement: S1 File — This zip file contains the underlying datasets, R code and the STATA do-file used to replicate the results of the manuscript. (ZIP) [file pone.0240790.s004.zip › replicationfiles/Graphs/GameComplexityandEconCompl.png]

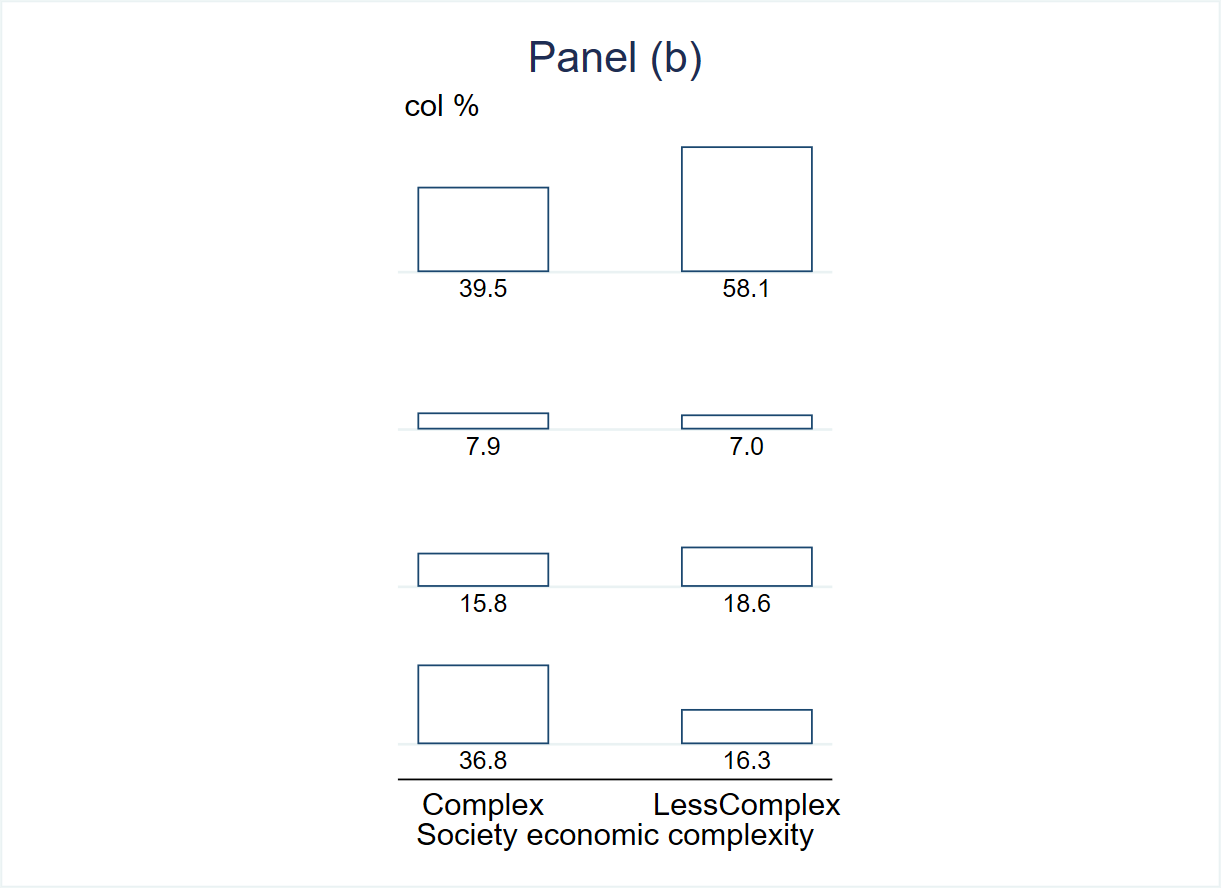

Supplement: S1 File — This zip file contains the underlying datasets, R code and the STATA do-file used to replicate the results of the manuscript. (ZIP) [file pone.0240790.s004.zip › replicationfiles/Graphs/GameComplexityandEconCompl_Col.png]

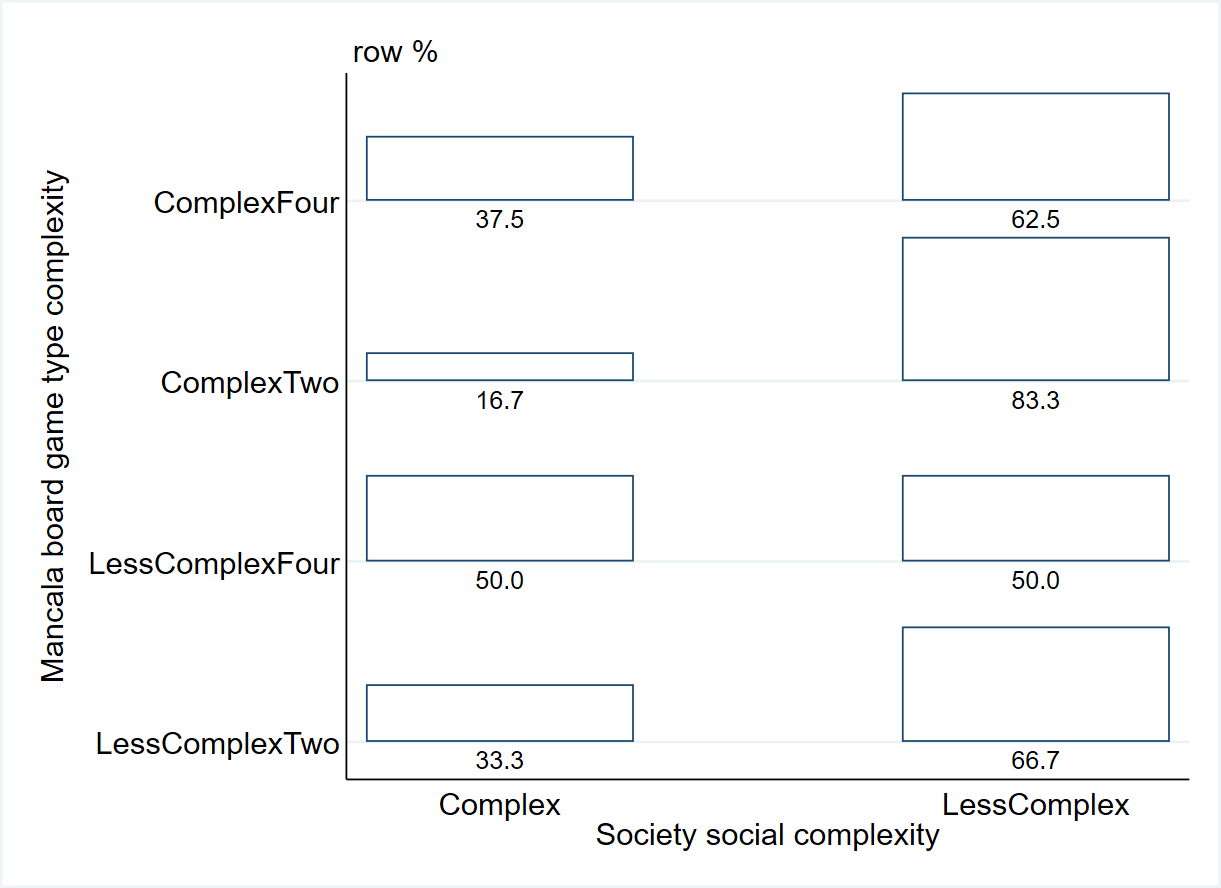

Supplement: S1 File — This zip file contains the underlying datasets, R code and the STATA do-file used to replicate the results of the manuscript. (ZIP) [file pone.0240790.s004.zip › replicationfiles/Graphs/GameComplexityandSocialCompl.png]

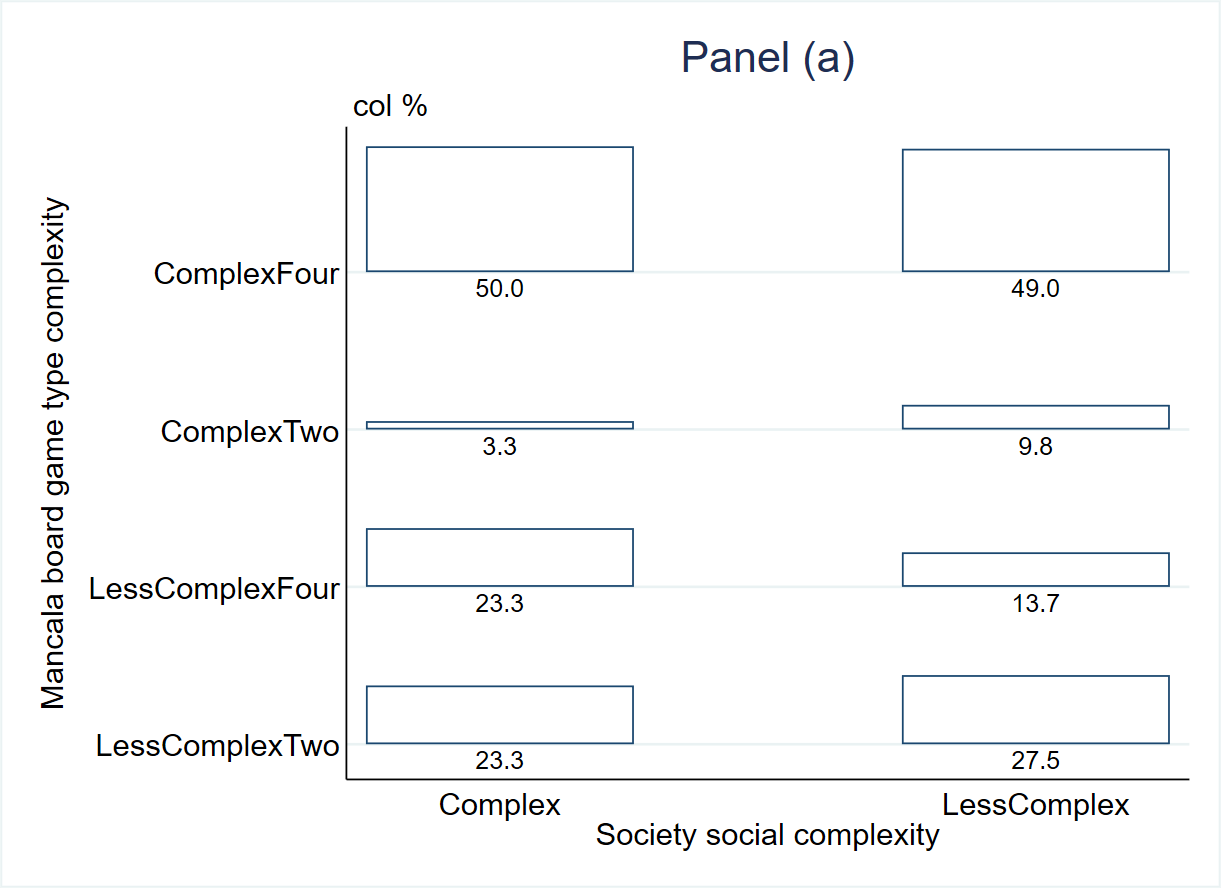

Supplement: S1 File — This zip file contains the underlying datasets, R code and the STATA do-file used to replicate the results of the manuscript. (ZIP) [file pone.0240790.s004.zip › replicationfiles/Graphs/GameComplexityandSocialCompl_Col.png]

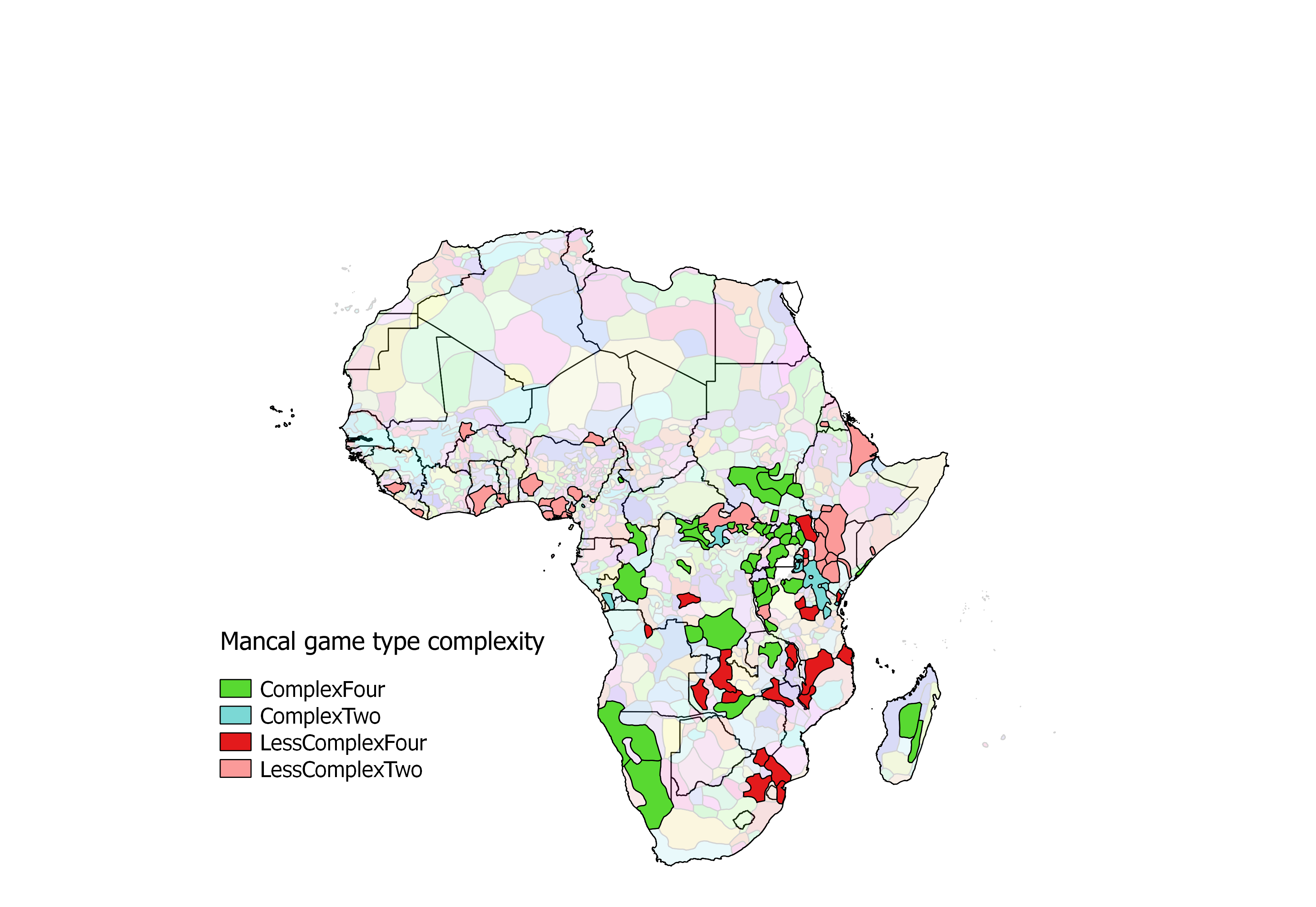

Supplement: S1 File — This zip file contains the underlying datasets, R code and the STATA do-file used to replicate the results of the manuscript. (ZIP) [file pone.0240790.s004.zip › replicationfiles/Graphs/GameComplexityMap.png]

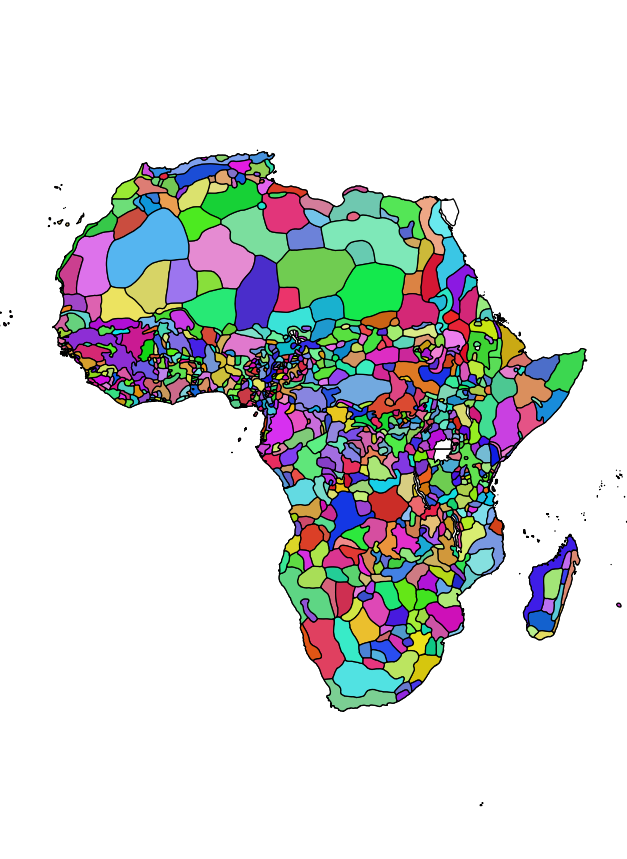

Supplement: S1 File — This zip file contains the underlying datasets, R code and the STATA do-file used to replicate the results of the manuscript. (ZIP) [file pone.0240790.s004.zip › replicationfiles/shp/Tribes.png]

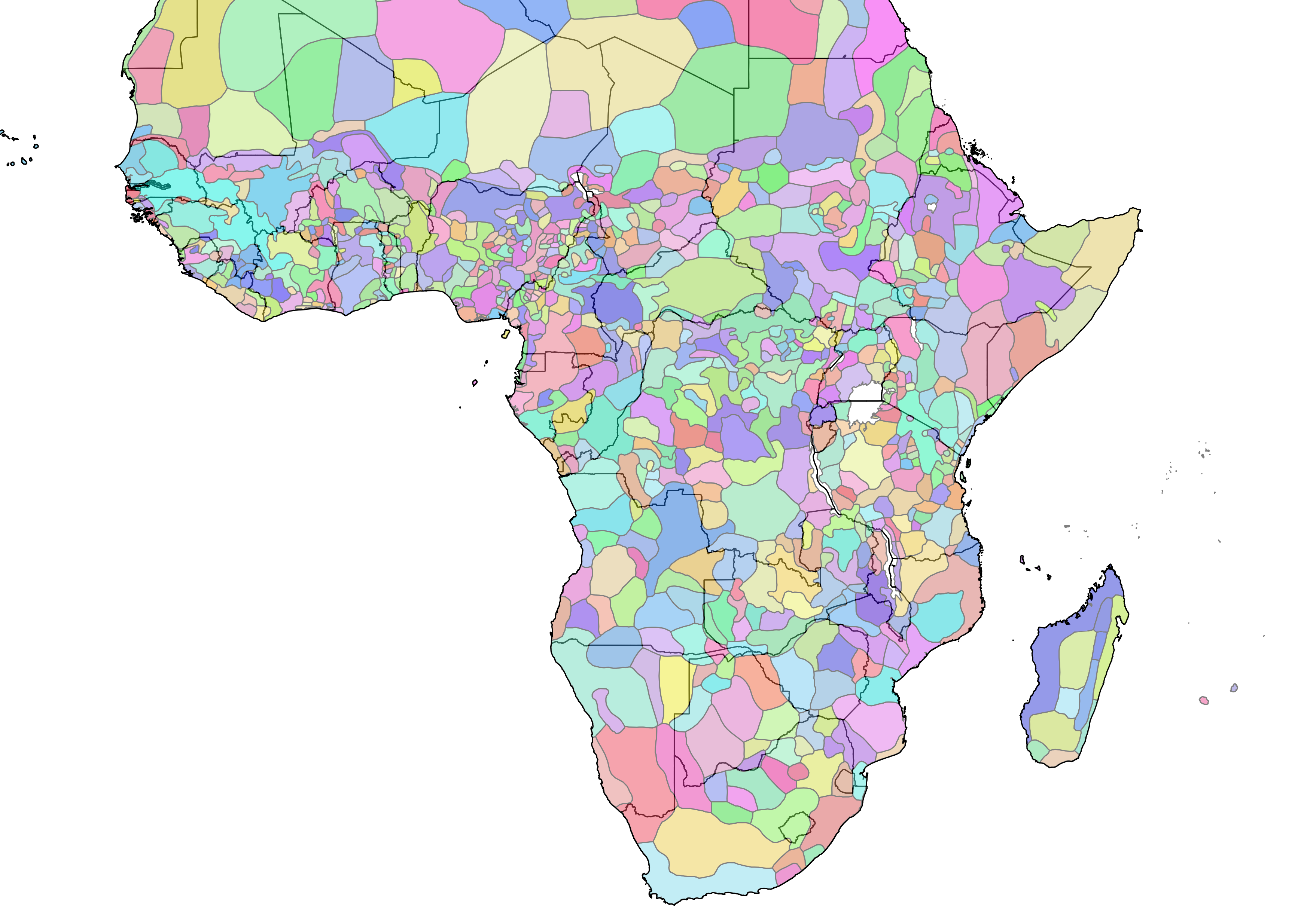

Supplement: S1 File — This zip file contains the underlying datasets, R code and the STATA do-file used to replicate the results of the manuscript. (ZIP) [file pone.0240790.s004.zip › replicationfiles/shp/Tribes2.png]

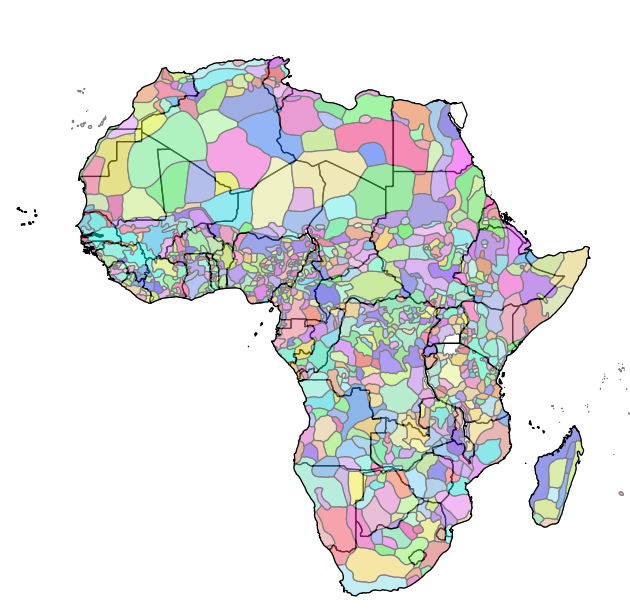

Supplement: S1 File — This zip file contains the underlying datasets, R code and the STATA do-file used to replicate the results of the manuscript. (ZIP) [file pone.0240790.s004.zip › replicationfiles/shp/Tribes3.png]
